# Diterpenoids of Marine Organisms: Isolation, Structures, and Bioactivities

**DOI:** 10.3390/md23030131

**Published:** 2025-03-18

**Authors:** Qi Shi, Shujie Yu, Manjia Zhou, Peilu Wang, Wenlong Li, Xin Jin, Yiting Pan, Yunjie Sheng, Huaqiang Li, Luping Qin, Xiongyu Meng

**Affiliations:** 1 School of Pharmaceutical Sciences, Zhejiang Chinese Medical University, 548 Binwen Road, Binjiang District, Hangzhou 310053, China; mikaelforget@163.com (Q.S.); rwnbiad_ysj@163.com (S.Y.); zmjsama@126.com (M.Z.); wangpeilu27@163.com (P.W.); liwl9902@163.com (W.L.); 15905853376@139.com (X.J.); pyt600050000@163.com (Y.P.); belemi123@163.com (Y.S.); 2 College of Pharmacy, Shaanxi University of Chinese Medicine, Xianyang 712046, China

**Keywords:** diterpenoid, marine fungi, structural diversity, biological activities

## Abstract

Diterpenoids from marine-derived organisms represent a prolific source of secondary metabolites, characterized by their exceptionally promising chemical structures and pronounced pharmacological properties. In recent years, marine diterpenoids have garnered considerable attention and are regarded as a prominent area of scientific research. As a vital class of metabolites, diterpenoids show diverse biological activities, encompassing antibacterial, antifungal, antiviral, anti-inflammatory, inhibitory, and cytotoxic activities, among others. With the rapid advancement of equipment and identified technology, there has been a tremendous surge in the discovery rate of novel diterpenoid skeletons and bioactivities derived from marine fungi over the past decade. The present review compiles the reported diterpenoids from marine fungal sources mainly generated from January 2000 to December 2024. In this paper, 515 diterpenoids from marine organisms are summarized. Among them, a total of 281 structures from various fungal species are included, comprising 55 from sediment, 39 from marine animals (predominantly invertebrates, including 17 from coral and 22 from sponges), and 53 from marine plants (including 34 from algae and 19 from mangrove). Diverse biological activities are exhibited in 244 compounds, and among these, 112 compounds showed great anti-tumor activity (45.90%) and 110 metabolites showed remarkable cytotoxicity (45.08%). Furthermore, these compounds displayed a range of diverse bioactivities, including potent anti-oxidant activity (2.87%), promising anti-inflammatory activity (1.64%), great anti-bacterial activity (1.64%), notable anti-thrombotic activity (1.23%), etc. Moreover, the diterpenoids’ structural characterization and biological activities are additionally elaborated upon. The present critical summary provides a comprehensive overview of the reported knowledge regarding diterpenoids derived from marine fungi, invertebrates, and aquatic plants. The systematic review presented herein offers medical researchers an extensive range of promising lead compounds for the development of marine drugs, thereby furnishing novel and valuable pharmaceutical agents.

## 1. Introduction

The ocean contains a vast array of biological resources, and marine natural products present unparalleled structural diversity and novelty relative to terrestrial sources [1]. Among these, it’s worth noting that diterpenoids represent one of the most crucial classes of terpenoids and demonstrate superexcellent physiological activities [2]. Meanwhile, the most abundant diterpenoids are found in the ocean. In recent years, an increasing number of research studies have focused on discovering novel diterpenes from marine-derived fungi [3,4].

**Figure 1:**
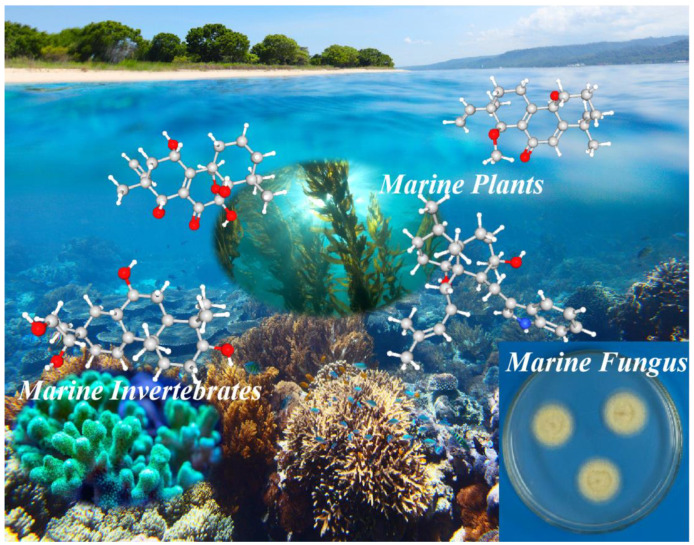
Sources of marine diterpenoids include marine fungi, marine invertebrates, and marine plants.

**Figure 2:**
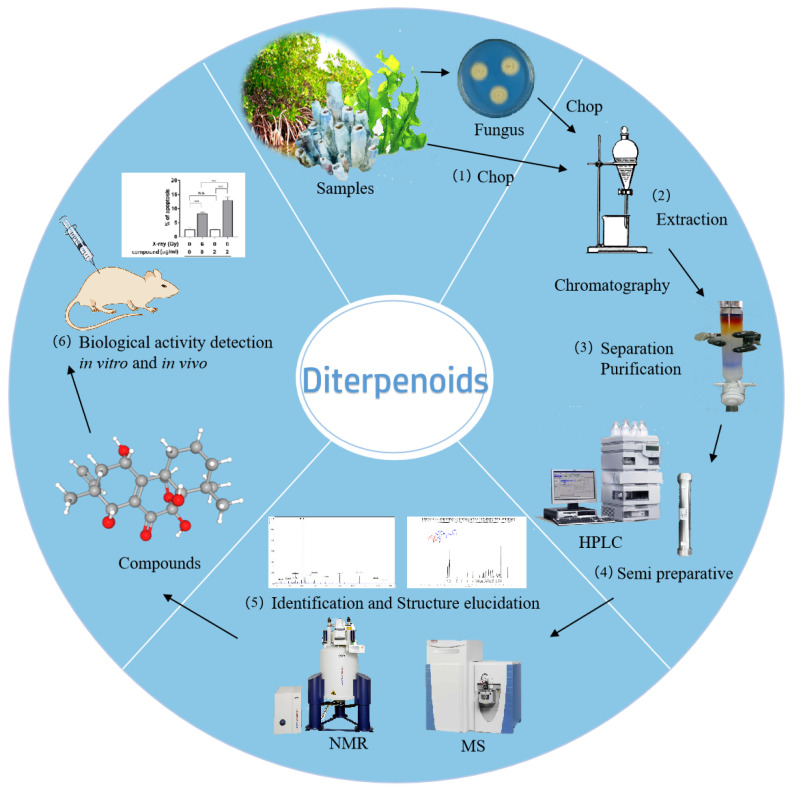
The separation process of compounds: (1) Biological sample collection; (2) Extract sample with PE/CH2Cl2/EtOAc/n-BuOH/MeOH; (3) Sample separation and purification; (4) Sample semi-preparation by HPLC; (5) Raw data acquisition and data analysis employing LC-MS, NMR, and HPLC; (6) Structure elucidation and bioactivity assay.

Herein, we have provided a comprehensive overview of marine diterpenoids, mainly covering the time from 2000 up to 2024 based on source classification, focusing on diterpenoids isolated from marine fungi. In total, 515 diterpenoid chemical structures are encompassed in this review, accompanied by an in-depth discussion of their bioactivities. By collecting information about their biological activities, pharmacologists are empowered to efficiently and easily identify marine diterpenes as potential drug candidates. The reported literature search was conducted employing diverse publishers and databases, including PubMed, Web of Science, ScienceDirect, Google Scholar, SciFinder, Scopus, Elsevier, Wiley, SpringerLink, and ACS Publications, applying specific keywords (diterpenoid, diterpene, marine fungi, marine invertebrates, and marine plants). Meanwhile, for a well-structured and comprehensive review, the diterpenoid compounds are classified into four categories based on their origin: marine fungi, marine invertebrates, marine plants, and mangroves (Figure 1). It should be noted that marine animal-derived diterpenoids are only found in marine invertebrates; “Marine invertebrates” is therefore considered as the appropriate taxonomic category rather than “Marine animals”. The standard procedures for acquiring diterpenes from marine organisms typically involve sample collection, separation, purification, identification, and bioactivity evaluation (Figure 2).

## 2. Diterpenoids from Marine Fungi

**Figure 3:**
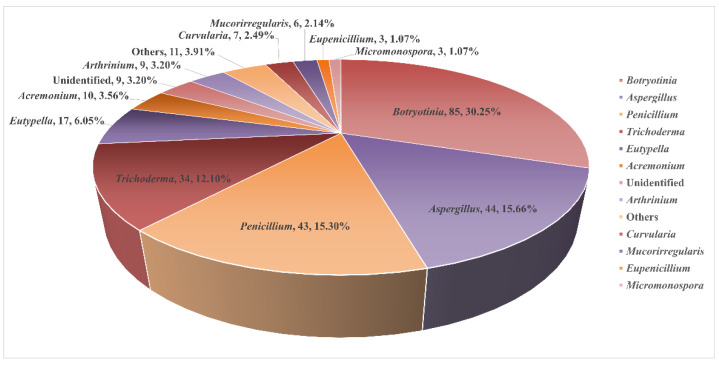
The diterpenoids from marine fungi in this review are divided by the origin of the genus.

Marine-sourced fungi are among the most prolific producers of bioactive natural products. Fungi have been demonstrated to be brilliant sources of bioactive compounds as potential sources of novel drugs [5,6]. As one of the richest producers of marine compounds, the diterpenes isolated from marine microorganisms account for nearly half in quantitative terms. The significant bioactive diversity exhibited by marine microorganisms has led to a wide array of natural compounds [7,8,9,10,11]. Given the scarcity of compounds sourced from marine bacteria, our review commences with an examination of diterpenoids derived from marine fungi. We summarized 286 compounds in this part, comprising 57 from sediment-sourced fungi, 39 from marine animal-sourced fungi (predominantly invertebrate-sourced, including 17 coral-sourced and 22 sponge-sourced), 37 from marine plants (algae)-derived fungi, and 19 from mangrove-derived fungi. If classified according to the fungal genus, the majority of diterpenoids are predominantly found in the genus *Botryotinia* (30.25%), *Aspergillus* (15.66%), *Penicillium* (15.30%), and *Trichoderma* (12.10%) (Figure 3). The bioactivities of these marine fungi-derived diterpenoids are elaborated in Table 1.

**Figure 4:**
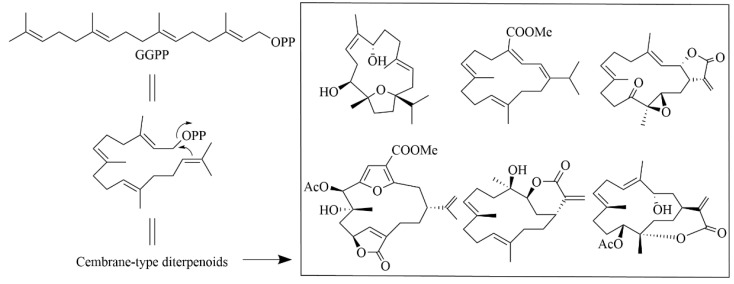
The cyclization mode and basic skeleton of the cembrane-type diterpenoids.

Marine fungi produce a diverse range of diterpenes with various carbon skeletons, typically consisting of four isoprene units linked in a “head-to-tail” manner, forming a carbon skeleton with 20 carbon atoms and representing an important category of marine terpenoids. Based on the latest research, these diterpenes can be classified into several types, including acyclic or monocyclic, bicyclic, tricyclic, tetracyclic, and more complex structures [12]. Among these, the cembrane-type diterpenes make up one of the largest groups, characterized by a 14-carbon ring skeleton containing five-, six-, seven-, or eight-membered lactone rings [12]. Figure 4 shows the cyclization mode and basic skeleton of the cembrane-type diterpenoids [13]. In addition, meroterpenoids, such as those isolated from marine soft coral-associated *Aspergillus* fungi with a 6/6/6 tricyclic skeleton, are also common and are derived through specific biosynthetic pathways (e.g., the DMOA pathway). Meanwhile, harziane-type diterpenoids possess a unique 6-5-4-7 tetracyclic carbon skeleton and are relatively rare in nature. These diterpenes are of significant interest due to their unique structural properties and biological activities. Future research will focus on analyzing the diversity of carbon skeletons and biosynthetic pathways of marine fungal diterpenoids to provide more comprehensive data support for related research fields.

### 2.1. Sediment-Sourced Fungi

As one of the most widespread sources of fungi, numerous diterpenoids have been discovered in marine sediments. This section provides a concise overview of 57 diterpenoids obtained from fungi isolated from sediment, showcasing their respective structures while emphasizing their profound biological activity.

#### 2.1.1. *Penicillium* sp.

**Figure 5:**
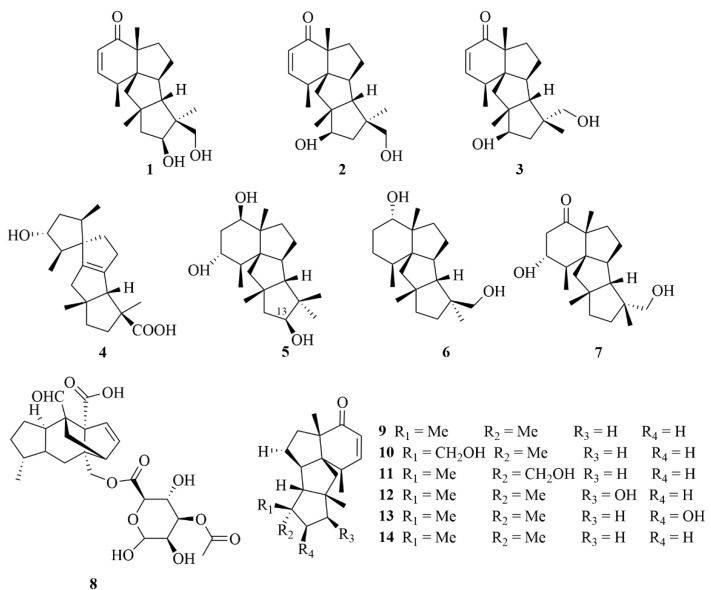
Chemical structures of diterpenoids from sediment-sourced fungi (**1**–**14**).

A study on the sea sediment-derived fungi *Penicillium* sp. TJ403-2 yielded three new diterpenoids identified as 13β-hydroxy conidiogenone C (**1**, Figure 5), 12β-hydroxy conidiogenone C (**2**, Figure 5), and 12β-hydroxy conidiogenone D (**3**, Figure 5) [14]. With an IC_50_ value of 2.19 ± 0.25 µM—threefold lower than the p.c. (positive control) indomethacin (IC_50_ = 8.76 µM)—compound 8 showed significant anti-inflammatory activity against LPS-induced NO production in RAW 264.7 cells [14]. In addition, 13β-hydroxy conidiogenone C (**1**) could strongly inhibit the production of cell cytokines, interleukin-1beta (IL-1β), interleukin-13 (IL-13), tumor necrosis factor-α (TNF-α), granulocyte-macrophage colony-stimulating factor (GM-CSF), macrophage inflammatory protein (MIP-1β), and monocyte chemoattractant protein-1 (MCP-1); suppress inducible nitric oxide synthesis (iNOS) and cyclooxygenase-2 (COX-2) protein expression in a dose-dependent manner; and abolish the nuclear translocation of nuclear factor-kappa B (NF-kB) p65 in LPS-activated RAW 264.7 cells [14]. As further study concluded its inhibition of the NF-kB-activated pathway, it is evident that compound 8 is a promising starting point for the development of new anti-inflammatory agents [14]. Another sea sediment-derived fungi, *Penicillium granulatum* MCCC 3A00475, produced a new spirotetracyclic diterpenoid with a 5/5/5/5 spiro-carbon skeleton structure, named spirograterpene A (**4**, Figure 5) [15]. It displayed anti-allergic effects on immunoglobulin E (IgE)-mediated rat mast RBL-2H3 cells with an inhibition rate of 18% at 20 µg/mL, and with loratadine serving as a positive control, it was 35% at the same concentration [15].

The isolation of three new cyclopiane diterpenoids, conidiogenols C and D (**5** and **6**, Figure 5) and conidiogenone L (**7**, Figure 5), was reported from *Penicillium* sp. YPGA11, a deep-sea-sediment fungi collected in the West Pacific Ocean at a depth of −4500 m [16]. The bioassay study proved the inhibitory effects against five esophageal HTCLs (EC109, KYSE70, EC9706, KYSE30, and KYSE450), in which compounds **5** and **7** showed weak inhibitory effects with inhibition rates less than 36% at an initial concentration of 50 µM [16]. Compound **6** showed more potent activity and was further tested for IC_50_ values. The IC_50_ values of compound **6** ranged from 36.80 to 54.7 µM (cisplatin as the p.c., 5.62 µM—7.96 µM), illustrating that it could exert moderate antiproliferative effects [16].

Xylarinonericin E (**8**, Figure 5), a novel glycosyl ester, was found in the fermentation broth of the fungi *Penicillium* sp. H1 from the sediments of the Beibu Gulf [17]. This compound displayed moderate antifungal activity against *Fusarium oxysporum f.* sp. Cubense with an MIC value of 32.0 µM/mL, and the MIC of the positive drug ketoconazole was 2.0 µM/mL [17].

A further study on the fungi *Penicillium* sp. F23-2 derived from deep-sea sediment demonstrated the isolation of six new diterpenoids, named conidiogenones B–G (**9**–**14**, Figure 5) [18]. The cytotoxic activities of all compounds were evaluated on HL-60, A-549, BEL-7402, and MOLT-4 cell lines [18]. Compounds **11**, **12**, and **14** showed notable cytotoxicity against the A-549 cell line with IC_50_ values of 9.3, 15.1, and 8.3 µM, respectively, while compounds **9** and **13** displayed much weaker cytotoxicity with IC_50_ values of 40.3 and 42.2 µM, respectively [18]. To the HL-60 cell line, compounds **11**, **12**, and **14** displayed potent cytotoxicity with IC_50_ values of 5.3, 8.5, and 1.1 µM, respectively, while compounds **9** and **13** showed weak activity with IC_50_ values of 28.2 and 17.8 µM, respectively [18]. It is worth mentioning that compound **10** showed ultra-high activity against the HL-60 and BEL-7402 cell lines, with IC_50_ values of 0.038 and 0.97 µM [18]. Compounds **11**, **13**, and **14** displayed moderate to weak cytotoxicity against the BEL-7402 cell line with IC_50_ values of 11.7, 17.1, and 43.8 µM, respectively [18]. In addition, they also showed biological activities against the MOLT-4 cell line with IC_50_ values of 21.1, 25.8, and 4.7 µM, respectively [18].

**Figure 6:**
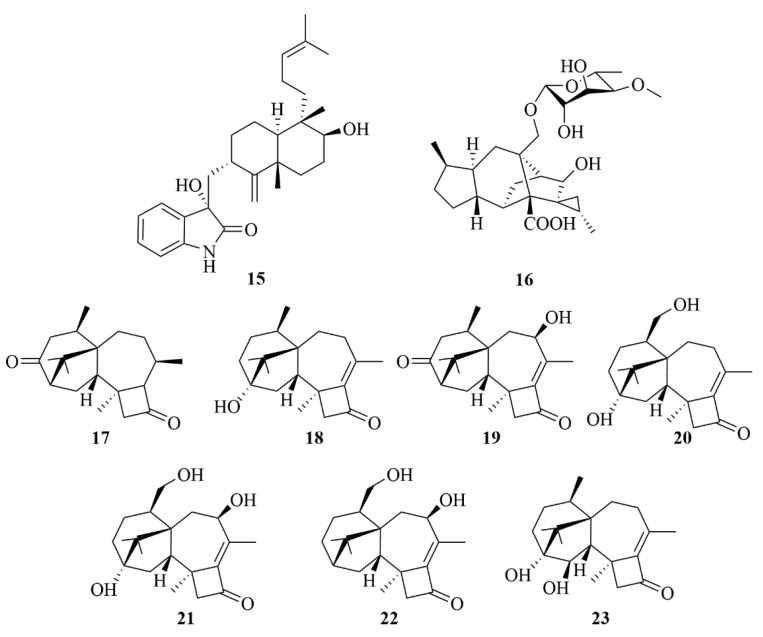
Chemical structures of diterpenoids from sediment-sourced fungi (**15**–**23**).

From the sea-sediment fungi *Penicillium* sp. YPCMAC1, collected at a depth of −4500 m in the western Pacific Ocean, penicindopene A (**15**, Figure 6), an indole diterpenoid containing a 3-hydroxy-2-indolone moiety, was isolated [19]. It displayed moderate cytotoxicity against the A-549 and HeLa cell lines with IC_50_ values of 15.2 and 20.5 µM, respectively [19].

#### 2.1.2. *Trichoderma* sp.

Trichosordarin A (**16**, Figure 6), a new sordarin derivative with a unique norditerpenoid aglycone, was discovered from *Trichoderma harzianum* R5, a deep-sea sediment-derived fungi collected in the Bohai Sea [20]. It is toxic to the marine zooplankton *A. salina* with an LC_50_ value of 233 µM. Still, it displayed weak inhibitory activity against two marine phytoplankton species (*Amphidinium carterae* and *Phaeocysti globosa*), with inhibition rates at 100 µg/mL of 20.6% and 8.1%, respectively [20].

Five new harziane-type diterpenoids were isolated from a deep-sea sediment-derived fungi *Trichoderma* sp. SCSIOW21, named harzianols K-O (**19**–**23**, Figure 6), along with two known compounds, hazianol J (**17**, Figure 5) and harzianol A (**18**, Figure 6) [21]. Harzianol J (**17**), harzianol A (**18**), and harzianol O (**23**) exhibited an anti-inflammatory effect with 81.8%, 46.8%, and 50.5% NO inhibition at 100 µM, respectively [21].

#### 2.1.3. *Aspergillus* sp.

**Figure 7:**
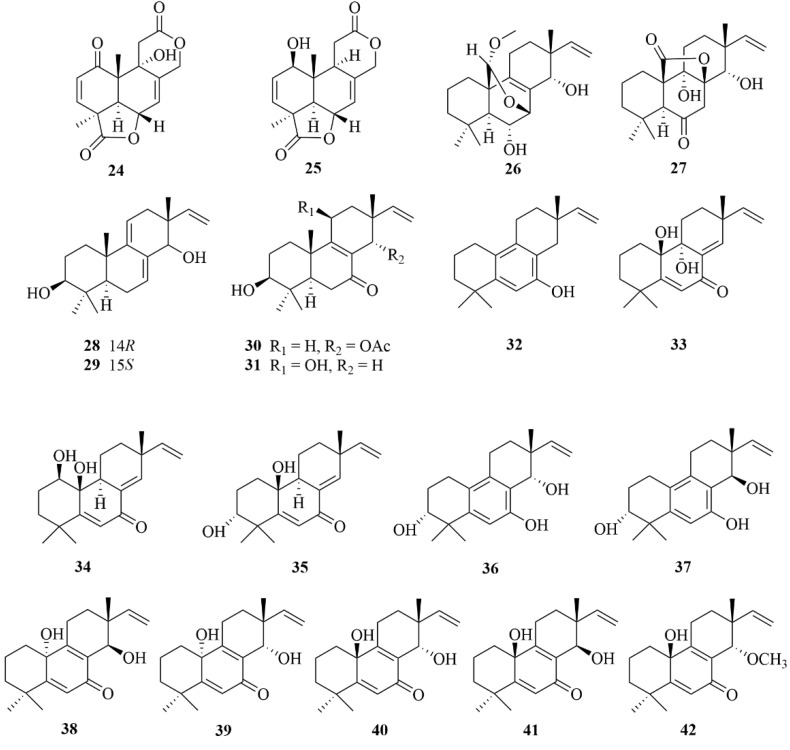
Chemical structures of diterpenoids from sediment-sourced fungi (**24**–**42**).

Further investigations reported nineteen diterpenoids (**24**–**42**, Figure 7) isolated from the marine sediment-derived fungi *Aspergillus wentii* SD-310, including two tetranorlabdane diterpenoids, asperolides D and E (**24** and **25**); six isopimarane-type diterpenoids, wentinoids A-F (**26**–**31**); ten undescribed rare 20-nor-isopimarane diterpenoid epimers, aspewentin A (**32**) and aspewentins D-L (**33**–**41**); and a new methylated derivative, aspewentin M (**42**) [22,23,24,25]. To the aquatic pathogens *Edwardsiella tarda*, compound **24** displayed moderate inhibitory activities with an MIC value of 16 µg/mL. At the same time, it is weaker against chloramphenicol and ampicillin with MIC values 8.0 and 2.0 µg/mL, respectively, which were used as positive controls [22]. Biological assay revealed the cytotoxicity of asperolides E (**25**) against HeLa, MCF-7, and NCI-H446 cell lines, with IC_50_ values of 10.0, 11.0, and 16.0 µM, respectively, and moderate activity against the *Edwardsiella tarda*, with an MIC value of 16 µg/mL [22]. Compound **24** and compounds **26**–**28** exhibited inhibitory activities against aquatic bacteria *Edwardsiella tarda*, *Micrococcus luteus*, *Pseudomonas aeruginosa*, *Vibrio harveyi*, and *V. parahemolyticus*, with the same MIC value of 4.0 µg/mL [22,23]. To the plant bacteria *Fusarium graminearum*, compounds **24** and **28** displayed substantial inhibitory activities with MIC values of 2.0 and 4.0 µg/mL, respectively, which were more potent than the positive control, amphotericin B, with an MIC value of 8.0 µg/mL [22,23]. The selective inhibition of wentinoid A (**26**) against four plants’ pathogenic fungi (*Phytophthora parasitica*, *Fusarium oxysporum f.* sp. lycopersici, *Fusarium graminearum*, and *Botryosphaeria dothidea*) proved that it may be a potential antifungal agent [23]. To aquatic pathogens (*M. luteus*, *E. tarda*, *V. harveyi*, *P. aeruginosa*, and *V. parahemolyticus*), compounds **33** and **35**–**38** offered remarkable inhibition, each with MIC values of 4.0 µg/mL, compared with the positive control chloramphenicol, with the MIC values of 8.0, 8.0, 4.0, 4.0, and 1.0 µg/mL, respectively [24]. Compounds **33** and **37** showed potent activity against plant pathogenic fungi *F. graminearum* with MIC values of 2.0 and 4.0 µg/mL, which showed more activity than the positive control (amphotericin B) with an MIC value of 8.0 µg/mL [24].

Furthermore, the bioactive test for brine shrimp lethality against *Artemia salina* showed that these compounds have no appreciable activity (LD_50_ > 10 µg/mL) [22,23,24,25]. In addition, an activity test showed that compound **38** has biological activity toward *E. coli* with an MIC value of 32 µg/mL. In contrast, aspewentins I and G (**38** and **39**) showed notable inhibitory activities against three marine bacteria (*E. tarda*, *V. harveyi*, and *V. parahaemolyticus*), with the same MIC value of 8.0 µg/mL [25]. In addition, compounds **38** and **39** displayed inhibitory activities toward zoonotic pathogens between human and aquatic animals, such as *Escherichia coli*, *Edwardsiella tarda*, *Vibrio harveyi*, and *V. parahaemolyticus* [25]. Thus, aspewentin M (**42**) may prove to be a beneficial antifungal agent for its potent antimicrobial activities against some plant pathogenic fungi (*Fusarium graminearum*, etc.), and compound **42** exhibited activity against *F.graminearum* with an MIC value of 4.0 µg/mL, which was the same MIC as for the p.c. amphotericin B. Thus, compounds with an *R* absolute configuration at C-10 are more active than those with an *S* configuration, for compounds **38** and **39** were more active against pathogenic bacteria than compounds **40**–**42** [25]. However, compound **42** displayed more muscular inhibitory activities of *F. graminearum* with methoxylation at C-14 than compounds **38**–**41** [25].

#### 2.1.4. *Eutypella* sp.

**Figure 8:**
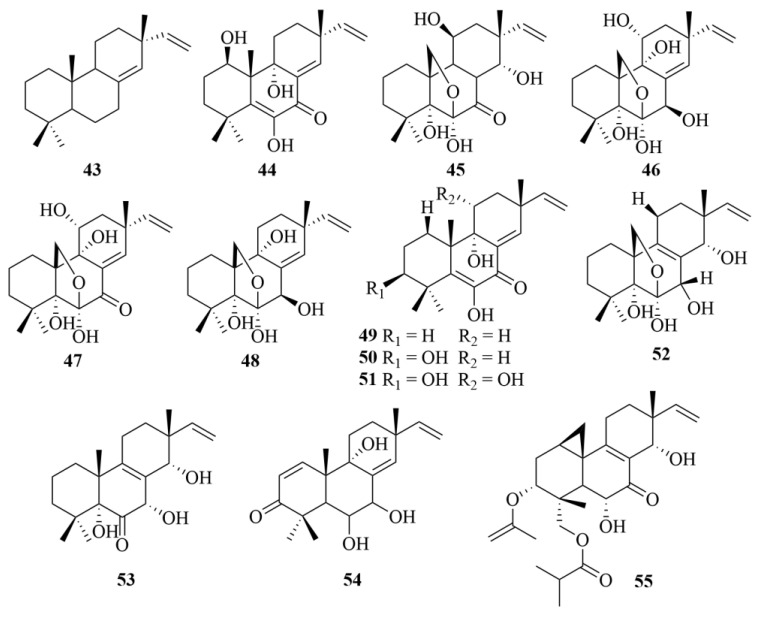
Chemical structures of diterpenoids from sediment-sourced fungi (**43**–**55**).

The sea-sediment-derived fungal strain from the South China Sea, *Eutypella scoparia*, produced six bioactive pimarane-type diterpenoids (**43**–**48**, Figure 8), identified as isopimara-8(14),15-diene (**43**), libertellenone A (**44**), scopararane B (**45**), diaporthein A (**46**), diaporthein B (**47**), and 11-deoxydiaporthein A (**48**) [26]. The bioactive test on SF-268 (human glioma cell line), MCF-7 (human breast adenocarcinoma cell line), and NCI-H460 (human non-small cell lung cancer cell line) revealed the selective cytotoxic activities of compound **44** (IC_50_ = 20.5, 12.0, and 40.2 µM), and the significant cytotoxicity of compound **47** (IC_50_ = 9.2, 4.4, and 9.9 µM), while compounds 45, 50, and 52 only showed its moderate cytotoxicity against the MCF-7 cell line with IC_50_ values of 38.8, 16.4, and 21.8 µM, respectively [26]. However, none of the other diterpenoids displayed any activities toward the three cell lines [26].

A recent study of *Eutypella scoparia* FS26, a marine-derived fungi from the sediment of the South China Sea, led to the isolation of five unprecedented oxygenated pimarane diterpenoids, named scopararanes C-G (**49**–**53**, Figure 8) [27]. Among them, scopararane C (**49**) and scopararane D (**50**) showed cytotoxic activities towards the MCF-7 cell line with IC_50_ values of 35.9 and 25.6 µM, respectively, while compounds **51** and **53** exhibited much weaker cytotoxicity with IC_50_ values of 74.1 µM and 85.5 µM, respectively [27]. To SF-268 and NCI-H460 cell lines, compound **50** displayed moderate cytotoxicity with IC_50_ values of 43.5 µM and 46.1 µM, respectively, while other diterpenoids did not exhibit any activities [27].

Two pimarane-type diterpenoids, scopararane H (**54**, Figure 8) and scopararane I (**55**, Figure 8), were first obtained from a deep-sea sediment-derived fungi *Eutypella* sp. FS46 (at a depth of −292 m) [28]. Scopararane I (**55**) exhibited moderate inhibitory activities against MCF-7, NCI-H460, and SF-268 cell lines with IC_50_ values of 83.91, 13.59, and 25.31 µg/mL, respectively [28].

### 2.2. Marine Invertebrates-Sourced Fungi

Abundant quantities of diterpenoids have been isolated from endophytic fungi associated with marine animals, predominantly derived from invertebrates such as corals and sponges. This section lists a compilation of 17 diterpenoids identified in corals and 22 diterpenoids found in sponges.

#### 2.2.1. Coral-Sourced Fungi

**Figure 9:**
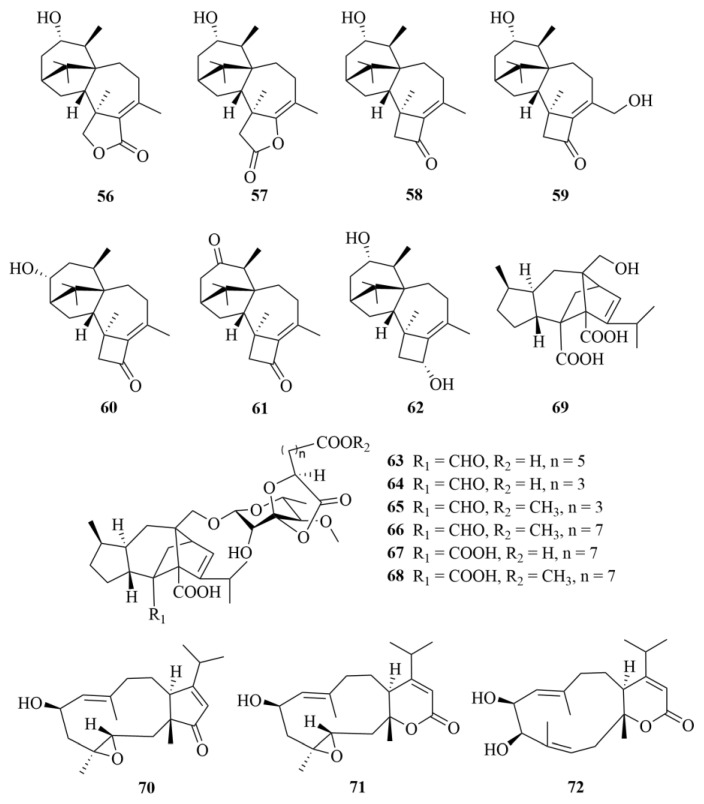
Chemical structures of diterpenoids from coral-sourced fungi (**56**–**72**).

*Trichoderma harzianum* XS20090075, a fungus derived from soft coral, produced seven novel harziane diterpenoids, including harzianelactones A and B (**56** and **57**, Figure 9), both with a 6/5/7/5-fused carbocyclic core containing a lactone ring system, and harzianones A-D (**58**–**61**, Figure 9) and harziane (**62**, Figure 9) [29]. These compounds are evaluated for phytotoxicity and all displayed notable activities against seedling growth of amaranth and lettuce [29].

Six new sordarin tetracyclic diterpenoid glycosides, moriniafungusns B-G (**63**–**68**, Figure 9), and a new sordaricin tetracyclic diterpenoid, sordaricin B (**69**, Figure 9), were isolated from the fungi *Curvularia hawaiiensis* TA2615 derived from the Weizhou coral reefs in the South China Sea [30]. These compounds exhibited diverse antifungal activity, which indicates that the glycosyl moiety, the length of the aliphatic acid side chain, and C-2 carboxylic acid may have impacts on antifungal activity; for example, compound **66** showed its potent antifungal activity against *Candida albicans* ATCC10231 with an MIC value of 2.9 µM [30].

A study reported the isolation of three novel dolabellane-type diterpenoids from a coral-derived fungi *Stachybotrys chartarum* TJ403-SS6, named stachatranones A-C (**70**–**72**, Figure 9) [31]. Among them, stachatranone B (**71**) showed selective biological activities, for not only did it exhibit an inhibitory effect against *Acinetobacter baumannii* (MIC = 16 µg/mL), with amikacin and vancomycin used as positive controls (MIC = 2, 8 µg/mL), but it also displayed an inhibitory effect against *Enterococcus faecalis* (MIC = 32 µg/mL), with vancomycin serving as the positive control (MIC = 0.5 µg/mL) [31].

#### 2.2.2. Sponge-Sourced Fungi

**Figure 10:**
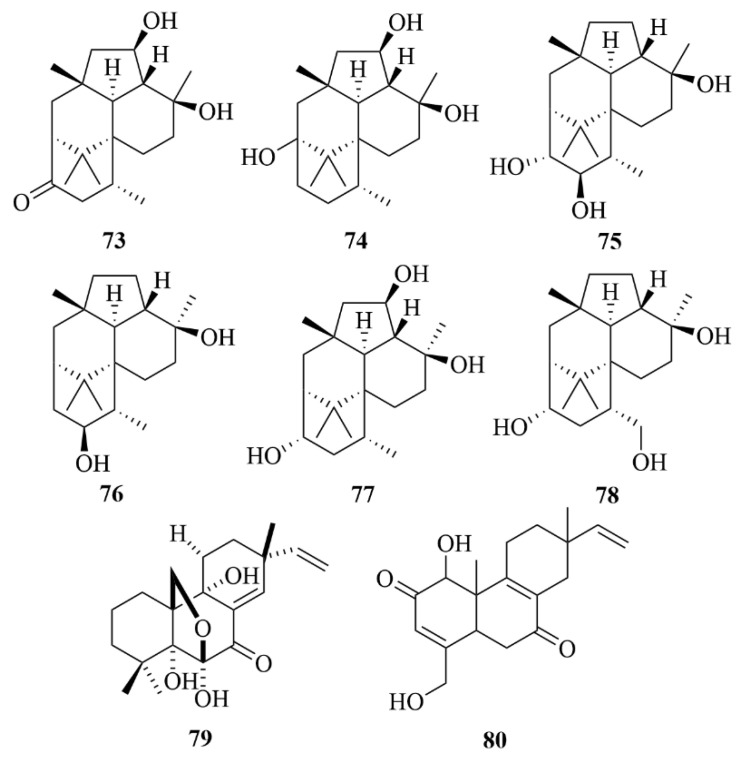
Chemical structures of diterpenoids from sponge-sourced fungi (**73**–**80**).

Trichodermanins C-H (**73**–**78**, Figure 10), six new diterpenoids that possess a fused 6-5-6-6 ring system, were obtained from the sponge-derived fungal strain *Trichoderma harzianum* OUPS-111D-4 from a piece of a marine sponge *Halichondria okadai* [32,33]. In the cytotoxicity assay towards three cancer cell lines, P388, HL-60, and L1210, trichodermanins C (**73**) exhibited potent activity with IC_50_ values ranging from 6.8 to 7.9 µM, and compounds **75** and **76** showed weaker activity, ranging from 41.5 to 125.2 µM, while the p.c. 5-fluorouracil displayed IC_50_ values ranging from 4.5 to 6.1 µM [32,33].

From the culture of the sea fungi *Cryptosphaeria eunomia var. Eunomia*, collected from a sponge growing off Pohnpei Island in the South Pacific, four pimarane-type diterpenoids were isolated, named 11-deoxydiaporthein A (**48**), diaporthein A (**46**), diaporthein B (**47**), and scopararane A (**79**, Figure 10) [34].

One unprecedented diterpenoid from the marine sponge-derived fungi *Actinomadura* sp. SpB081030SC-15 was reported, named compound JBIR-65 (**80**, Figure 10) [35]. It is the only compound that has been produced by the genus *Actinomadura* since 2009 [35]. The report demonstrated that compound JBIR-65 can protect neuronal hybridoma N18-RE-105 cells from L-glutamate toxicity with an EC_50_ value of 31 µM, but it is weaker than the representative antioxidant α-tocopherol with an EC_50_ value of 6.3 µM [35].

**Figure 11:**
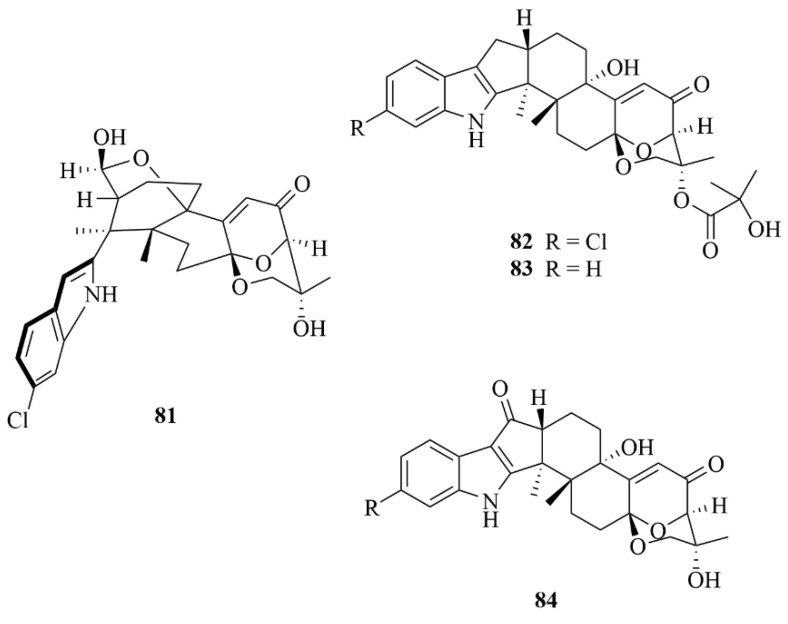
Chemical structures of diterpenoids from sponge-sourced fungi (**81**–**84**).

*Aspergillus candidus* HDN15-152, marine fungi derived from a sponge, produced four new indole diterpenoids and ascandinines A-D (**81**–**84**, Figure 11) [36]. Except for ascandinine A, the three compounds left all have rare 6/5/5/6/6/6/6 fused ring systems [36]. Bioactivity assay showed that compound **83** showed anti-influenza virus A (H1N1) activity with an IC_50_ value of 26 µM, with ribavirin serving as the positive control (IC_50_ = 31 µM). In comparison, compound **84** showed potent cytotoxic activity against HL-60 cells with an IC_50_ value of 7.8 µM [36].

**Figure 12:**
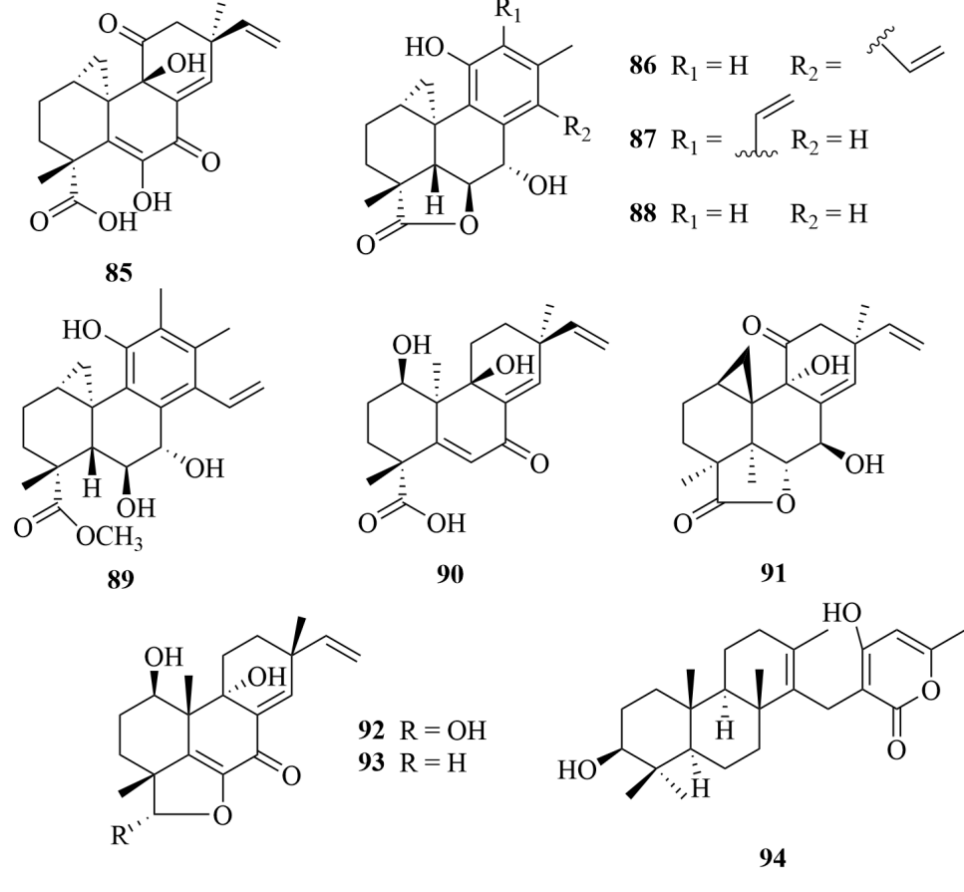
Chemical structures of diterpenoids from sponge-sourced fungi (**85**–**94**).

Various compounds were reported from the sponge-derived genus *Arthrinium*. Arthrinins A-D (**85**–**88**, Figure 12), myrocin D (**89**, Figure 12), and myrocin A (**90**, Figure 12) are six diterpenoids obtained from the marine sponge-derived fungi *Arthrinium* sp. [37]. Compounds **89** and **90** inhibited vascular endothelial growth factor A (VEGF-A)-dependent endothelial cell sprouting (IC_50_ = 2.6, 3.7 µM), with sunitinib used as a positive control (IC_50_ = 0.12 µM), which proved the antitumor activity of myrocin D [37]. To the L5178Y (mouse lymphoma) tumor cell line, it also exhibited notable antiproliferative activities (IC_50_ = 2.05, 2.74 µM), with kahalalide F serving as a positive control (IC_50_ = 4.30 µM) [37]. However, myrocin D (**89**) and myrocin A (**90**) showed no inhibitory activity for the protein kinase and weak activities against K-562, A2780 (human ovarian cancer line), and A2780CisR (cisplatin-resistant human ovarian cancer cells), with IC_50_ values of 50.3, 41.3, 66.0, and 42.0, 28.2, 154.7 µM, respectively, with cisplatin used as the positive control (IC_50_ = 7.80, 0.80, and 8.40 µM) [37].

Another type of fungi of the genus *Arthrinium*, *Arthrinium sacchari*, isolated three undescribed compounds, named myrocin D (**91**, Figure 12), libertellenone E (**92**, Figure 12), and libertellenone F (**93**, Figure 12) [38]. The result of the vitro angiogenesis assay on human umbilical vascular endothelial cell (HUVEC) sprouting induced by VEGF-A revealed that compounds **91** and **92** have no antitumoral potential [38].

It should be noted that two compounds isolated from different strains of *Arthrinium* by other researchers, compounds **89** and **91**, were named the same, myrocin D [37,38]. The close timing of submission and acceptance of the two articles is speculated to be the probable cause of the coincidence [37,38].

Investigation of *Neosartorya paulistensis*, a rare marine sponge-derived fungi, led to a meroditerpenoid, sartorypyrone C (**94**, Figure 12) [39]. It has no apparent antibacterial activity against four reference strains (*Staphylococcus aureus*, *Bacillus subtilis*, *Escherichia coli*, and *Pseudomonas aeruginosa*) [39].

### 2.3. Marine Algae-Derived Fungi

A plethora of bioactive compounds have recently been discovered in marine plants, showcasing their remarkable biological potential [40,41,42]. Fungi isolated from marine plants serve as another valuable reservoir of marine diterpenoids, with endophytic fungi from algae being the primary source. This section encompasses a comprehensive collection of 37 diterpenoids derived from endophytic algae-sourced fungi, including both red algae-sourced fungi and brown algae-sourced fungi.

#### 2.3.1. *Trichoderma* sp.

**Figure 13:**
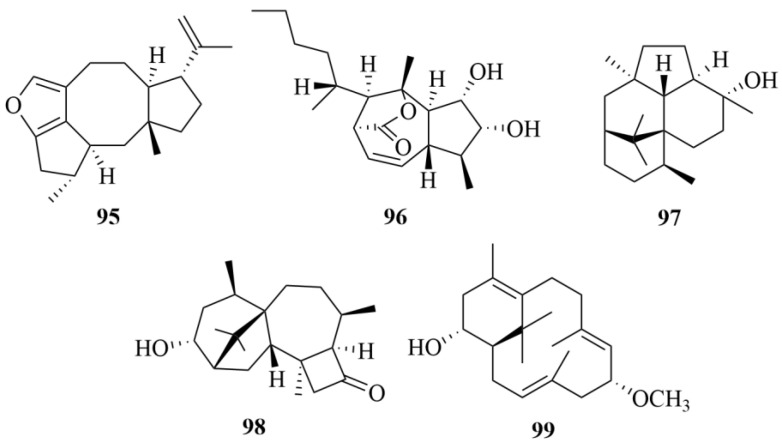
Chemical structures of diterpenoids from algae-sourced fungi (**95**–**99**).

The marine brown algae-derived fungus *Trichoderma citrinoviride* cf-27 has been found to produce a furan-bearing fusicoccane diterpenoid, trichocitrin (**95**, Figure 13), and a new norditerpenoid with an unprecedented skeleton, citrinovirin (**96**, Figure 13) [43,44]. To *E. coli*, compound **95** showed antibacterial activity with an inhibitory diameter of 8.0 mm at 20 µg/disc. At the same time, *Prorocentrum donghaiense* exhibited anti-microalgal solid capability with 54.1% growth inhibition at 80 µg/mL [43,44]. Compound **96** showed inhibitory activity towards *S. aureus* (MIC = 12.4 µg/mL), exhibited toxicity against the marine zooplankton *Artemia salina* (LC_50_ = 65.6 µg/mL), and displayed 14.1–37.2% inhibition of three marine phytoplankton species (*C. marina*, *H. akashiwo*, and *P. donghaiense*) at 100 µg/mL [43,44]. However, citrinovirin (**96**) is conducive to the growth of *Scrippsiella trochoidea*, a marine phytoplankton [43,44].

A new diterpenoid antipode, (+)-wickerol A (**97**, Figure 13), was discovered from *Trichoderma asperellum* d1-34, a fungus derived from marine brown algae [45]. The biologic investigation of compound **97** showed that it has inhibitory activity against *E. coli* and *S. aureus*, with the same inhibitory diameter of 8.0 mm at 30 µg/disc, and displayed lethal activity against *A. salina* with an LC_50_ value of 12.0 µg/mL [45].

*Trichoderma harzianum* X-5, a fungus derived from the marine brown algae Laminaria japonica, furnished two undescribed diterpenoids, named 3*R*-hydroxy-9*R*,10*R*-dihydroharzianone (**98**, Figure 13) and 11Rmethoxy-5,9,13-proharzitrien-3-ol (**99**, Figure 13) [46]. The biologic activities of two compounds were evaluated on four phytoplankton species (*Chattonella marina*, *Heterosigma akashiwo*, *Karlodinium veneficum*, and *Prorocentrum donghaiense*) [46]. Results demonstrated the inhibitory activity of compound **104** against Chattonella marina with an IC_50_ value of 7.0 µg/mL, and compound **105** showed a notable inhibitory effect on the growth of all four kinds of phytoplankton, with IC_50_ values of 1.2, 1.3, 3.2, and 4.3 µg/mL, respectively, with K_2_Cr_2_O_7_ as the positive control (IC_50_ = 0.46, 0.98, 0.89, and 1.9 µM) [46].

**Figure 14:**
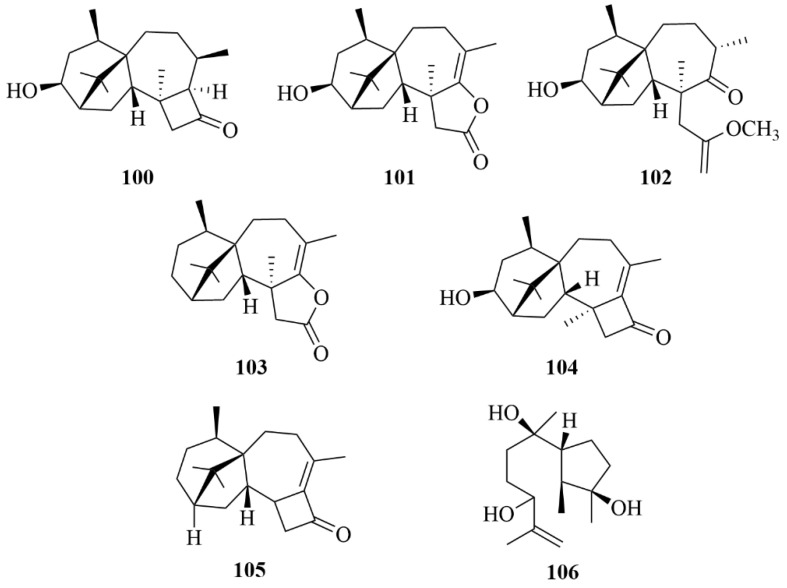
Chemical structures of diterpenoids from algae-sourced fungi (**100**–**106**).

Three novel harziane diterpenoids were found from the algicolous fungi *Trichoderma asperelloides* RR-dl-6-11, identified as 3*S*-hydroxy-9*R*,10*R*-dihydroharzianone, 3Shydroxytrichodermaerin, and methyl 3*S*-hydroxy-10,11-seco-harzianate (**100**–**102**, Figure 14) [47]. There is no inhibitory activity exhibited against any of the tested marine bacteria by these compounds at 100 µg/disc [47].

Deoxytrichodermaerin (**103**, Figure 14), an undescribed harziane lactone with an ester linkage between C-10 and C-11, was isolated from the cultivation of an endophyte, *Trichoderma longibrachiatum* A-WH-20-2, which was derived from the marine red algae *Laurencia okamurai* [48]. To four marine phytoplankton strains (*C. marina*, *H. akashiwo*, *K. veneficum*, and *P. donghaiense*), deoxytrichodermaerin (**103**) and the other two isolates (harzianol A and harzianone) showed strong inhibition, with IC_50_ values ranging from 0.53 to 2.7 µg/mL [48]. Compound 111 exhibited toxicity against the marine zooplankton *A. salina* with an LC_50_ value of 19 µg/mL, which confirmed that the lactone unit in deoxytrichodermaerin may have some contribution to these activities [48].

The isolation of one new harziane diterpenoid, 3*S*-hydroxyharzianone (**104**, Figure 14), which may be an intermediate in the biosynthesis of harziandione from harzianone, was concluded by an investigation of a marine red algae-derived endophytic fungus, *Trichoderma asperellum* A-YMD-9-2 [49]. The bioactive assay showed that compound **104** has a significant inhibition against three red tide-related phytoplankton species (*C. marina*, *H. akashiwo*, *K. veneficum*, and *P. donghaiense*) with IC_50_ values ranging from 3.1 to 7.7 µg/mL, and its inhibitory ability is primarily due to the hydroxyl group at C-3 [49]. In addition, compound **104** displayed weak inhibition against five marine-derived pathogenic bacteria (four different strains of *Vibrio* and a *P. citrea*), at 40 µg/disc [49].

Harzianone (**105**, Figure 14), a new harziane dieterpene, was obtained from a seaweed-derived fungus *Trichoderma longibrachiatum* [50]. It showed 82.6% lethality in brine shrimp (*Artemia salina* L.) larvae at 100 µg/mL [50]. Moreover, the antibacterial activity of harzianone was evaluated on *Escherichia coli* and *Staphylococcus aureus* at 30 µg/disk, with inhibitory diameters of 8.3 and 7.0 mm, respectively, while chloramphenicol as the positive control exhibited inhibitory diameters of 22 mm at 20 µg/disc [50].

Two fungal strains, *Trichoderma citrinoviride* cf-27 and *Trichoderma asperellum* cf44-2, were isolated from the surface of seaweed [43,51]. Among them, the fungus *Trichoderma citrinoviride* cf-27 was proven to be the source of a new diterpenoid, named trichocitrin (**95**) [43,51]. Trichocitrin formed an 8.0 mm inhibition zone against *Escherichia coli* at 20 µg/disk [43,51]. In addition, the isolation of one novel compound, 11-hydroxy-9-harzien-3-one (**106**, Figure 14), was reported from the fermentation of the fungus *Trichoderma asperellum* cf44-2 [43,51].

#### 2.3.2. *Aspergillus* sp.

**Figure 15:**
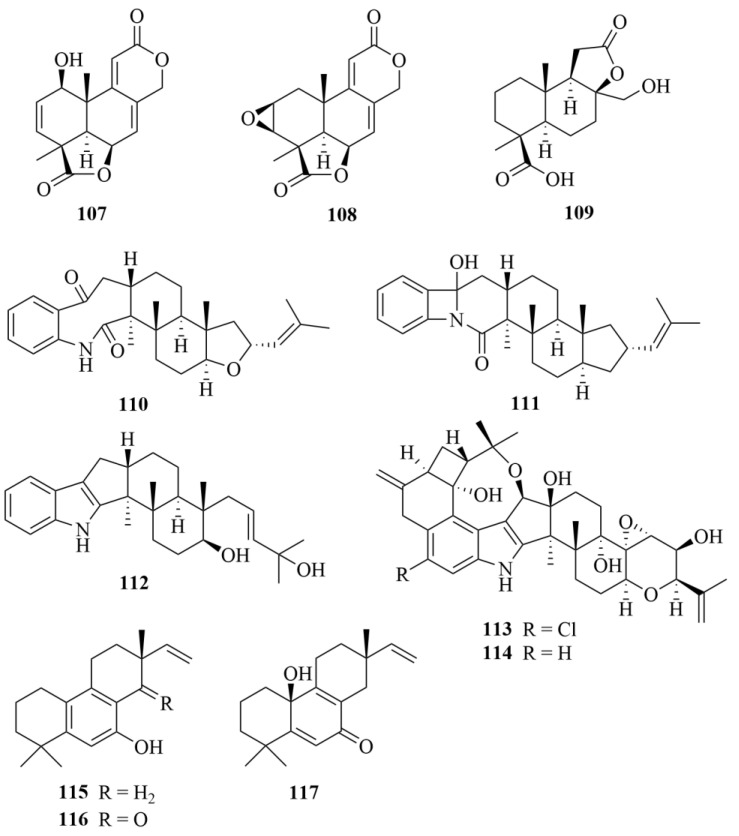
Chemical structures of diterpenoids from algae-sourced fungi (**107**–**117**).

From the brown algal-derived fungus *Aspergillus wentii* EN-48, three new norditerpenoids, asperolides A-C (**107**–**109**, Figure 15), were acquired [52]. No bioactivity was reported [52,53].

From the red algae-derived fungus *Aspergillus oryzae*, two new indole diterpenoid derivatives asporyzins A and B (**110** and **111**, Figure 15), one new indole diterpenoid asporyzin C (**112**, Figure 15), and three known related indole diterpenoids were discovered [54]. However, the three new compounds **110**–**112** did not exhibit any antibacterial activity against *Escherichia coli* or antifungal activity against plant pathogens *Colletotrichum lagenarium* and *Fusarium oxysporum* [54].

The team of Zhang et al. reported the isolation of two undescribed indole diterpenoids derived from a red algae-derived fungus *Aspergillus nidulans* EN-330, named 19-hydroxypenitrem A (**113**, Figure 15) and 19-hydroxypenitrem E **114**, Figure 15) [55]. Compound **113** exhibited antibacterial activity against pathogens *Edwardsiella tarda*, *Vibrio anguillarum*, *Escherichia coli*, and *Staphylococcus aureus*, with MIC values of 16, 32, 16, and 16 µg/mL, respectively, with chloramphenicol used as the positive control (MIC = 16, 0.5, 2, and 2 µg/mL) [55].

The fermentation of the fungus *Aspergillus wentii* na-3, a fungus derived from the surface of Sargassum algae, was the source of three novel norditerpenoids (**115**–**117**, Figure 15) [56]. In the assay of inhibitory activity, compound **116** exhibited inhibitory activities against the marine zooplankton *Artemia salina* with an LC_50_ of 6.36 µM, and compound **115** showed activity against two marine phytoplankton species (*Chattonella marina* and *Heterosigma akashiwo*), with LC_50_ values of 0.81 and 2.88 µM, respectively [56].

#### 2.3.3. *Penicillium* sp.

**Figure 16:**
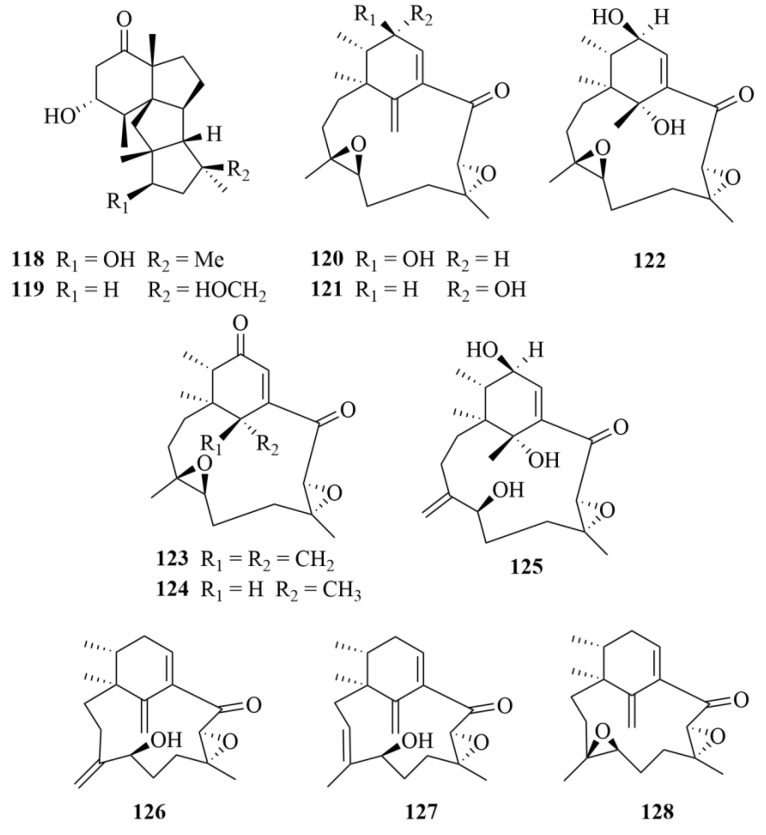
Chemical structures of diterpenoids from algae-sourced fungi (**118**–**128**).

Two unusual diterpenoids, cyclopiasconidiogenones H and I (**118** and **119**, Figure 16), were isolated by Gao et al. from a red algae-derived fungus *Penicillium chrysogenum* QEN-24S [57]. No bioactivity of the two compounds was reported in the antimicrobial test [57].

#### 2.3.4. Unidentified Fungi

There were nine new diterpenoids found from a marine red algal-derived unidentified fungus, including phomactin I (**120**, Figure 16), 13-epiphomactin I (**121**, Figure 16), phomactin J (**122**, Figure 16), phomactins K-M (**123**–**125**, Figure 16), and phomactins N-P (**126**–**128**) [53,58]. These compounds were tested for cytotoxicity against HUVECs, NHDF (normal human dermal fibroblasts) cells, and HeLa cells, but they did not show any activity [53,58].

### 2.4. Mangrove-Derived Fungi

Mangroves thrive in seawater, typically found at the confluence of terrestrial and ocean mudflats. Due to their unique growth habit, mangroves are classified into a separate category. This section summarizes 19 diterpenoids isolated from fungi originating from mangroves.

**Figure 17:**
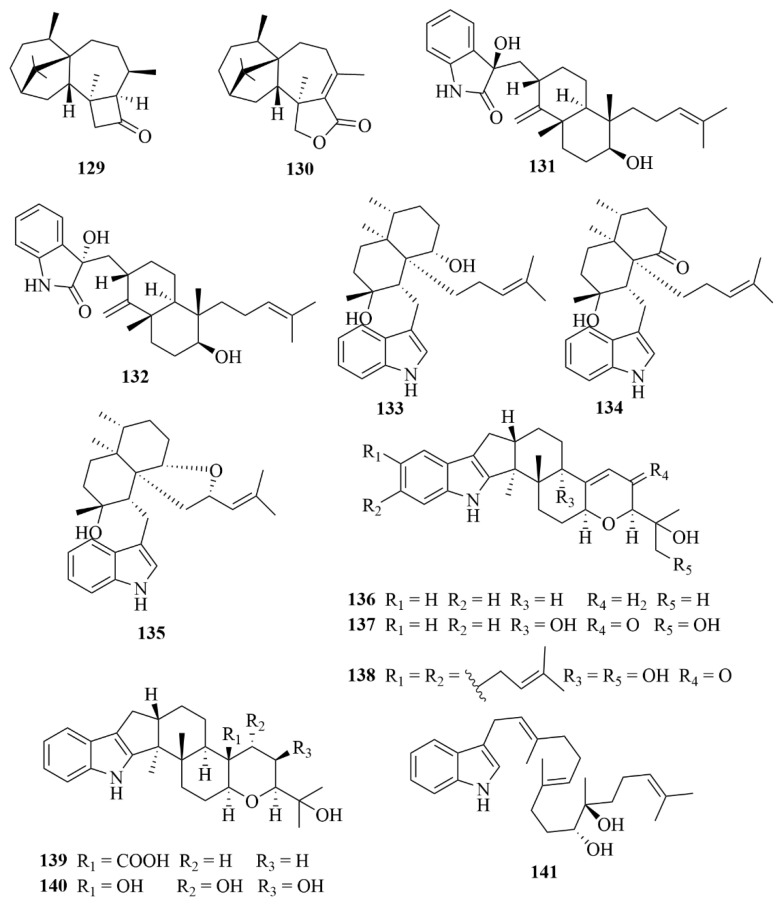
Chemical structures of diterpenoids from mangrove-sourced fungi (**129**–**141**).

A study on the marine mangrove-derived endophytic fungus *Trichoderma* sp. Xy24 led to the discovery of two novel harziane diterpenoids, (9*R*, 10*R*)-dihydro-harzianone (**129**, Figure 17) and harzianelactone (**130**, Figure 17) [59]. To the HeLa and MCF-7 cell lines, compound **129** showed selective cytotoxicity with IC_50_ values of 30.1 and 30.7 µM, respectively, whereas compound **130** was inactive at 10 mM [59].

*Aspergillus versicolor*, a fungus derived from marine mangroves, produced two new oxoindolo diterpenoids, anthcolorin G (**131**, Figure 17) and anthcolorin H (**132**, Figure 17) [60]. Compound **132** exhibited weak activity against HeLa cells, with an IC_50_ value of 43.7 µM [60].

The mangrove-derived fungus *Eupenicillium* sp. HJ002 resulted in the isolation and identification of three new indole diterpenoids, penicilindoles A-C (**133**–**135**, Figure 17) [61]. The bioactivities of compounds 138–140 were evaluated on human A-549, HeLa, and HepG2 cell lines by the MTT method [62]. Among them, compound **133** exhibited potent activities against human A-549 and HepG2 cell lines (IC_50_ = 5.5, 1.5 µM), with adriamycin used as the positive control (IC_50_ = 0.002, 0.1 µM), and 36.8 and 76.9 µM, respectively, for 5-fluoracil [62].

The fungus *Penicillium camemberti* OUCMDZ-1492, which was isolated from the culture of marine mangroves, afforded six novel indole diterpenoids (**136**–**141**, Figure 17) [61]. Against the H1N1 virus, weak activities were exerted by compounds **136**–**138** and **140**, with IC_50_ values of 28.3, 38.9, 32.2, and 73.3 µM, respectively [61].

**Figure 18:**
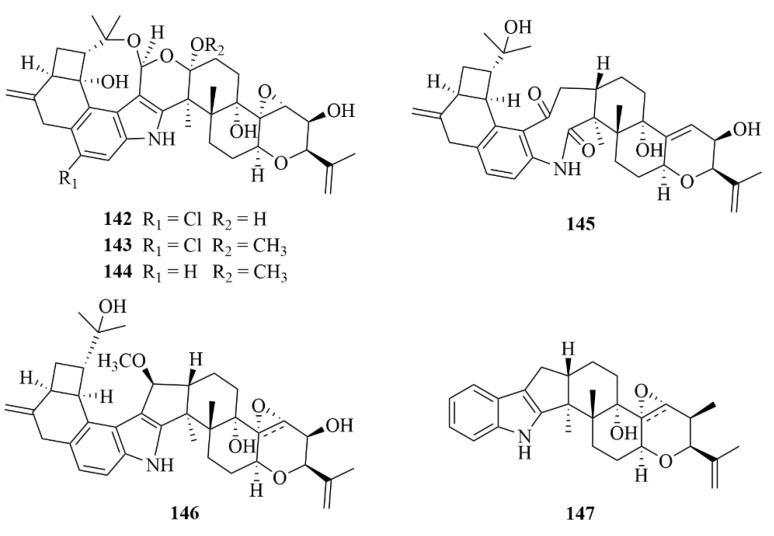
Chemical structures of diterpenoids from mangrove-sourced fungi (**142**–**147**).

Gao et al. discovered six indole diterpenoids from the fungal strain *Mucorirregularis* QEN-189 isolated from mangroves, named rhizovarins A–F (**142**–**147**, Figure 18) [63]. With IC_50_ values of 11.5, 6.3, and 9.2 µM, respectively, compounds **142**, **143**, and **147** exhibited moderate activities toward the A-549 cancer cell line, with adriamycin serving as the positive control (IC_50_ = 0.3 µM) [63]. Additionally, compounds **142** and **143** showed notable activities against the HL-60 cancer cell line with IC_50_ values of 9.6 and 5.0 µM, respectively, compared to adriamycin (IC_50_ = 0.067 µM) [63].

### 2.5. Miscellaneous

In addition to marine sediments, marine invertebrates, marine plants, and mangroves, a few other sources of fungi have also been found to contain diterpenoids, such as seawater, marine ascidians, and sea anemones. However, their sources are limited in quantity and have not been classified separately. Furthermore, some compounds obtained from marine sources have not been clearly described and are included in this section.

#### 2.5.1. *Penicillium* sp.

**Figure 19:**
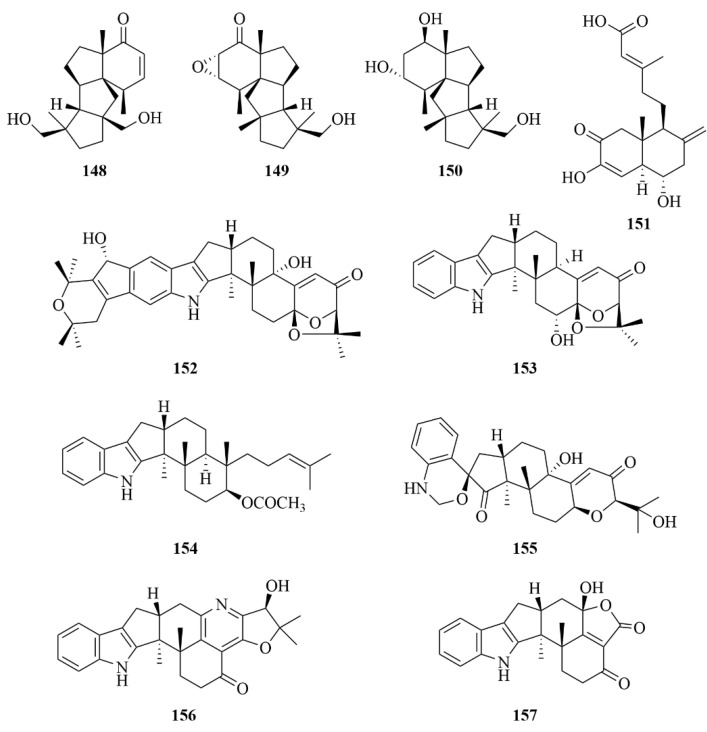
Chemical structures of diterpenoids from other-sourced fungi (**148**–**157**).

Three undescribed cyclopiane diterpenoids exhibiting a rare rigid 6/5/5/5 fused tetracyclic ring framework were isolated from the deep-sea fungi *Penicillium commune* MCCC 3A00940, including conidiogenone K (**149**, Figure 19), conidiogenol B (**150**, Figure 19), and the first naturally occurring cyclopiane diterpenoid enantiomer, conidiogenone J (**148**) [64]. However, when tested for antiallergic effects in immunoglobulin E (IgE)-mediated rat basophilic leukemia RBL2H3 cells, none of these compounds showed biological activity [64].

An unusual 19-nor labdane-type diterpenoid, named penitholabene (**151**, Figure 19), was obtained from the marine fungal strain *Penicillium thomii* YPGA3, which was derived from the deep-sea water at a depth of −4500 m in the Yap Trench (West Pacific Ocean) [65]. This compound was confirmed to be the first 19-nor labdane-type diterpenoid found in nature [65]. To the α-glucosidase, it displayed an inhibitory effect, with an IC_50_ value of 282 µM, which was more active than the positive control, acarbose (1330 µM) [65].

*Penicillium* sp. AS-79, a fungus derived from the sea anemone, produced three novel indole diterpenoids, named 22-hydroxylshearinine F (**152**, Figure 19), 6-hydroxylaspalinine (**153**, Figure 19), and 7-O-acetylemindole SB (**154**, Figure 19) [66]. In the evaluation of bioactivity, compound **153** exhibited activity against the aquatic pathogen *Vibrio parahaemolyticus* with an MIC of 64.0 µg/mL, compared to the positive control chloromycetin, with the MIC value of 0.5 µg/mL [66].

**Figure 20:**
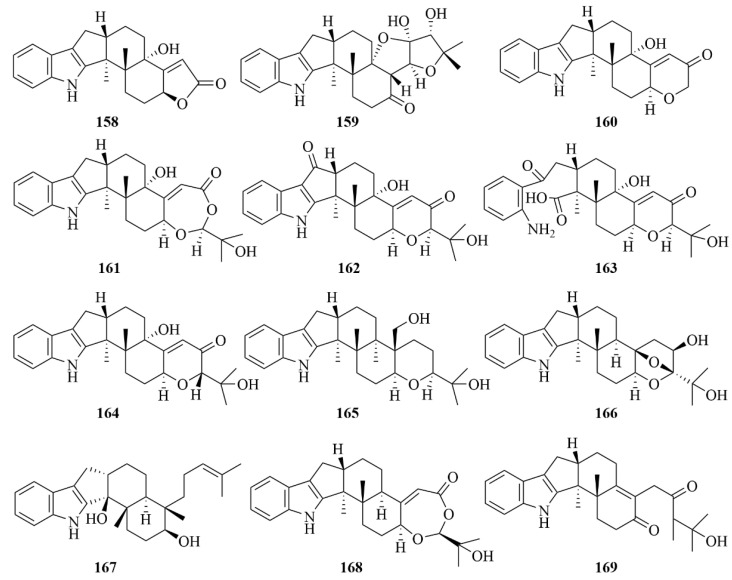
Chemical structures of diterpenoids from other-sourced fungi (**158**–**169**).

The study on the marine fungus *Penicillium* sp. KFD28, which was derived from the bivalve mollusk, led to the discovery of fifteen indole diterpenoids (**155**–**169**, Figure 19 and Figure 20) [67]. Among them, compound **161** was the first one featuring a rare pyridine-containing heptacyclic ring system, and compound **159** represented a unique 6/5/5/6/6/5/5 heptacyclic system [67]. To protein tyrosine phosphatase (PTP1B), Compounds **155**, **156**, **159**, **160**, **162**, **163**, and **168** exhibited great inhibitory activities with IC_50_ values of 1.7, 2.4, 14, 27, 23, 31.5, and 9.5 µM, respectively, compared to the positive control Na_3_VO_4_, with an IC_50_ value of 1.6 µM [67]. Additionally, compound **167** showed weak activity against HeLa cells with an IC_50_ value of 36.3 µM, compared to the positive control cisplatin, which was 8.6 µM [67].

#### 2.5.2. *Botryotinia* sp.

**Figure 21:**
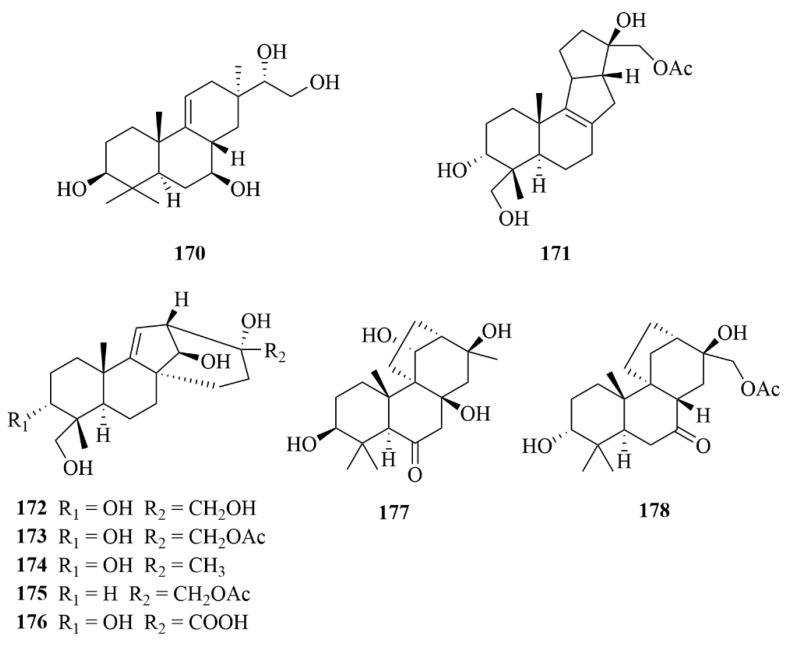
Chemical structures of diterpenoids from other-sourced fungi (**170**–**178**).

The team of Niu et al. obtained an undescribed rare pimarane diterpenoid, featuring a ∆^9(11)^ double bond, named botryopimarene A (**170**, Figure 21), and eight new diterpenoids, botryotins A–H (**171**–**178**, Figure 21), representing three new carbon skeletons with 6/6/5/5 (**171**), 6/6/5/6 (**172**–**176**), and 6/6/6/5 (**177** and **178**) tetracyclic scaffolds, from a deep-sea fungi *Botryotinia fuckeliana* MCCC in the western Pacific Ocean at a depth of −5572 m [68,69]. Botryopimarene A (**170**) was isolated from *Botryotinia fuckeliana* MCCC 3A00494, while compounds **171**–**178** lacked strain numbers in the original text. Botryotins A–H (**171**–**178**) played inactive against six HTCLs (HL-60, BEL-7402, BIU-87, PANC-1, HeLa-S3, and ECA109), each with IC_50_ values less than 20 µM, while compound **153** showed moderate antiallergic activity in RBL-2H3 cells, with an IC_50_ value of 0.2 mM, compared to the loratadine as p.c. (IC_50_ = 0.1 mM) [68,69].

**Figure 22:**
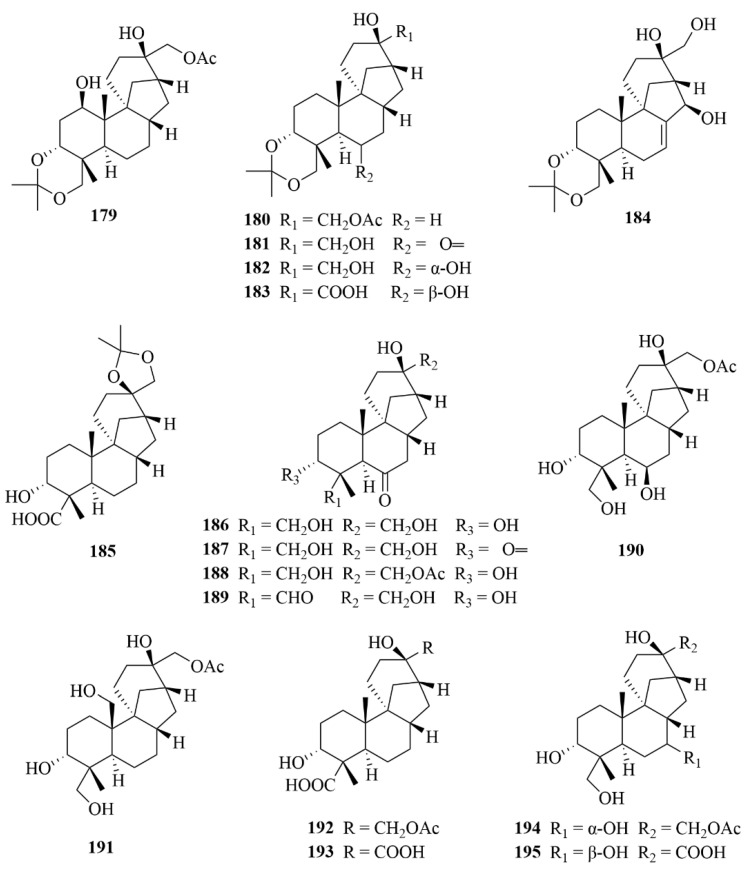
Chemical structures of diterpenoids from other-sourced fungi (**179**–**195**).

**Figure 23:**
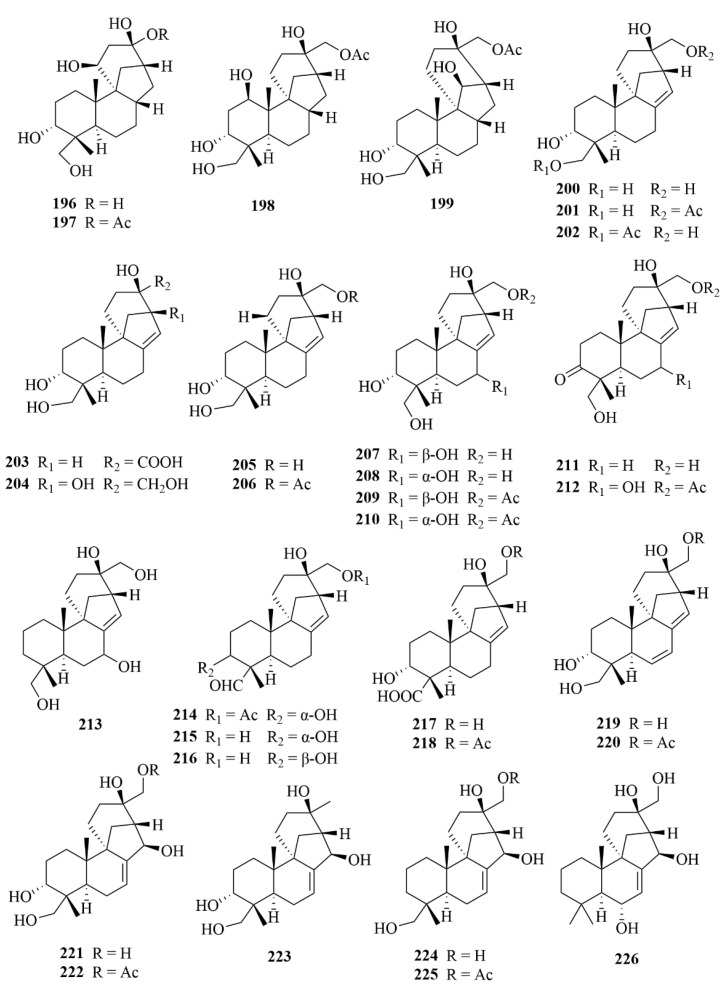
Chemical structures of diterpenoids from other-sourced fungi (**196**–**226**).

**Figure 24:**
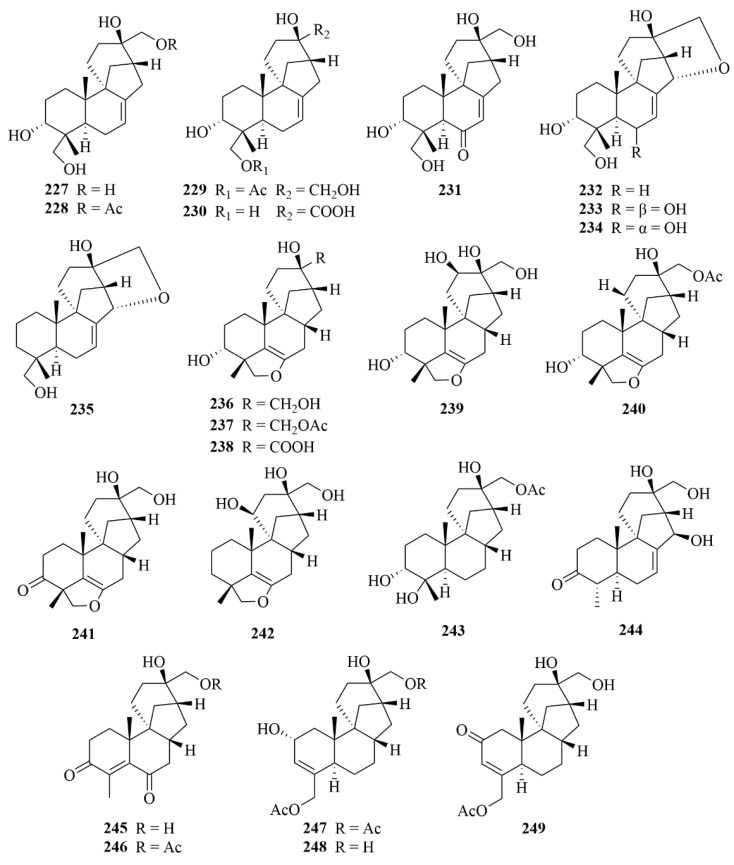
Chemical structures of diterpenoids from other-sourced fungi (**227**–**249**).

Further work conducted on the same strain led to the discovery of another 71 unprecedented diterpenoids, A1–A71 (**179**–**249**, Figure 22, Figure 23 and Figure 24), all belonging to aphidicolin congeners [70]. Among these diterpenoids, compounds **222**–**236** and **237**–**243** are new 6/6/5/6/5 pentacyclic aphidicolanes featuring tetrahydrofuran and dihydrofuran rings, respectively, and compounds 231–236 are rare noraphidicolins [70]. Compound **179** proved helpful as a potent cytotoxic lead compound due to its notable activities against T24 and HL-60 cells (IC_50_ = 2.5, 6.1 µM) [70].

#### 2.5.3. *Micromonospora* sp.

**Figure 25:**
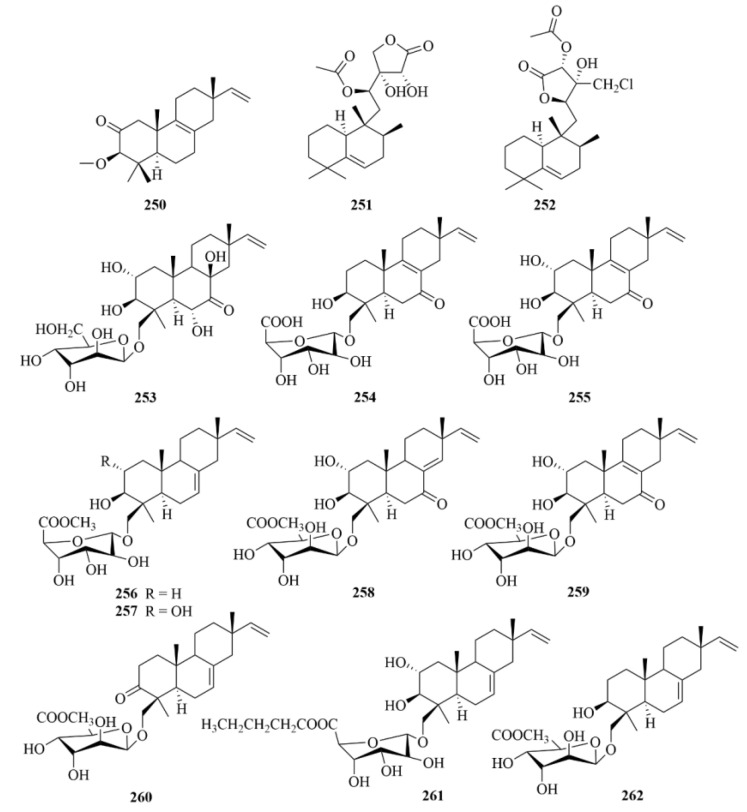
Chemical structures of diterpenoids from other-sourced fungi (**250**–**262**).

A novel ∆^8(9)^-pimarane diterpenoid, isopimara-2-one-3-ol-8,15-diene (**250**, Figure 25), was found by the team of Mullowney et al. from a marine-derived *Micromonospora* sp. in Vietnam’s east sea [71]. Combined with murine ovarian surface epithelial (MOSE) and murine oviductal epithelial (MOE), this compound showed no apparent cytotoxicity against two ovarian cancer cell lines (OVCAR4 and *Kuramochi*), with doxorubicin used as a positive control (LC_50_ = 0.078 µM) [71].

Two unreported halimane-type diterpenoids micromonohalimanes A (**251**, Figure 25) and B (**252**, Figure 25) were isolated from the fermentation of *Micromonospora* sp. WMMC-218 is a fungus derived from the marine ascidian *Symlegma brakenhielmi* [72]. Their unique secondary metabolite profiles were determined by LC-MS-based metabolomics [72]. Compound **252** inhibited the methicillin-resistant *Staphylococcus aureus* with an MIC value of 40 µg/mL, compared to the positive control, vancomycin, with an MIC value of 1 µg/mL [72].

#### 2.5.4. *Acremonium* sp.

On a wort agar medium treated with potassium bromide, ten new diterpenoids were identified, which were the secondary metabolites of the fungus *Acremonium striatisporum* KMM 4401 isolated from the marine holothurian *Eupentacta fraudatrix*, and they were named virescenosides Z9-Z18 (**253**–**262**, Figure 25) [73]. Compounds **253**, **254**, **256**, and **257** could observably decrease ROS production in macrophages under 10µM LPS stimulation [73]. Among them, compound **254** exhibited the most activity for inducing downregulation of ROS production by 45%. Moreover, at a concentration of 1 µM, virescenoside Z10 (**254**) and Z13 (**257**) decreased the NO production in LPS-stimulated macrophages [73].

#### 2.5.5. *Aspergillus* sp.

**Figure 26:**
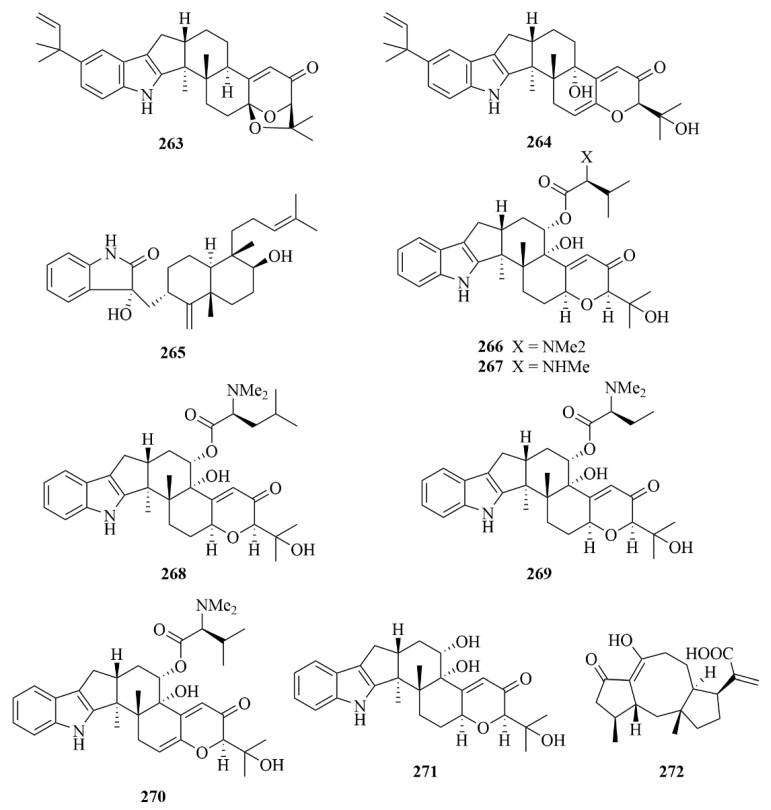
Chemical structures of diterpenoids from other-sourced fungi (**263**–**272**).

Two indole diterpenoids were isolated from the fungus *Aspergillus flavus* OUCMDZ-2205 derived from the *Penaeus vannamei* by Sun et al., identified as (2*R*, 4b*R*, 6a*S*, 12b*S*, 12c*S*, 14a*S*)-4b-Deoxyβ-aflatrem (**263**, Figure 26) and (2*R*, 4b*S*), 6a*S*, 12b*S*, 12c*R*)-9-Isopentenylpaxilline D (**264**, Figure 26) [74]. The biological assay revealed the cytotoxicity of compounds **263** and **264** against the A-549 cell cycle in the *S* phase with IC_50_ values of 10 µM [74]. In addition, compound **263** contributes to attenuating vascular complications of diabetes, due to its inhibition against the kinase PKC-β with an IC_50_ value of 15.6 µM [74]. A new indole diterpenoid, (3*R*, 9*S*, 12*R*, 13*S*, 17*S*, 18*S*)-2-carbonyl3hydroxylemeniveol (**265**, Figure 26), was isolated from a marine-derived fungus *Aspergillus versicolor* ZZ761 [75]. Compound **265** showed activity against *Escherichia coli* and *Candida albicans* with MIC values of 20.6 and 22.8 µM, respectively [75].

The isolation of six rare indole diterpenoids, noonindoles A–F (**266**–**271**, Figure 26), was revealed from the Australian marine-derived fungus *Aspergillus noonimiae* CMB-M0339 [76]. Their structures were determined by detailed spectroscopic and X-ray crystallographic analysis [76]. Only compound **266** showed moderate antifungal activity against the fungi *Candida albicans* [76].

From the culture extract of the marine-derived fungus *Aspergillus aculeatinus* WHUF0198, one unprecedented norditerpenoid was isolated by the team of Wu et al., namely aculeaterpene A (**272**, Figure 26) [77]. With the help of spectroscopic analysis, including 1D and 2D NMR and HR-ESI-MS experiments, the structure of compound **272** was expounded in detail, and its absolute configurations were displayed by comparing their experimental or calculated ECD spectra [77].

#### 2.5.6. *Epicoccum* sp.

**Figure 27:**
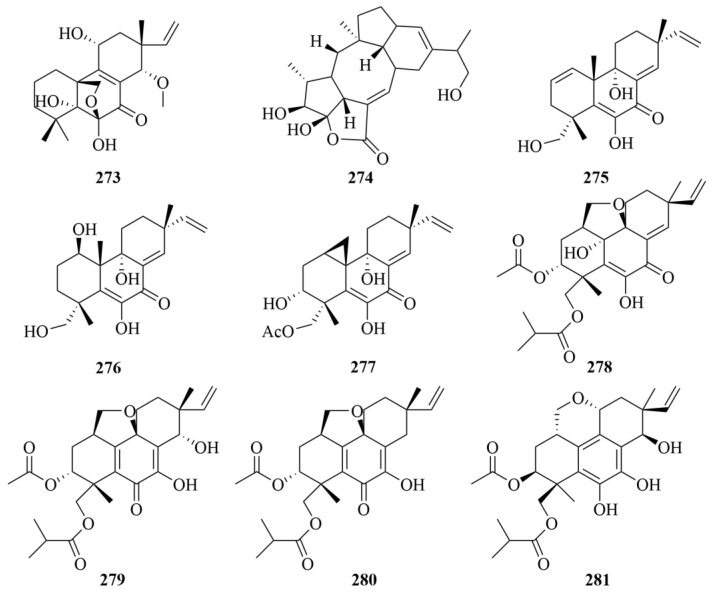
Chemical structures of diterpenoids from other-sourced fungi (**273**–**281**).

The *Apostichopus japonicus*-associated fungi *Epicoccum* sp. HS-1 produced a new isopimarane diterpenoid (**273**, Figure 27) [78]. It exhibited significant inhibitory effects on α-glucosidase, with an IC_50_ value of 4.6 µM, higher than the p.c. resveratrol, with IC_50_ = 31.2 µM [78].

#### 2.5.7. *Talaromyces* sp.

One new diterpenoid, roussoellol C (**274**, Figure 27), was obtained from the marine-derived fungus *Talaromyces purpurogenus* PP-414, which was isolated from a beach in Qinhuangdao, Hebei Province [79]. It exhibited cytotoxic activity against the MCF-7 cells with an IC_50_ of 6.5 µM [79].

#### 2.5.8. *Libertella* sp.

Marine-derived fungi *Libertella* sp. produced four diterpenoids, including libertellenone A (**44**), and libertellenones B-D (**275**–**277**, Figure 27) [80]. They showed weak activity against *Candida albicans* with MIC values less than 160 µg/mL [80]. Furthermore, significant cytotoxicity against HCT-116 (human adenocarcinoma cell line) was exerted by libertellenone D (**277**) with an IC_50_ value of 0.76 µM, while the other three compounds showed weaker activities (IC_50_ = 15, 15, and 53 µM) [80].

#### 2.5.9. *Eutypella* sp.

From the culture extract of *Eutypella* sp. D-1, the fungi isolated from the *Arctic region*, three undescribed pimarane diterpenoids, eutypellenoids A–C (**278**–**280**, Figure 27), and a known compound, eutypenoid C (**281**, Figure 27), were reported [81]. Among these diterpenoids, a range of biological activity was shown by compound **279** [81]. To Staphylococcus aureus and *Escherichia coli*, compound **279** displayed antibacterial activities with MIC values of 8 and 8 µg/mL, respectively [81]. To *Candida parapsilosis*, *Candida albicans*, *Candida glabrata*, and *Candida tropicalis*, compound **279** exhibited antifungal activities with MIC values of 8, 8, 16, and 32 µg/mL, respectively [81]. To the HCT-116 cell line, compound **279** showed moderate cytotoxic activity with an IC_50_ value of 3.7 µM [81].

## 3. Diterpenoids from Marine Invertebrates

Marine invertebrates are a special source of natural products, with great biological and pharmacological activities [82,83,84,85]. Among marine invertebrate sources, marine-derived diterpenes are mainly obtained from sponges and corals [86]. In all, we identified 162 diterpenes isolated from marine invertebrates, including 46 from sponges (28.40%), 106 from coral (65.43%), and 10 from sea hare (6.17%). Various biological activities exerted by the activities of these compounds are summarized below.

### 3.1. Sponge

**Figure 28:**
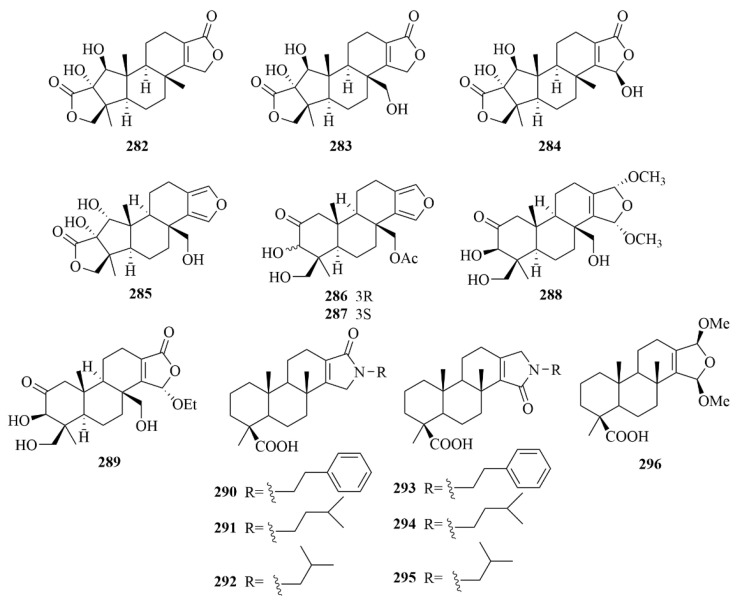
Chemical structures of diterpenoids from sponges (**282**–**296**).

Spongenolactones A-C (**282**–**284**, Figure 28), three novel 5,5,6,6,5-pentacyclic spongian diterpenes, were isolated from a Red Sea sponge *Spongia* sp. [87]. The biological activities of these compounds were evaluated, and all exhibited inhibitory effects against superoxide anion generation in fMLF/CB-stimulated human neutrophils [87]. Additionally, compared to spongenolactone B (**283**), spongenolactone A (**282**) was more active against the growth of Staphylococcus aureus [87].

The South China Sea sponge *Spongia officinalis* produced five new diterpenes (**285**–**289**, Figure 28), including sponalactone (**285**), which was a 5,5,6,6,5-pentacyclic diterpene, and two unprecedented spongian diterpenes, 17-O-acetylepispongiatriol (**286**) and 17-O-acetylspongiatriol (**287**), together with two novel spongian diterpene artifacts, namely 15α,16α-dimethoxy-15,16-dihydroepispongiatriol (**288**) and 15α-ethoxyepispongiatriol-16(15H)-one (**289**) [88]. To LPS-induced NO production in RAW264.7 macrophages, compounds **285**–**289** showed moderate inhibitory activities, with IC_50_ values of 12–32 µM [88].

Many experiments have been carried out on the Indonesian marine sponge *Spongia ceylonensis*. El-Desoky et al. isolated seven new spongian diterpenes, ceylonamides A-F (**290**–**295**, Figure 28) and 15α,16-dimethoxyspongi-13-en-19-oic acid (**296**, Figure 28) from the *Spongia ceylonensis* [89,90]. Their investigation on RANKL-induced osteoclastogenesis in RAW264 macrophages showed the significant inhibition of compound **290** with an IC_50_ value of 13 µM, and 18 µM of compound **291** [89,90].

**Figure 29:**
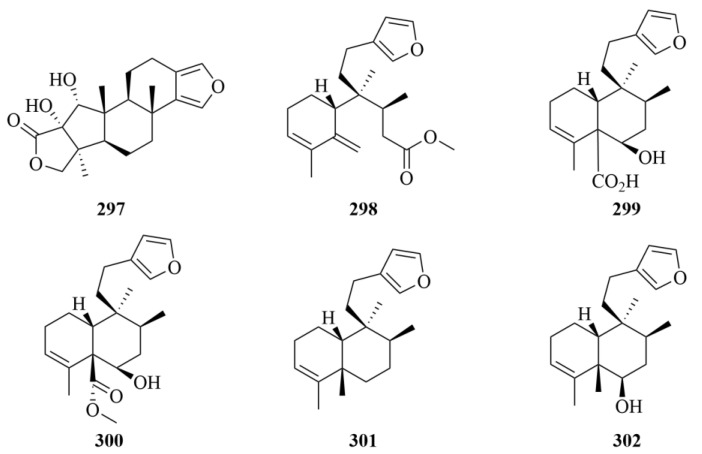
Chemical structures of diterpenoids from sponges (**297**–**302**).

The isolation of a rare A-ring contracted secospongian diterpene 17-dehydroxysponalactone (**297**, Figure 29) was revealed from a Red Sea sponge *Spongia* sp. [91]. Compound **297** exhibited noncytotoxicity but showed strong inhibitory activity against the superoxide anion generation and elastase release in the fMLF/CB-induced neutrophils, so it was supposed to be a promising candidate for further development of anti-inflammatory agents [91]. A study on the marine sponge *Raspailia bouryesnaultae* derived from South Brazil led to the discovery of one novel diterpene, raspadiene (**298**, Figure 29), and four diterpenes (**299**–**302**, Figure 29), which were elucidated as isomers of clerodane diterpenes previously obtained from plants, named kerlinic acid (**299**), kerlinic acid methyl ester (**300**), annonene (**301**), and 6-hydroxyannonene (**302**) [92]. In the evaluation of antiproliferative activities on human cancer cell line A549, the diterpenes with a hydroxyl group at C-6 showed moderate cytotoxic activity, with IC_50_ values lower than 25 µM [92]. Besides, compound **298** exhibited inhibitory activities against HSV-1 (KOS and 29R strains) replication by 83% and 74%, respectively, which proved that it may be a promising compound against herpes simplex virus type 1 (HSV-1, KOS, and 29R strains) [92].

**Figure 30:**
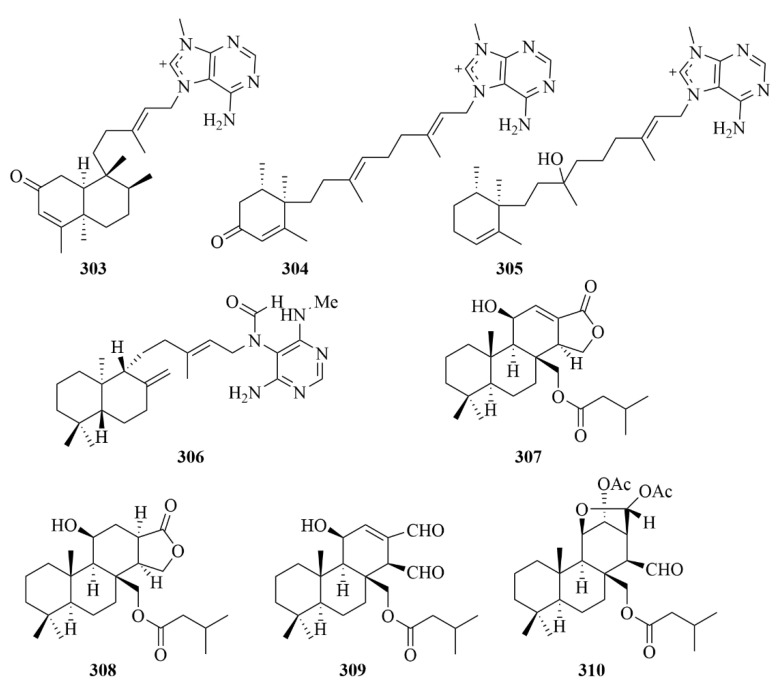
Chemical structures of diterpenoids from sponges (**303**–**310**).

The chemical investigation of marine sponge *Agelas nakamurai Hoshino* resulted in three novel N-methyladenine-containing diterpenes (**303**–**305**, Figure 30), namely 2oxoagelasines A (**303**) and F (**304**) and 10-hydro-9-hydroxyagelasine F (**305**) [93]. At 20 µg/disc, compound **304** exhibited inhibition against the growth of *Mycobacterium smegmatis* with inhibition zones of 10 mm [93]. In addition, compounds **303** and **304** showed significant activities against *M. smegmatis* [93].

A diterpene alkaloid, (-)-Agelamide D (**306**, Figure 30), was isolated from the marine sponge *Agelas* sp. [94]. It exerted active tumor growth inhibition by radiation without systemic toxicities and enhanced radiation-induced ATF4 expression and apoptotic cell death; these results proved that it could be a natural radiosensitizer in hepatocellular carcinoma models [94].

Four undescribed diterpenes (**307**–**310**, Figure 30) were obtained from the marine sponge *Dysidea* cf. *Arenaria*, collected from Irabu Island [95]. To NBT-T2 cells, four compounds all exhibited cytotoxicity with IC_50_ values of 3.1, 1.9, 8.4, and 3.1 µM, respectively [95].

**Figure 31:**
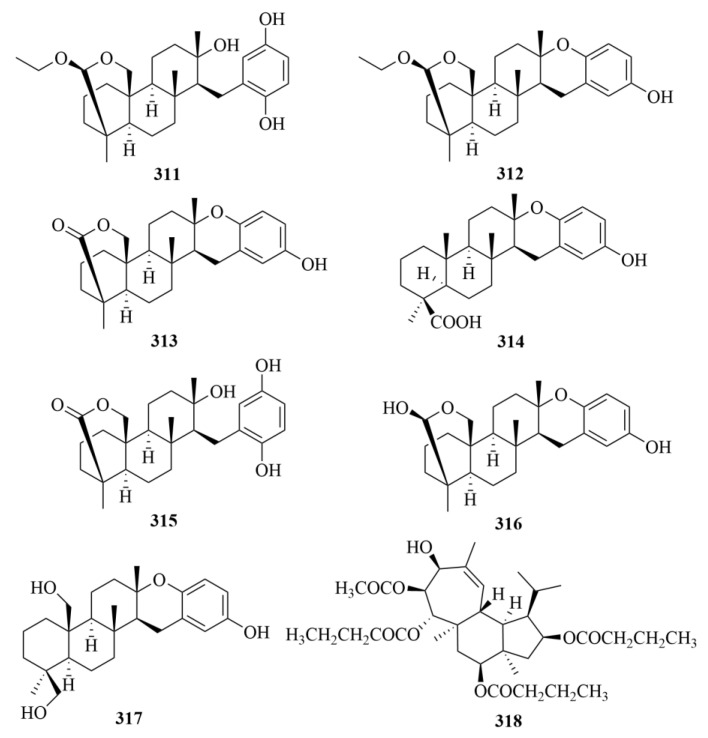
Chemical structures of diterpenoids from sponges (**311**–**318**).

The Okinawan marine sponge *Strongylophora strongilata* produced one novel meroditerpene, namely 26-O-ethylstrongylophorine-14 (**311**, Figure 31), together with six known strongylophorines (**312**–**317**, Figure 30): 26-O-methylstrongylophorine-16 (**312**) and strongylophorines-2 (**313**), -3 (**314**), -8 (**315**), -15 (**316**), and -17 (**317**) [96]. The inhibitory effect against protein tyrosine phosphatase 1B (PTP1B) was evaluated, and compounds **311**–**317** showed inhibition with IC_50_ values of 8.7, 8.5, >24.4, 9.0, 21.2, 11.9, and 14.8 lM, respectively, with oleanolic acid as the positive control (IC_50_ = 0.7 lM) [96]. It is worth mentioning that this is the first study to confirm the inhibition activities of meroditerpenes towards PTP1B [96].

The isolation of a highly oxygenated diterpene, named Gagunin D (GD) (**318**, Figure 31), was revealed from the marine sponge *Phorbas* sp. [97]. It has been proven that GD exhibits cytotoxicity against human leukemia cells [97]. The biological assay showed the significant activities of GD; it not only could suppress the expression of tyrosinase and increase the rate of tyrosinase degradation but also inhibited tyrosinase enzymatic activity [97]. With GD’s numerous effects on tyrosinase, which is the key to skin pigmentation controls, it is supposed to be a potential candidate for cosmetic formulations due to its multi-functional properties [97].

**Figure 32:**
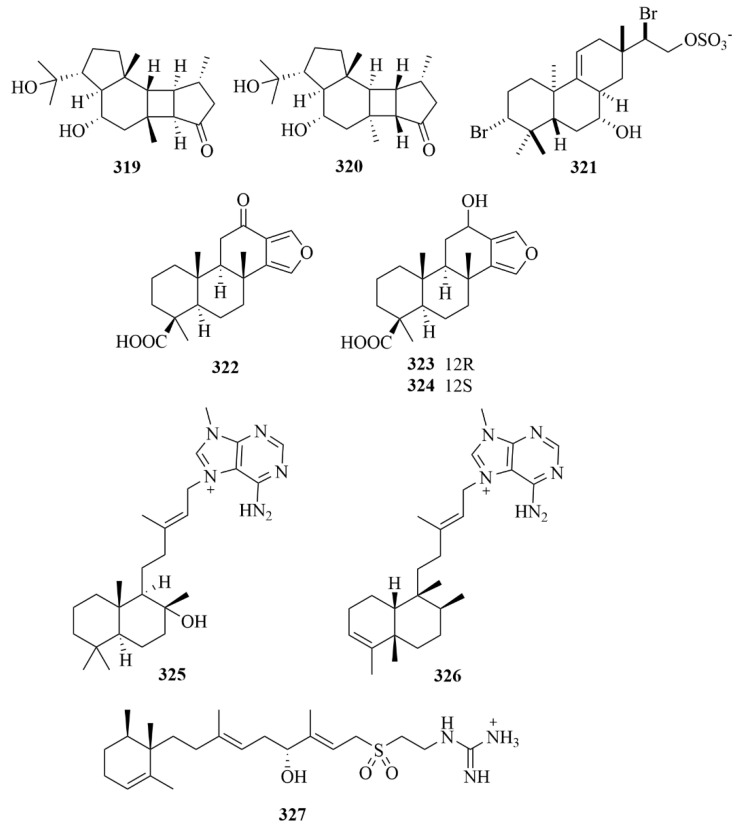
Chemical structures of diterpenoids from sponges (**319**–**327**).

The investigation of the marine sponge *Hippospongia lachne*, collected from the South China Sea, led to the isolation of two new anti-allergic diterpenoids, hipposponlachnins A (**319**, Figure 32) and B (**320**, Figure 32) [98]. Compounds **319** and **320** inhibited the release of biomarker β-hexosaminidase and the production of pro-inflammatory cytokine IL-4 and lipid mediator LTB4 in DNP-IgE stimulated RBL-2H3 cells [98].

Tedanol (**321**, Figure 32), which was a brominated and sulfated ent-pimarane diterpene, was obtained from the Caribbean sponge *Tedania ignis* [99]. It showed great anti-inflammatory activity at 1 mg/kg in a mouse model of inflammation in vivo [99]. In addition, in acute (4 h) and subchronic (48 h) phases, tedanol (**321**) could potently significantly reduce carrageenan-induced inflammation, which demonstrates that it has the potential to be a model of new anti-inflammatory molecules with low gastrointestinal toxicity [99].

Further study by the same team led to the isolation of three new spongian diterpenes, named ceylonins G–I (**322**–**324**, Figure 32), but they did not exhibit inhibition against USP7 [100].

The sponge *Agelas citrina*, which was derived from the coasts of the Yucatán Peninsula (Mexico), produced three undescribed diterpene alkaloids (**325**–**327**, Figure 32), namely (+)-8-epiagelasine T (**325**), (+)-10-epiagelasine B (**326**), and (+)-12-hydroxyagelasidine C (**327**) [101]. Among them, compound **326** exhibited the most activity against the Gram-positive pathogens (*Staphylococcus aureus*, *Streptococcus pneumoniae*, *Enterococcus faecalis*) with an MIC in the range of 1–8 µg/mL, while other compounds showed lower activities [101].

### 3.2. Coral

#### 3.2.1. *Sarcophyton* sp.

**Figure 33:**
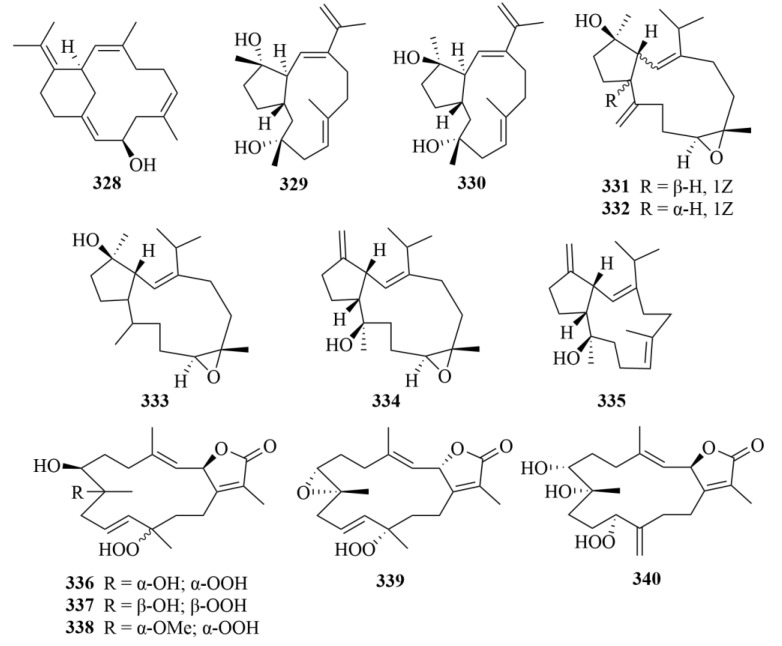
Chemical structures of diterpenoids from coral (**328**–**340**).

Mililatensols A–C (**328**–**330**, Figure 33), three novel diterpenes bearing unusual sarsolenane and capnosane skeletons, were isolated from the soft coral *Sarcophyton mililatensis* collected from the South China Sea [102]. By molecular docking, these compounds were proven to be potential inhibitors towards SARS-CoV-2 M^pro^ due to their great activities in the preliminary virtual screening of inhibitory potential against SARS-CoV-2 [102].

The isolation of five novel capnosane diterpenes, named sarboettgerins A–E (**331**–**335**, Figure 33), was revealed from the South China Sea soft coral *Sarcophyton boettgeri* [103]. Compound **335** exhibited significant anti-neuroinflammatory activity against LPS-induced NO release in BV-2 microglial cells in the biological assay, so it is a promising new type of neuroprotective agent in the future [103].

The first investigation of the red sea soft coral *Sarcophyton convolutum* afforded five highly oxygenated cembrenoids, sarcoconvolutum A–E (**336**–**340**, Figure 33) [104]. Their cytotoxicity was evaluated on lung adenocarcinoma, cervical cancer, and oral-cavity carcinoma (A549, HeLa, and HSC-2, respectively) [104]. Among them, compound **339** exhibited the most activity, showing cytotoxic activity against cell lines A549 and HSC-2 with IC_50_ values of 49.70 and 53.17 µM, respectively [104].

**Figure 34:**
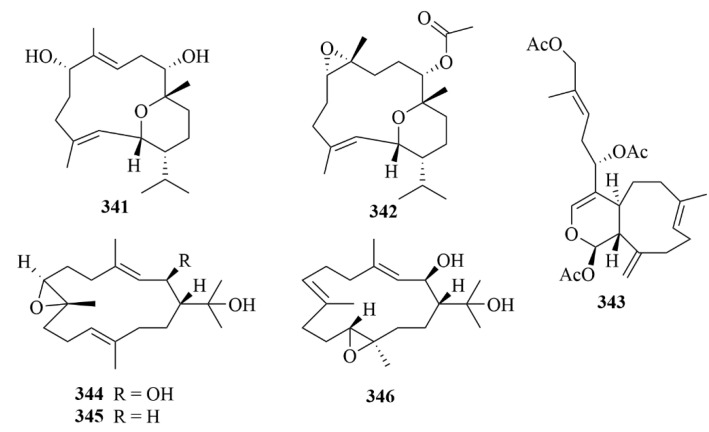
Chemical structures of diterpenoids from coral (**341**–**346**).

Two novel pyranosyl cembranoid diterpenes, 9-hydroxy-7,8dehydro-sarcotrocheliol (**341**, Figure 34) and 8,9-expoy-sarcotrocheliol acetate (**342**, Figure 34), were obtained from the soft coral *Sarcophyton trocheliophorum* [105].

Waixenicin A (**343**, Figure 34), a new xenicane diterpenoid, was isolated from the Hawaiian soft coral *Sarcothelia edmondsoni* [106]. The bioactive test proved that waixenicin A reduced hypoxic-ischemic brain injury and preserved long-term behavioral outcomes in mouse neonates [106]. As the most potent and specific inhibitor available for TRPM7, which is an emerging drug target for CNS diseases and disorders, waixenicin A is thought to be a viable and potential drug lead for these disorders [106].

#### 3.2.2. *Nephthea* sp.

Hsiao et al. isolated two unprecedented 15-hydroxycembranoid diterpenes (**344**–**346**, Figure 34), namely 2β-hydroxy-7β,8α-epoxynephthenol (**344**) and 2β-hydroxy-11α,12β-epoxynephthenol (**3345**), and a novel natural cembrane-type epoxynephthenol (**346**) from extracts of the octocoral *Nephthea columnaris* [107].

#### 3.2.3. *Sinularia* sp.

**Figure 35:**
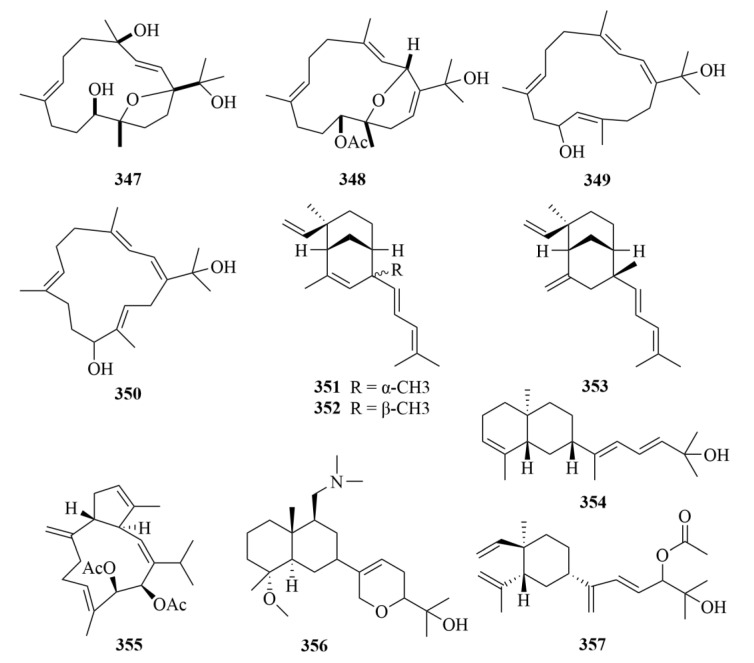
Chemical structures of diterpenoids from coral (**347**–**357**).

Numerosols A–D (**347**–**350**, Figure 35), four novel cembrane-based diterpenes, were isolated from the Taiwanese soft coral *Sinularia numerosa* [108]. No significant activity was shown in the bioassay [108].

Xisha soft coral *Sinularia polydactyla* afforded three uncommon novel diterpenes with unprecedented carbon skeletons (**351**–**355**, Figure 35), together with a new prenyleudesmane type diterpene, sinupol (**354**, Figure 35), and a new capnosane type diterpenoid, sinulacetate (**355**, Figure 35) [109]. Through extensive spectroscopic analysis, the comparison of their NMR data with those of related compounds, and time-dependent density functional theory electronic circular dichroism (TDDFT ECD) calculations, the structure of compounds **354** and **355** was demonstrated [109]. They showed notable activity against protein tyrosine phosphatase 1B (PTP1B), and since PTP1B is a promising drug target for type II diabetes and obesity, compounds **354** and **355** may contribute to the treatment of type II diabetes and obesity [109].

Two new compounds, (4R*, 5R*, 9S*, 10R*, 11*Z*)4-methoxy-9-((dimethylamino)-methyl)-12,15-epoxy-11(13)-en-decahydronaphthalen-16-ol (**356**, Figure 35) and the lobane (1R*, 2R*, 4S*, 15*E*)-loba-8,10,13(14),15(16)-tetraen-17,18-diol-17-acetate (**357**, Figure 35), were produced by the Australian soft coral *Sinularia* sp. [110]. The two compounds inhibited the growth of three human tumor cell lines (SF-268, MCF-7, and H460), and compound **356** showed a lower activity with a GI_50_ value of 70–175 µM [110].

#### 3.2.4. *Lobophytum* sp.

**Figure 36:**
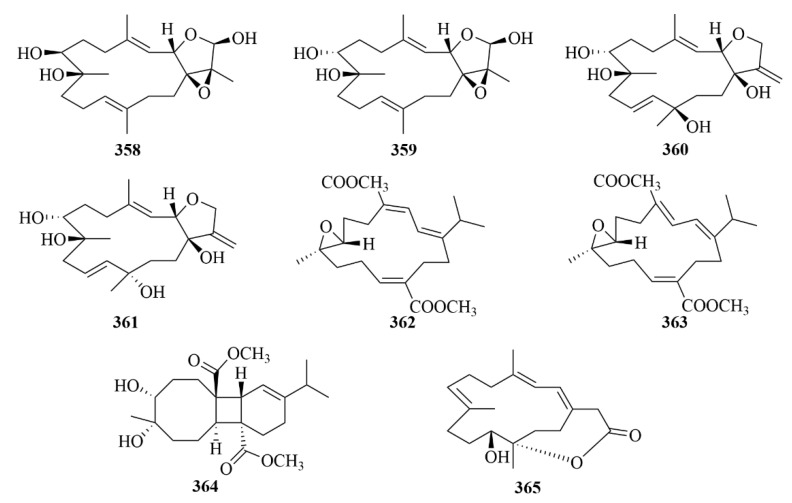
Chemical structures of diterpenoids from coral (**358**–**365**).

Four undescribed cembrane-type diterpenes, namely lobocrasols A–D (**358**–**361**, Figure 36), were obtained from the methanol extract of the soft coral *Lobophytum crassum* [111]. Compounds **358** and **359** showed potent inhibition against TNFa-induced NF-jB transcriptional activity in HepG2 cells in a dose-dependent manner (IC_50_ = 6.30 ± 0.42, 6.63 ± 0.11 lM) [111]. Additionally, these compounds could decrease the gene expression levels in HepG2 cells in cyclooxygenase-2 (COX-2) and inducible nitric oxide synthase (iNOS) to inhibit transcription [111].

**Figure 37:**
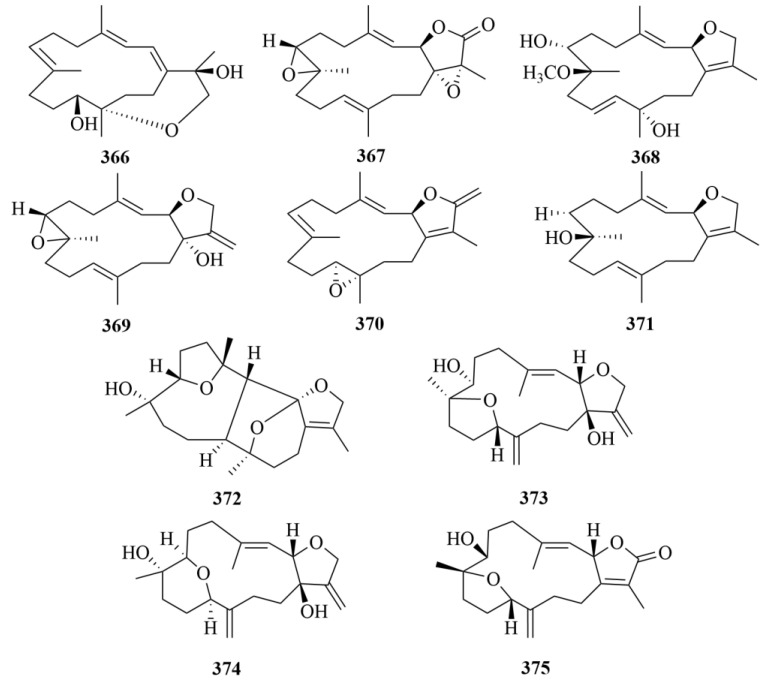
Chemical structures of diterpenoids from coral (**366**–**375**).

Further study on the soft coral *Lobophytum crassum* yielded ten new cembranoid diterpenes (**362**–**371**, Figure 36 and Figure 37), locrassumins A–G (**362**–**368**), (−)-laevigatol B (**369**), (−)-isosarcophine (**370**), and (−)-7*R*, 8*S*-dihydroxydeepoxysarcophytoxide (**371**) [112]. The bioactive test demonstrated the moderate inhibition of compounds **362** and **368**, against lipopolysaccharide (LPS)-induced nitric oxide (NO) production with IC_50_ values of 8–24 µM [112].

Four novel cembranoid diterpenes, namely crassumols D–G (**372**–**375**, Figure 37), were obtained from the methanol extract of the Vietnamese soft coral *Lobophytum crassum* [113]. Their structures were revealed by spectroscopic methods [113].

#### 3.2.5. *Junceella* sp.

**Figure 38:**
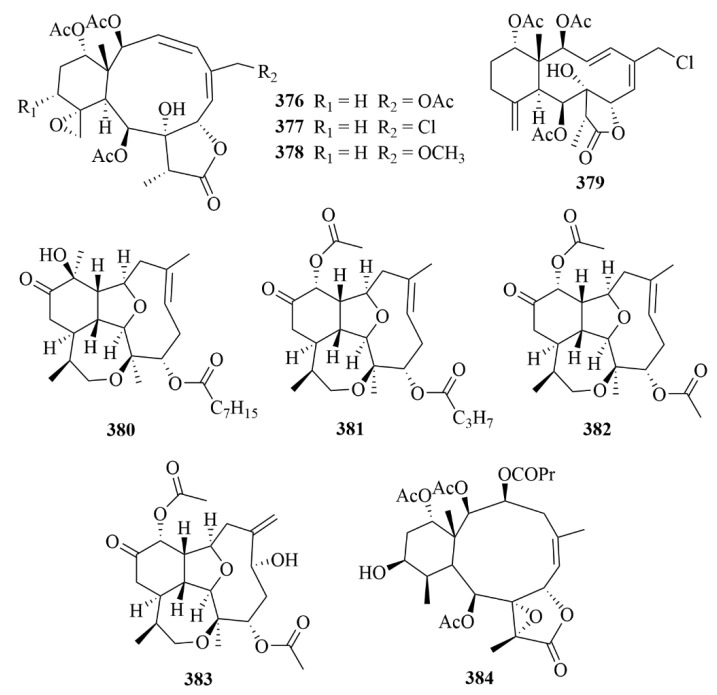
Chemical structures of diterpenoids from coral (**376**–**384**).

The South China Sea Gorgonian Coral, *Junceella gemmacea*, produced four new briarane diterpenoids, named junceellolides M–P (**376**–**379**, Figure 38) [114]. In an in vitro biological investigation on A549, MG63, and SMMC-7721 cell lines, however, none of these compounds exhibited growth inhibitory activity [114].

#### 3.2.6. *Briareum* sp.

The study of the methanolic extract of *Briareum asbestinum*, an octocoral collected in Bocas del Toro, on the Caribbean side of Panama, resulted in the discovery of three new eunicellin-type diterpenes (**380**–**382**, Figure 38), named briarellin T (**380**), asbestinin 27 (**381**), and asbestinin 28 (**382**), together with a known compound, asbestinin 17 (**383**, Figure 38) [115]. Compounds **380**–**383** were obtained by reversed-phase solid-phase extraction (SPE) and HPLC purification. Their potential for anti-inflammatory activity was well proven through the downregulation of the pro-inflammatory cytokines TNF-α, IL-6, IL-1β, and IL-8 as well as the reduction of COX-2 expression in LPS-induced THP-1 macrophages [115].

Excavatolide B (**384**, Figure 38), a marine-derived diterpenoid with great pharmacological activity, was isolated from Formosan gorgonian *Briareum excavatum* [116]. In the evaluation of the mRNA expression of the proinflammatory mediators, inducible nitric oxide synthase (iNOS) and cyclooxygenase-2 (COX-2), in lipopolysaccharide (LPS)-challenged murine macrophages (RAW 264.7), excavatolide B exhibited significant inhibition [116]. It could potentially deaden carrageenan-induced nociceptive behaviors, mechanical allodynia, thermal hyperalgesia, weight-bearing deficits, and paw edema [116]. In addition, excavatolide B (**384**) also showed inhibitory activity against iNOS and the infiltration of immune cells in carrageenan-induced inflammatory paw tissue [116].

#### 3.2.7. *Anthelia* sp.

**Figure 39:**
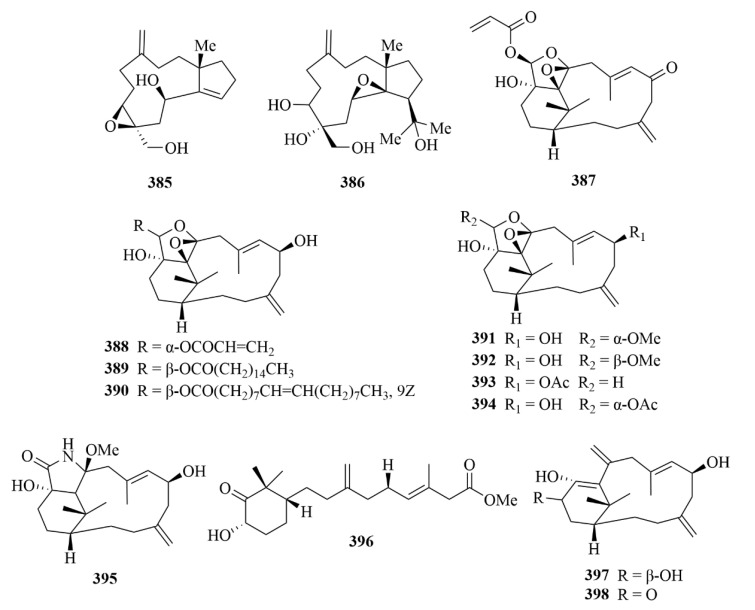
Chemical structures of diterpenoids from coral (**385**–**398**).

Two new marine-derived diterpenes, including a trinor-dolabellane diterpenoid, sangiangol A (**385**, Figure 39), and one dolabellane diterpenoid, sangiangol B (**386**, Figure 39), were identified from an Indonesian marine soft coral, *Anthelia* sp. [117]. Their structures were determined by spectral and molecular modelling studies [117]. In addition, the bioassay showed the moderate cytotoxicity of two compounds against an NBT-T2 cell line (0.5–10 µg/mL) [117].

#### 3.2.8. *Cespitularia* sp.

Twelve new verticillane-type diterpenes and norditerpenes were isolated from the soft coral *Cespitularia* sp. [118]. Among them, there are eight novel verticillane-type diterpenes, cespitulins H–O (**387**–**394**, Figure 39), one new cyclic verticillane-type diterpenoidal, amide cespitulactam L (**395**, Figure 39), and three new verticillane-type norditerpenes, cespitulins P–R (**396**–**398**, Figure 39) [118]. Their structure elucidation was achieved by extensive spectroscopic analyses, including 2D NMR experiments [118]. The investigation demonstrated the similarity between the structural framework of verticillane-type derivatives and the tricyclic taxane skeleton, and the soft coral genus *Cespitularia* was the only source of the structural framework of verticillane-type derivatives [118]. Compounds **387**–**389** showed great anti-inflammatory activities, especially **387** and **388**; not only could they potently inhibit the production of TNF-α and NO, but they also displayed potent suppression of the expression of iNOS and the COX-2 gene [118].

#### 3.2.9. *Klyxum* sp.

**Figure 40:**
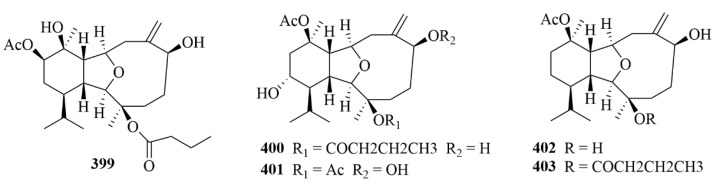
Chemical structures of diterpenoids from coral (**399**–**403**).

The soft coral *Klyxum simplex* produced four novel eunicellin-based diterpenes, simplexins P–S (**399**–**402**, Figure 40), and the known simplexin A (**403**, Figure 40) [119]. The structures of four new compounds were clarified by extensive spectroscopic analysis, including 1D and 2D NMR experiments [119]. Compounds **399** and **401**–**403** showed cytotoxicity against a limited panel of cancer cell lines, especially **401** [119].

#### 3.2.10. Vietnamese Soft Corals

**Figure 41:**
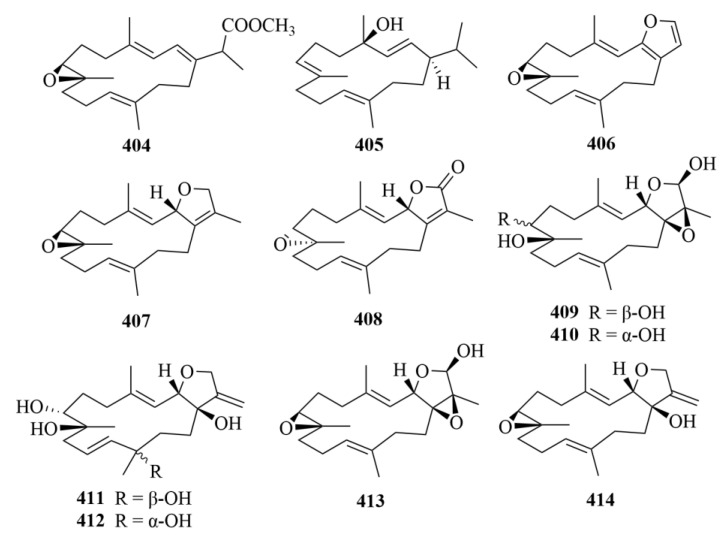
Chemical structures of diterpenoids from coral (**404**–**414**).

**Figure 42:**
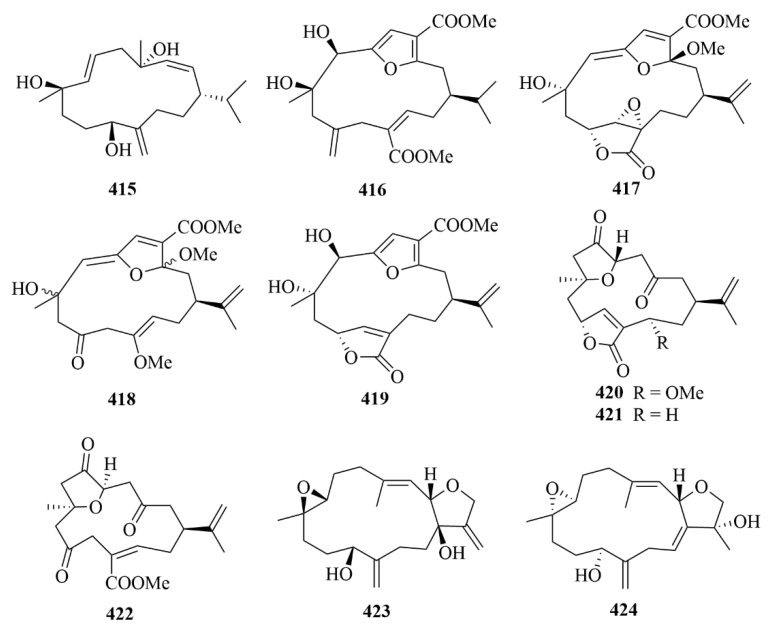
Chemical structures of diterpenoids from coral (**415**–**424**).

**Figure 43:**
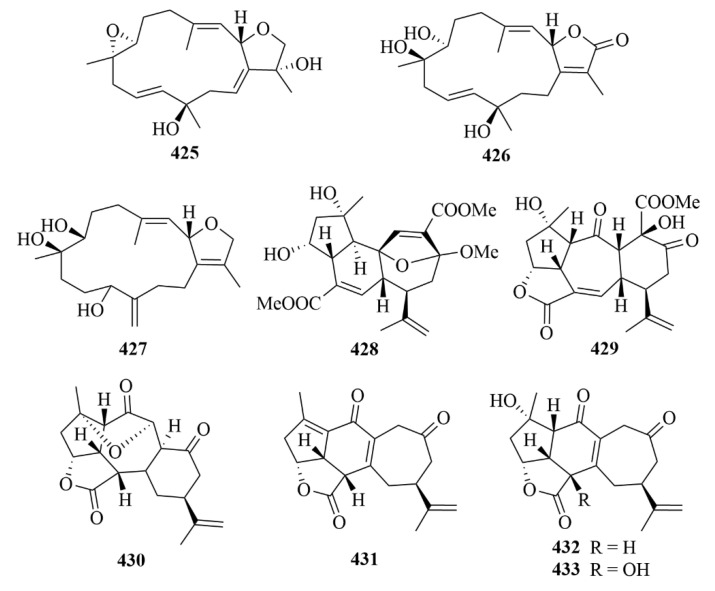
Chemical structures of diterpenoids from coral (**425**–**433**).

Thirty-four cembrane-type diterpenes (**404**–**433**, Figure 41, Figure 42 and Figure 43) were isolated from Vietnamese soft corals. Only 12 of them had anti-protozoal activities, including 7*S*, 8*S*-epoxy-1,3,11-cembratriene-16-oic methyl ester (**404**), (1*R*, 4*R*, 2*E*, 7*E*, 11*E*)-cembra-2,7,11-trien-4-ol (**405**), lobocrasols A-C (**409**–**411**), laevigatol A (**413**), crassumols D-G (**378**–**381**), (1*S*, 2*E*, 4*S*, 6*E*, 8*S*, 11*S*)-2,6,12(20)-cembrantriene-4,8,11-triol (**415**), sinumaximol A (**416**), sinumaximol C (**417**), and 13-Epi-scabrolide C (**420**) [120]. Among them, compounds **404**, **405**, **409**, **411**, **415**, **417**, and **420** exhibited activities against bloodstream forms of *T. brucei* [120]. Meanwhile, lobocrasol A (**409**) and lobocrasol C (**411**) showed potent and selective activity against *L. donovani* [120]. It is worth mentioning that laevigatol A (**413**) was the only compound that showed moderate antiplasmodial activity with an IC_50_ of no more than 5.0 µM [120]. However, none of these compounds showed obvious cytotoxicity [120].

### 3.3. Sea Hare

**Figure 44:**
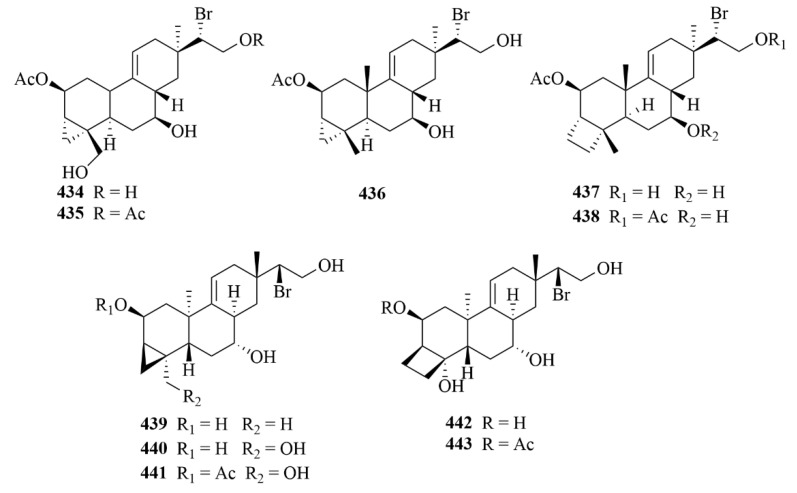
Chemical structures of diterpenoids from sea hare (**434**–**443**).

From the sea hare *Aplysia. Dactylomela*, five brominated diterpenes (**434**–**438**, Figure 44) were discovered, including parguerol (**434**), parguerol 16-acetate (**435**), deoxyparguerol (**436**), isoparguerol (**437**), and isoparguerol 16-acetate (**438**) [121]. Compounds **434**–**438** showed potent inhibition against P388 murine leukemia cells (IC_50_ = 8.3, 8.6, 0.86, 10.1, 1.0 µM) [121].

The chemical investigation of the sea hare *Aplysia pulmonica* from the South China Sea contributed to the isolation of five brominated ent-pimarane diterpenoids, namely compounds **439** and **440**–**443** [122]. To *Artemia salina*, compounds **442** and **443** showed low toxicity at a concentration of 0.5 µM [122]. In addition, with ciprooxacin used as the positive control, these compounds were evaluated for antibacterial activity towards *E. coli*, *S. aureus*, *S. albus*, *B. cereus*, *V. parahemolyticus*, *V. anguillarum*, and *P. putida*, but no significant results were reported [122].

## 4. Diterpenoids from Marine Plants

Plants produce numerous natural products and are another important source of diterpenes [123]. Plant diterpenes are important metabolites with various industrial and biological values [124,125,126]. Marine algae and mangroves are important components of marine ecosystems and two major sources of marine diterpenes [127]. In this part, a total of 56 diterpenoids of marine plant origin are summarized; all were derived from algae.

### 4.1. Algae

**Figure 45:**
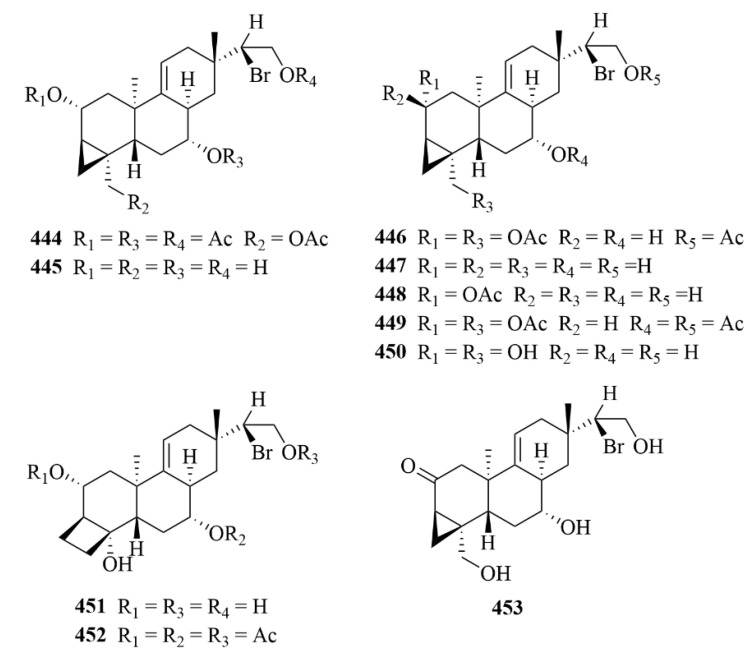
Chemical structures of diterpenoids from algae (**444**–**453**).

Two ent-pimarane diternpenes, 15-bromo-2,7,16,19-tetraacetoxy-9(11)-parguerene (**444**, Figure 45) and 15-bromo-2,7,16-tetraacetoxy-9(11)-parguerene (**445**, Figure 45), were found from *Laurencia obtusa (Hudson) Lamouroux*, the marine red algae from the Teuri island of Hokkaido [128]. This is the first time that an ent-pimarne diterpenoid was isolated from a marine organism [128]. Compound **444** exhibited cytotoxicity, but **445** did not [128]. Moreover, eight brominated diterpenoids, compounds **446**–**453 (**Figure 45**)**, were isolated from the same plant [128]. In the biological test of cytotoxic activity, compounds **446**, **450**, **451**, and **453** showed cytotoxic activity against HeLa, with IC_50_ values of 5.7, 0.68, 10.8, and 11.6 µM, respectively, and against P388 cell lines, with IC_50_ values of 6.5, 2.5, 14.6, and 18.3 µM, respectively, while no significant activity was exhibited by the other compounds [128].

**Figure 46:**
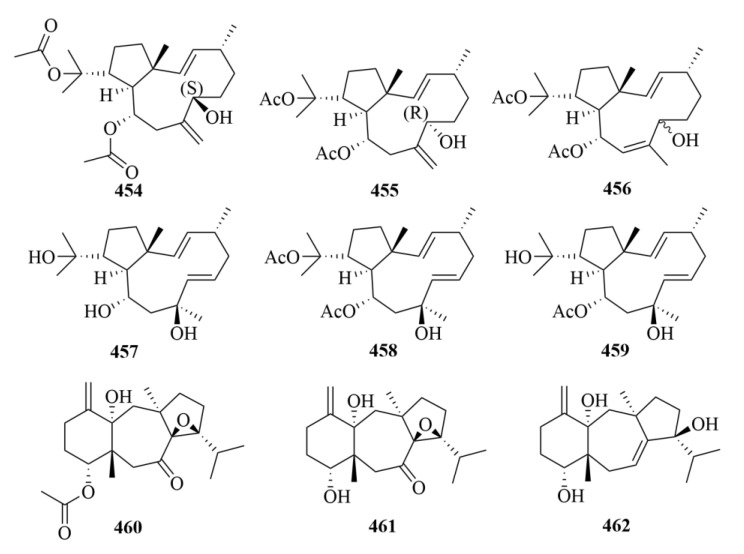
Chemical structures of diterpenoids from algae (**454**–**462**).

Three new dolabellane diterpenes, dolabelladienols A–C (**454**–**456**, Figure 46), together with three known dolabellane diterpenes (**457**–**459**, Figure 46) were isolated from the marine brown algae *Dictyota pfaffii*, collected from *Atol das Rocas*, in Northeast Brazil [129]. The structures of three new compounds were identified as (1R*, 2*E*, 4R*, 7*S*, 10S*, 11S*, 12R*)10,18-diacetoxy-7-hydroxy-2,8(17)-dolabelladiene (**454**), (1R*, 2*E*, 4R*, 7R*, 10S*, 11S*, 12R*)10, 18-diacetoxy-7-hydroxy-2, 8(17)-dolabelladiene (**455**), and (1R*, 2*E*, 4R*, 8*E*, 10S*, 11*S*, 12R*)10, 18-diacetoxy-7-hydroxy-2, 8-dolabelladiene (**456**) [129]. Compounds **454** and **455** exhibited more active anti-HIV-1 activities than compound **457**, with IC_50_ values of 2.9 and 4.1 µM, while their cytotoxic activity against MT-2 lymphocyte tumor cells was lower [129]. The results demonstrate that these compounds could be promising anti-HIV-1 agents [129].

The study on the Jamaican macroalga *Canistrocarpus cervicornis* resulted in the discovery of two new dolastane diterpenes, 4*R*-acetoxy-8*S*,9*S*-epoxy-14*S*-hydroxy-7-oxodolastane (**460**, Figure 46) and 4*R*-hydroxy-8*S*,9*S*-epoxy-14*S*-hydroxy-7-oxodolastane (**461**, Figure 46), together with the known dolastane (4*R*, 9*S*, 14*S*)-4,9,14-trihydroxydolast-1(15), 7-diene (**462**, Figure 45) [130]. Compounds **460**–**462** exhibited moderate and concentration-dependent cytotoxic activity against human tumor cell lines PC3 and HT29 [130].

**Figure 47:**
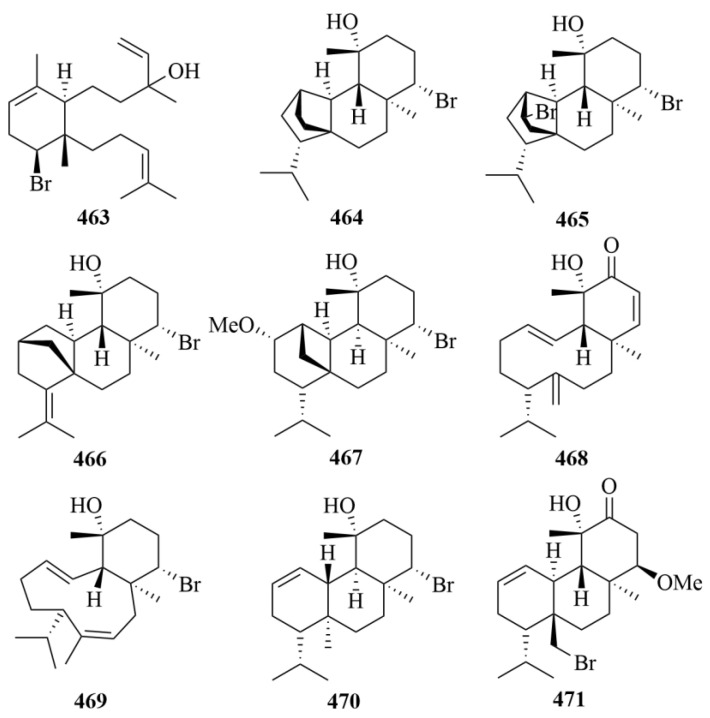
Chemical structures of diterpenoids from algae (**463**–**471**).

One unprecedented brominated diterpene of the dactylomelane family was isolated from the red algae *Sphaerococcus coronopifolius*, namely sphaerodactylomelol (**463**, Figure 47) [131]. Tests were carried out on the activity of compound **463**, and it exhibited antimicrobial activity against S. aureus with an IC_50_ value of 96.3 µM [131]. To HepG-2 cells, compound **463** showed cytotoxicity with an IC_50_ value of 720 µM and induced inhibition of cell proliferation with an IC_50_ value of 280 µM [131].

From the extracts of the red algae *Sphaerococcus coronopifolius*, which was collected from the coastline of the Ionian Sea in Greece, eight novel diterpenes (**464**–**471**, Figure 47) bearing five different carbocycles were discovered [132]. In vitro growth inhibitory activity of compounds **464**–**471** was evaluated on one murine cancer cell line (B16F10) and five human cancer cell lines (A549, Hs683, MCF7, U373); compounds **468** and **471** showed antitumor activity with IC_50_ values 15 and 16 µM, respectively, and doxorubicin was used as a positive control [132].

**Figure 48:**
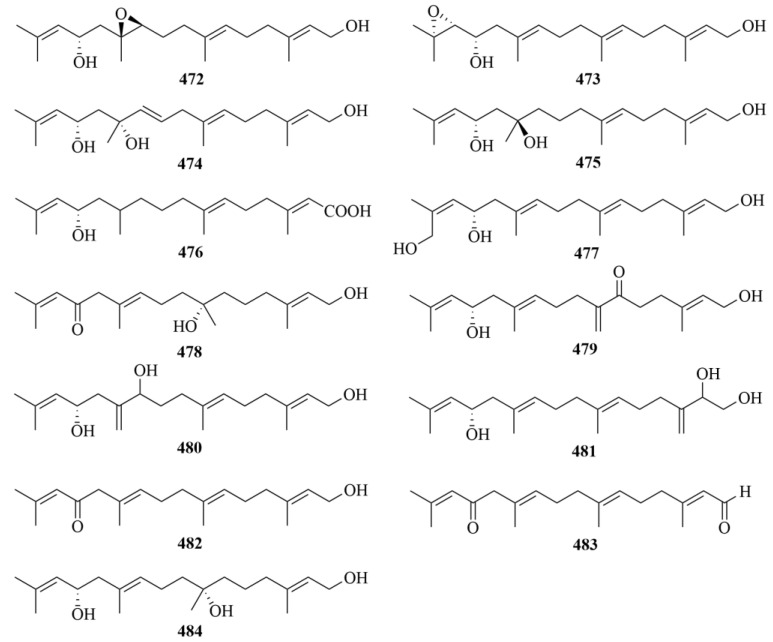
Chemical structures of diterpenoids from algae (**472**–**484**).

Four new acyclic diterpenes (**472**–**475**, Figure 48) were isolated from the brown algae *Bifurcaria bifurcate*, and their structures were revealed by means of 1D and 2D NMR, HRMS, and FT-IR spectroscopy [133]. At 100 µg/mL test concentration, compound **473** showed inhibition against the growth of cancer cells (78.8%), while compounds **472**, **473,** and **475** did not exhibit that activity [133].

Further investigation on the brown seaweed *Bifurcaria bifurcate* led to the isolation of six new acyclic diterpenes (**476**–**481**, Figure 48), eleganolone (**482**), and eleganonal (**483**), as well as bifurcatriol (**484**) [134,135,136]. The bioassay revealed that compounds **476**–**481** exhibited moderate inhibition against the growth of the MDA-MB-231 cell line, with IC_50_ values ranging from 11.6 to 32.0 µg/mL [134]. Eleganolone (**482**) and eleganonal (**483**) exerted antioxidant potential by FRAP and ORAC assays, which demonstrated that they may be potential candidates for further neuroprotection assays of PD [135]. For the malaria parasite *P. falciparum*, bifurcatriol (**484**) showed the highest activity (IC_50_ = 0.65 µg/mL) with low cytotoxicity (IC_50_ = 56.6 µg/mL) [136].

**Figure 49:**
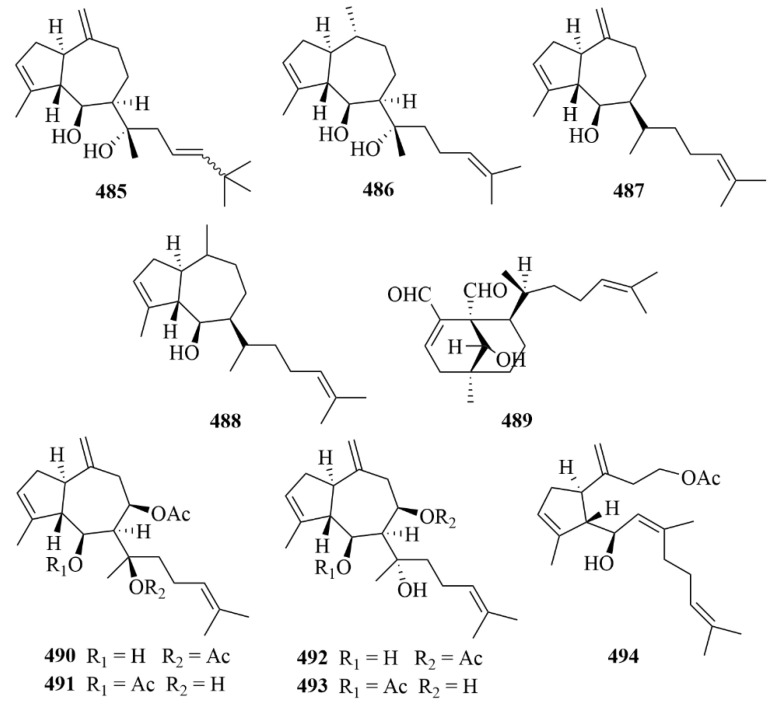
Chemical structures of diterpenoids from algae (**485**–**494**).

The brown algae of the genus *Dictyota* produced five new diterpenes (**485**–**489**, Figure 49), including pachydictyols B (**485a**/**485b**) and C (**486**) isolated from *Dictyota dichotoma* and pachydictyol A (**487**), isopachydictyol A (**488**), and dichotomanol (**489**), which were obtained from *Dictyota menstrualis* [137,138]. Weak antimicrobial properties were exhibited by pachydictyol B (**485a**) [138]. The extract of crude algal exerted notable activities against the breast carcinoma tumor cell line MCF7 with an IC_50_ value of 0.6 µg/mL^−1^, whereas those compounds isolated from it only showed very weak activity [138]. Compounds **487**–**489** were useful in the studies of more active antithrombotic prototypes [137].

Further study on the *Dictyota* brown algae led to the isolation of another five novel diterpenes, including four new hydroazulenes (**490**–**494**, Figure 49), (8*R*, 11*R*)-8,11-diacetoxypachydictyol A (**490**), (8R*, 11R*)-6-O-acetyl-8-acetoxy-11-hydroxypachydictyol A (**491**), (8R*, 11S*)-8-acetoxy-11-hydroxypachydictyol A (**492**), and (8R*, 11S*)-6-O-acetyl-8,11-dihydroxypachydictyol A (**493**), and a secohydroazulene derivative, named 7Z-7,8-seco-7,11-didehydro-8- acetoxypachydictyol A (**494**) [139]. Extensive spectral analysis and comparison with reported data elucidated the structure of the compounds [139]. Additionally, potent antioxidant activities against H_2_O_2_-induced oxidative damage in neuron-like PC12 cells at a low concentration of 2 µM were significantly exhibited by all compounds [139].

**Figure 50:**
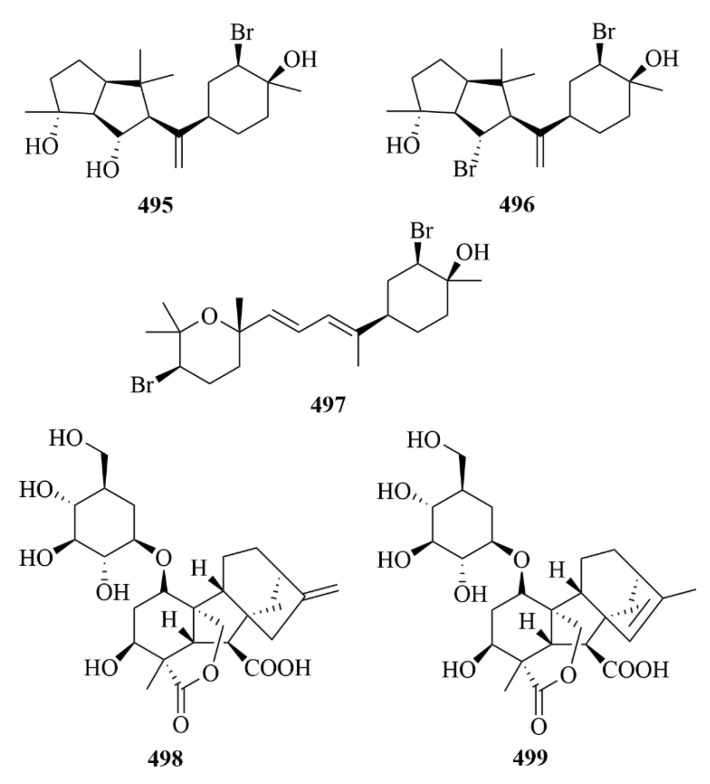
Chemical structures of diterpenoids from algae (**495**–**499**).

Three diterpenes (**495**–**497**, Figure 50), neorogioltriol (**495**), neorogioldiol (**496**), and O^11^,15-cyclo-14-bromo-14,15-dihydrorogiol-3,11-diol (**497**), were isolated from the red algae *Laurencia* [140]. All three compounds could suppress macrophage activation and promote an M2-like anti-inflammatory phenotype. Thus, they have proven to be useful in the development of anti-inflammatory agents targeting macrophage polarization mechanisms [140].

Enhoidin A (**498**, Figure 50) and Enhoidin B (**499**, Figure 50), two undescribed diterpenes bearing a rare gibberellane skeleton, were obtained from the stems and leaves of tropical seagrass Enhalus acoroides in the South China Sea [141]. The structures of the two compounds were established by spectroscopic analysis, including 1D and 2D NMR techniques and HR-ESI-MS [141]. To four human cancer cell lines (MCF-7, HCT-116, HepG-2, and HeLa), all compounds exhibited moderate cytotoxic activities [141].

### 4.2. Mangrove

Mangroves are also important sources of a class of marine diterpenoid compounds. However, few relevant studies have been conducted. Sixteen mangrove-derived diterpenoids are listed in this section.

**Figure 51:**
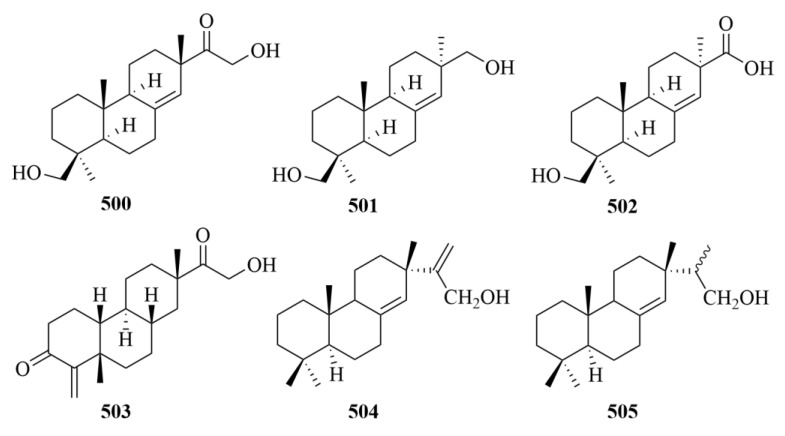
Chemical structures of diterpenoids from mangroves (**500**–**505**).

The isolation of four undescribed diterpenes, tagalons A–D (**500**–**503**, Figure 51), was revealed from the Chinese mangrove, *Ceriops tagal* [142]. To the human breast cancer cell line MT-1, compounds **502** and **503** showed selective cytotoxicities with IC_50_ values of 3.75 and 8.07 µM, respectively [142].

The structures of two isopimarane diterpenes were revealed from the chloroform extract of the roots of *Ceriops tagal* from Maruhubi Mangrove Reserve in Zanzibar, Tanzania, and named isopimar-8(14)-en-16-hydroxy-15-one (**504**, Figure 51) and isopimar-8(14)-en-15,16-diol (**505**, Figure 51) [143]. Their antibacterial activities were evaluated on five Gram-positive and five Gram-negative bacterial strains [143]. The result showed that compound **504** has low antibacterial activity against *Bacillus cereus*, *Staphylococcus aureus*, and *Micrococcus kristinae* (each with MIC values of 100 µg/mL) and lower activity towards *Streptococcus pyrogens* and *Salmonella pooni* (MIC = 500, 250 µg/mL), with chloramphenicol serving as the positive control (each with MIC values of 1.0 µg/mL) [143]. However, compound **505** did not show antibacterial activity in the test [143].

**Figure 52:**
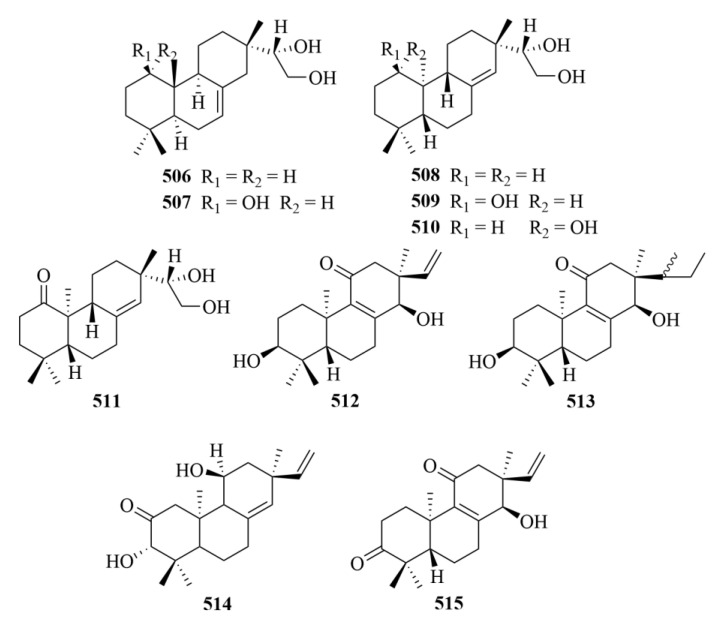
Chemical structures of diterpenoids from mangroves (**506**–**515**).

The methanol extracts of the stems of marine mangrove *Bruguiera gymnorrhiza* from Xiamen, China, were the source of two isopimarane diterpenes, compounds **506** and **507** (Figure 52) [144]. With an IC_50_ value of 22.9 µM, compound **506** displayed moderate cytotoxicity against K562 chronic myeloid leukemia cells. However, no activity was exhibited by compound **507** [144].

The further study gained four ent-pimarane diterpenoids (**508**–**511**, Figure 52) from the same mangrove plant [144]. The cytotoxic activity against K562, HeLa, and L-929 (mouse fibroblasts) cell lines of compound **508**–**511** were tested, and only compound **510** showed weaker cytotoxicity on L-929 (IC_50_ = 30.6 µM) [144].

Three ent-isopimarane diterpenes (**512**–**514**, Figure 52), agallochaols A (**512**) and B (**513**) and compound **514**, together with agallochaone A (**515**, Figure 52), were obtained from the Chinese mangrove *Excoecaria agallocha* L. [145,146,147]. Among them, compounds **512**–**513** were isolated from the MeOH extract of the stems and leaves of the mangrove, and they had no activity against A-549 human lung cancer cells, whereas the MeOH extract of *Excoecaria agallocha* L. exhibited weak antitumor activity [145,146,147].

## 5. Bioactivities of Diterpenoids from Marine-Derived Fungi

**Figure 53:**
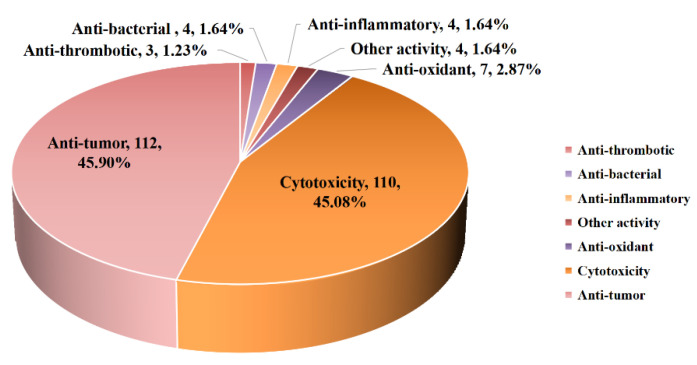
Bioactivities of marine-derived diterpenoids.

Natural diterpenoids have attracted considerable interest because of their powerful pharmacological activities, including cytotoxic, anti-inflammatory, anticancer, analgesic, antitumor, and antidiabetic activities [148,149,150,151,152,153], which are of great significance for drug research for conditions such as tuberculosis (TB) [154,155], leukemia [156], and breast cancer [157]. Through our classification of 515 compounds from the year 2000 to the year 2024, there are 244 compounds that demonstrated bioactivities, and the surprising potential of the anti-tumor and cytotoxic activity of marine-derived diterpenoids is demonstrated, for 112 compounds showed significant anti-tumor activity (45.90%), and another 110 compounds exhibited potent cytotoxicity (45.08%). In addition, other various bioactivities are also displayed by some diterpenes, such as anti-oxidant activity (2.87%), anti-inflammatory activity (1.64%), anti-bacterial activity (1.64%), and anti-thrombotic activity (1.23%) (Figure 53). The bioactivities of these marine fungi-derived diterpenoids are elaborated in this work (Table 1, Table 2 and Table 3). In addition, the structure-activity relations of active compounds are included as well.

**Table 1:** Marine fungi-derived compounds with various bioactivities.

Source	NO.	Compound	Producing Organism	Extract/Fraction	Activity	References
Sediment	**1**	Harzianol J	*Trichoderma* sp. SCSIOW21	BuOH extract	An anti-inflammatory effect with 81.8% and NO inhibition at 100 µM	[21]
Sediment	**2**	Harzianol A	*Trichoderma* sp. SCSIOW21	BuOH extract	An anti-inflammatory effect with 46.8% and NO inhibition at 100 µM	[21]
Sediment	**7**	Harzianol O	*Trichoderma* sp. SCSIOW21	BuOH extract	An anti-inflammatory effect with 50.5% and NO inhibition at 100 µM	[21]
Sediment	**8**	13β-hydroxy conidiogenone C	*Penicillium* sp. TJ403-2	EtOAc extract	A significant anti-inflammatory activity against LPS-induced NO production in RAW 264.7 cells, with an IC_50_ value of 2.19 µM	[14]
Sediment	**11**	Spirograterpene A	*Penicillium granulatum* MCCC 3A00475	EtOAc extract	Anti-allergic effects on immunoglobulin E (IgE)-mediated rat mast RBL-2H3 cells with the inhibition rate of 18% at 20 µg/mL	[15]
Sediment	**12**	Conidiogenol C	*Penicillium* sp. YPGA11	EtOAc extract	Weak inhibitory effects with inhibition rates below 36% at an initial concentration of 50 µM against five esophageal HTCLs (EC109, KYSE70, EC9706, KYSE30, and KYSE450)	[16]
Sediment	**13**	Conidiogenol D	*Penicillium* sp. YPGA11	EtOAc extract	Weak inhibitory effects against five esophageal HTCLs (EC109, KYSE70, EC9706, KYSE30, and KYSE450) with an IC_50_ value ranging from 36.80 to 54.7 µM	[16]
Sediment	**14**	Conidiogenone L	*Penicillium* sp. YPGA11	EtOAc extract	Weak inhibitory effects with inhibition rates below 36% at an initial concentration of 50 µM against five esophageal HTCLs (EC109, KYSE70, EC9706, KYSE30, and KYSE450)	[16]
Sediment	**15**	Xylarinonericin E	*Penicillium* sp. H1	EtOAc extract	A moderate antifungal activity against *Fusarium oxysporum f.* sp. *cubense*, with an MIC value of 32.0 µM	[17]
Sediment	**16**	Conidiogenone B	*Penicillium* sp. F23-2	EtOAc extract	Weak cytotoxicities against the A-549 cell line and HL-60 cell line with IC_50_ values of 40.3 and 28.2 µM, respectively	[19]
Sediment	**17**	Conidiogenone C	*Penicillium* sp. F23-2	EtOAc extract	Exceptional potency against the HL-60 and BEL-7402 cell lines, with IC_50_ values of 0.038 and 0.97 µM	[19]
Sediment	**18**	Conidiogenone D	*Penicillium* sp. F23-2	EtOAc extract	Cytotoxicities against the A-549, HL-60, BEL-7402, and MOLT-4 cell lines with IC_50_ values of 9.3, 5.3, 11.7, and 21.1 µM, respectively	[19]
Sediment	**19**	Conidiogenone E	*Penicillium* sp. F23-2	EtOAc extract	Significant cytotoxicities against the A-549 cell line and HL-60 cell line with IC_50_ values of 15.1 and 8.5 µM, respectively	[19]
Sediment	**20**	Conidiogenone F	*Penicillium* sp. F23-2	EtOAc extract	Cytotoxicities against the A-549, HL-60, BEL-7402, and MOLT-4 cell lines with IC_50_ values of 42.2, 17.8, 17.1, and 25.8 µM, respectively	[19]
Sediment	**21**	Conidiogenone G	*Penicillium* sp. F23-2	EtOAc extract	Cytotoxicities against the A-549, HL-60, BEL-7402, and MOLT-4 cell lines with IC_50_ values of 8.3, 1.1, 43.8, and 4.7 µM, respectively	[19]
Sediment	**22**	Penicindopene A	*Penicillium* sp. YPCMAC1	EtOAc extract	Moderate cytotoxicities against the A-549 and HeLa cell lines with IC_50_ values of 15.2 and 20.5 µM, respectively	[19]
Sediment	**23**	Trichosordarin A	*Trichoderma harzianum* R5	CH_2_Cl_2_ and MeOH (1:1, *v*/*v*) extract	Toxicity towards the marine zooplankton *A. salina* with an LC_50_ value of 233 µM; weak inhibitory activities against two marine phytoplankton species (*Amphidinium carterae* and *Phaeocysti globosa*), with inhibition rates at 100 µg/mL of 20.6% and 8.1%, respectively	[20]
Sediment	**24**	Asperolide D	*Aspergillus wentii* SD-310	EtOAc extract	Moderate inhibitory activities towards the aquatic pathogens *Edwardsiella tarda* and the plant bacteria *Fusarium graminearum* with MIC values of 16 and 2 µg/mL, respectively; inhibitory activities against aquatic bacteria *Edwardsiella tarda*, *Micrococcus luteus*, *Pseudomonas aeruginosa*, *Vibrio harveyi*, and *V. parahemolyticus*, with the same MIC value of 4.0 µg/mL	[22]
Sediment	**25**	Asperolide E	*Aspergillus wentii* SD-310	EtOAc extract	Cytotoxicities against HeLa, MCF-7, and NCI-H446 cell lines, with IC_50_ values of 10.0, 11.0, and 16.0 µM, respectively, and moderate activity against *Edwardsiella tarda*, with an MIC value of 16 µg/mL	[22]
Sediment	**26**	Wentinoid A	*Aspergillus wentii* SD-310	EtOAc extract	Inhibitory activities against aquatic bacteria *Edwardsiella tarda*, *Micrococcus luteus*, *Pseudomonas aeruginosa*, *Vibrio harveyi*, and *V. parahemolyticus*, with the same MIC value of 4.0 µg/mL; selective inhibition against four plant pathogenic fungi (*Phytophthora parasitica*, *Fusarium oxysporum f.* sp. *lycopersici*, *Fusarium graminearum*, and *Botryosphaeria dothidea*)	[23]
Sediment	**27**	Wentinoid B	*Aspergillus wentii* SD-310	EtOAc extract	Inhibitory activities against aquatic bacteria *Edwardsiella tarda*, *Micrococcus luteus*, *Pseudomonas aeruginosa*, *Vibrio harveyi*, and *V. parahemolyticus*, with the same MIC value of 4.0 µg/mL	[23]
Sediment	**28**	Wentinoid C	*Aspergillus wentii* SD-310	EtOAc extract	Inhibitory activities against aquatic bacteria *Edwardsiella tarda*, *Micrococcus luteus*, *Pseudomonas aeruginosa*, *Vibrio harveyi*, and *V. parahemolyticus*, with the same MIC value of 4.0 µg/mL; notable inhibitory activities towards the plant bacteria *Fusarium graminearum* with MIC values of 4.0 µg/mL	[23]
Sediment	**33**	Aspewentin D	*Aspergillus wentii* SD-310	EtOAc extract	Significant inhibition against aquatic pathogens (*M. luteus*, *E. tarda*, *V. harveyi*, *P. aeruginosa*, and *V. parahemolyticus*), each with MIC values of 4.0 µg/mL, compared with the positive control chloramphenicol, with MIC values of 8.0 µg/mL; potent activity against plant pathogenic fungi *F. graminearum* with MIC values of 2.0 µg/mL.	[24]
Sediment	**35**	Aspewentin F	*Aspergillus wentii* SD-310	EtOAc extract	Great inhibition against aquatic pathogens (*M. luteus*, *E. tarda*, *V. harveyi*, *P. aeruginosa*, and *V. parahemolyticus*), each with MIC values of 4.0 µg/mL, compared with the positive control chloramphenicol, with the MIC values of 8.0 µg/mL	[24]
Sediment	**36**	Aspewentin G	*Aspergillus wentii* SD-310	EtOAc extract	Significant inhibition against aquatic pathogens (*M. luteus*, *E. tarda*, *V. harveyi*, *P. aeruginosa*, and *V. parahemolyticus*), each with MIC values of 4.0 µg/mL, compared with the positive control chloramphenicol, with the MIC values of 4.0 µg/mL	[24]
Sediment	**37**	Aspewentin H	*Aspergillus wentii* SD-310	EtOAc extract	Significant inhibition against aquatic pathogens (*M. luteus*, *E. tarda*, *V. harveyi*, *P. aeruginosa*, and *V. parahemolyticus*), each with MIC values of 4.0 µg/mL, compared with the positive control chloramphenicol, with the MIC values of 4.0 µg/mL	[24]
Sediment	**38**	Aspewentin I	*Aspergillus wentii* SD-310	EtOAc extract	Notable inhibitory activities against three marine bacteria (*E. tarda*, *V. harveyi*, and *V. parahaemolyticus*), with an MIC value of 8.0 µg/mL; inhibitory activities toward zoonotic pathogens between human and aquatic animals, such as *Escherichia coli*, *Edwardsiella tarda*, *Vibrio harveyi*, and *V. parahaemolyticus*; great inhibition against aquatic pathogens (*M. luteus*, *E. tarda*, *V. harveyi*, *P. aeruginosa*, and *V. parahemolyticus*), each with MIC values of 4.0 µg/mL, compared with the positive control chloramphenicol, with the MIC values of 1.0 µg/mL	[25]
Sediment	**39**	Aspewentin J	*Aspergillus wentii* SD-310	EtOAc extract	Notable inhibitory activities against three marine bacteria (*E. tarda*, *V. harveyi*, and *V. parahaemolyticus*), with an MIC value of 8.0 µg/mL; inhibitory activities toward zoonotic pathogens between human and aquatic animals, such as *Escherichia coli*, *Edwardsiella tarda*, *Vibrio harveyi*, and *V. parahaemolyticus*; potent inhibition against aquatic pathogens (*M. luteus*, *E. tarda*, *V. harveyi*, *P. aeruginosa*, and *V. parahemolyticus*)	[25]
Sediment	**40**	Aspewentin K	*Aspergillus wentii* SD-310	EtOAc extract	Activity against pathogenic bacteria	[25]
Sediment	**41**	Aspewentin L	*Aspergillus wentii* SD-310	EtOAc extract	Activity against pathogenic bacteria	[25]
Sediment	**42**	Aspewentin M	*Aspergillus wentii* SD-310	EtOAc extract	Activity against *F. graminearum* with an MIC value of 4.0 µg/mL	[25]
Sediment	**44**	Libertellenone A	*Eutypella scoparia*	EtOAc extract	Selective cytotoxic activities against SF-268, MCF-7, and NCI-H460 (IC_50_ = 20.5, 12.0, and 40.2 µM)	[26]
Sediment	**47**	Diaporthein B	*Eutypella scoparia*	EtOAc extract	Significant cytotoxicity against SF-268, MCF-7, and NCI-H460 (IC_50_ = 9.2, 4.4, and 9.9 µM)	[26]
Sediment	**48**	11-deoxydiaporthein A	*Eutypella scoparia*	EtOAc extract	Moderate cytotoxicity against the MCF-7 cell line with IC_50_ = 38.8 µM	[26]
Sediment	**49**	Scopararane C	*Eutypella scoparia*	EtOAc extract	Moderate cytotoxicity against the MCF-7 cell line with IC_50_ = 16.4 µM	[26]
Sediment	**50**	Scopararane D	*Eutypella scoparia* FS26	EtOAc extract	Cytotoxic activity towards the MCF-7 cell line with an IC_50_ value of 25.6 µM; moderate cytotoxic activities against SF-268 and NCI-H460 cell lines with IC_50_ values of 43.5 µM and 46.1 µM.	[27]
Sediment	**51**	Scopararane E	*Eutypella scoparia* FS26	EtOAc extract	Cytotoxic activity towards the MCF-7 cell line with IC_50_ values of 74.1 µM	[27]
Sediment	**53**	Scopararane G	*Eutypella scoparia* FS26	EtOAc extract	Cytotoxic activities towards the MCF-7 cell line with IC_50_ values of 85.5 µM	[27]
Sediment	**55**	Scopararane I	*Eutypella* sp. FS46	EtOAc extract	Moderate inhibitory activity against NCI-H460 and SF268 cell lines with IC_50_ values of 13.59 and 25.31 µg/mL	[28]
Coral	**56**	Harzianelactone A	*Trichoderma harzianum* XS20090075	EtOAc extract	Notable activities against seedling growth of amaranth and lettuce	[29]
Coral	**57**	Harzianelactone B	*Trichoderma harzianum* XS20090075	EtOAc extract	Notable activities against seedling growth of amaranth and lettuce	[29]
Coral	**58**	Harzianone A	*Trichoderma harzianum* XS20090075	EtOAc and CH_2_Cl_2_-MeOH (*v*/*v*, 1:1) extract	Notable activities against seedling growth of amaranth and lettuce	[29]
Coral	**59**	Harzianone B	*Trichoderma harzianum* XS20090075	EtOAc and CH_2_Cl_2_-MeOH (*v*/*v*, 1:1) extract	Notable activities against seedling growth of amaranth and lettuce	[29]
Coral	**60**	Harzianone C	*Trichoderma harzianum* XS20090075	EtOAc and CH_2_Cl_2_-MeOH (*v*/*v*, 1:1) extract	Notable activities against seedling growth of amaranth and lettuce	[29]
Coral	**61**	Harzianone D	*Trichoderma harzianum* XS20090075	EtOAc and CH_2_Cl_2_-MeOH (*v*/*v*, 1:1) extract	Notable activities against seedling growth of amaranth and lettuce	[29]
Coral	**62**	Harziane	*Trichoderma harzianum* XS20090075	EtOAc and CH_2_Cl_2_-MeOH (*v*/*v*, 1:1) extract	Notable activities against seedling growth of amaranth and lettuce	[29]
Coral	**63**	Moriniafungusn B	*Curvularia hawaiiensis* TA2615	EtOAc extract	Diverse antifungal activity	[30]
Coral	**64**	Moriniafungusn C	*Curvularia hawaiiensis* TA2615	EtOAc extract	Diverse antifungal activity	[30]
Coral	**65**	Moriniafungusn D	*Curvularia hawaiiensis* TA2615	EtOAc extract	Diverse antifungal activity	[30]
Coral	**66**	Moriniafungusn E	*Curvularia hawaiiensis* TA2615	EtOAc extract	Potent antifungal activity against *Candida albicans* ATCC10231 with an MIC value of 2.9 µM	[30]
Coral	**67**	Moriniafungusn F	*Curvularia hawaiiensis* TA2615	EtOAc extract	Diverse antifungal activity	[30]
Coral	**68**	Moriniafungusn G	*Curvularia hawaiiensis* TA2615	EtOAc extract	Diverse antifungal activity	[30]
Coral	**69**	Sordaricin B	*Curvularia hawaiiensis* TA2615	EtOAc extract	Diverse antifungal activity	[30]
Coral	**71**	Stachatranone B	*Stachybotrys chartarum* TJ403-SS6	EtOAc extract	An inhibitory effect against *Acinetobacter baumannii* (MIC = 16 µg/mL) and an inhibitory effect against *Enterococcus faecalis* (MIC = 32 µg/mL)	[31]
Sponge	**73**	Trichodermanin C	*Trichoderma harzianum* OUPS-111D-4	EtOAc extract	Potent activities towards three cancer cell lines, P388, HL-60, and L1210, with IC_50_ values ranging from 6.8 to 7.9 µM	[32,33]
Sponge	**75**	Trichodermanin E	*Trichoderma harzianum* OUPS-111D-4	EtOAc extract	Moderate activities towards three cancer cell lines, P388, HL-60, and L1210	[32,33]
Sponge	**76**	Trichodermanin F	*Trichoderma harzianum* OUPS-111D-4	EtOAc extract	Moderate activities towards three cancer cell lines, P388, HL-60, and L1210	[32,33]
Sponge	**80**	Compound JBIR-65	*Actinomadura* sp.	EtOAc extract	An ability to protect neuronal hybridoma N18-RE-105 cells from L-glutamate toxicity, with an EC_50_ value of 31 µM	[35]
Sponge	**83**	Ascandinine C	*Aspergillus candidus* HDN15-152	EtOAc extract	Anti-influenza virus A (H1N1) activity with an IC_50_ value of 26 µM, with ribavirin served as the positive control (IC_50_ = 31 µM)	[36]
Sponge	**84**	Ascandinines D	*Aspergillus candidus* HDN15-152	EtOAc extract	Strong cytotoxic activity against HL-60 cells with an IC_50_ value of 7.8 µM	[36]
Sponge	**85**	Myrocin A	*Arthrinium* sp.	Methanolic extract	Vascular endothelial growth factor A (VEGF-A)-dependent endothelial cell sprouting (IC_50_ = 3.7 µM); notable antiproliferative activities against L5178Y (mouse lymphoma) tumor cell line (IC_50_ = 2.05 µM); no inhibitory activity for the protein kinase and weak activities against K-562, A2780 (human ovarian cancer line), and A2780CisR (cisplatin-resistant human ovarian cancer cells) with IC_50_ values of 50.3, 41.3, and 66.0 µM, with cisplatin used as the positive control (IC_50_ = 7.80, 0.80, and 8.40 µM).	[37]
Sponge	**88**	Arthritis D	*Arthrinium* sp.	Methanolic extract	Vascular endothelial growth factor A (VEGF-A)-dependent endothelial cell sprouting (IC_50_ = 2.6 µM); notable antiproliferative activities against L5178Y (mouse lymphoma) tumor cell line (IC_50_ = 2.74 µM)	[37]
Sponge	**89**	Myrocin D	*Arthrinium* sp.	Methanolic extract	No inhibitory activity for the protein kinase and weak activities against K-562, A2780 (human ovarian cancer line), and A2780CisR (cisplatin-resistant human ovarian cancer cells) with IC_50_ values of 42.0, 28.2, and 154.7 µM, respectively, with cisplatin used as the positive control (IC_50_ = 7.80, 0.80, and 8.40 µM).	[37]
Algae	**95**	Trichocitrin	*Trichoderma citrinoviride* cf-27	CH_2_Cl_2_ and MeOH (1:1, *v*/*v*) extract	An 8.0 mm inhibition zone against *Escherichia coli* at 20 µg/disk	[43,44]
Algae	**96**	Citrinovirin	*Dictyopteris prolifera*	EtOAc extract	Inhibitory activity towards *S. aureus* (MIC = 12.4 µg/mL); toxicity against the marine zooplankton *Artemia salina* (LC_50_ = 65.6 µg/mL); a 14.1–37.2% inhibition of three marine phytoplankton species (*C. marina*, *H. akashiwo*, and *P. donghaiense*) at 100 µg/mL.	[43,44]
Algae	**97**	(+)-wickerol A	*Trichoderma asperellum* d1-34	EtOAc extract	Inhibitory activity against *E. coli* and *S. aureus*, with the same inhibitory diameters of 8.0 mm at 30 µg/disc; lethal activity against *A. salina* with an LC_50_ value of 12.0 µg/mL	[45]
Algae	**98**	3R-hydroxy-9R,10R-dihydroharzianone	*Trichoderma harzianum* X-5	CH_2_Cl_2_ and MeOH (1:1, *v*/*v*) extract	Inhibitory activity against *Chattonella marina* with an IC_50_ value of 7.0 µg/mL	[46]
Algae	**99**	11Rmethoxy-5,9,13-proharzitrien-3-ol	*Trichoderma harzianum* X-5	EtOAc extract	Notable inhibitory effect on the growth of all four kinds of phytoplankton, with IC_50_ values of 1.2, 1.3, 3.2, and 4.3 µg/mL, respectively, with K_2_Cr_2_O_7_ as a positive control (IC_50_ = 0.46, 0.98, 0.89, and 1.9 µM)	[46]
Algae	**103**	Deoxytrichoderma-erin	*Trichoderma longibrachiatum* A-WH-20-2	EtOAc extract	Strong inhibition offour marine phytoplankton strains (*C. marina*, *H. akashiwo*, *K. veneficum*, and *P. donghaiense*) with IC_50_ values ranging from 0.53 to 2.7 µg/mL; toxicity against the marine zooplankton *A. salina* with a LC_50_ value of 19 µg/mL	[48]
Algae	**104**	3S-hydroxyharzianone	*Trichoderma asperellum* A-YMD-9-2	CH_2_Cl_2_ and MeOH (1:1, *v*/*v*) extract	Significant inhibition of four marine phytoplankton strains (*C. marina*, *H. akashiwo*, *K. veneficum*, and *P. donghaiense*) with IC_50_ values ranging from 3.1 to 7.7 µg/mL; weak inhibition against five marine-derived pathogenic bacteria (four different strains of *Vibrio* and a *P. citrea*), at 40 µg/disc	[49]
Algae	**105**	Harzianone	*Trichoderma longibrachiatum*	/	82.6% of lethality in brine shrimp (*Artemia salina* L.) larvae at 100 µg/mL and exhibition of antibacterial activity against *Escherichia coli* and *Staphylococcus aureus* at 30 µg/disk, with inhibitory diameters of 8.3 and 7.0 mm, respectively	[50]
Algae	**113**	19-hydroxypenitrem A	*Aspergillus nidulans* EN-330	Acetone extract	Antibacterial activity against pathogens *Edwardsiella tarda*, *Vibrio anguillarum*, *Escherichia coli*, and *Staphylococcus aureus*, with MIC values of 16, 32, 16, and 16 µg/mL, respectively	[55]
Algae	**115**	Compound **115**	*Aspergillus wentii* na-3	CHCl_3_ and MeOH (1:1, *v*/*v*) extract	Activities against two marine phytoplankton species (*Chattonella marina* and *Heterosigma akashiwo*) with LC_50_ values of 0.81 and 2.88 µM	[56]
Algae	**116**	Compound **116**	*Aspergillus wentii* na-3	CHCl_3_ and MeOH (1:1, *v*/*v*) extract	Inhibitory activities against the marine zooplankton *Artemia salina* with an LC_50_ of 6.36 µM	[56]
Mangrove	**129**	(9R, 10R)-dihydro-harzianone	*Trichoderma* sp. Xy24	/	Selective cytotoxicities toward the HeLa and MCF-7 cell lines with IC_50_ values of 30.1 and 30.7 µM	[59]
Mangrove	**130**	Harzianelactone	*Trichoderma* sp. Xy24	/	Inactive cytotoxicities to the HeLa and MCF-7 cell lines with IC_50_ values of 10 mM	[59]
Mangrove	**132**	Anthcolorin H	*Aspergillus versicolor*	EtOAc extract	Weak activity against HeLa cells, with an IC_50_ value of 43.7 µM	[60]
Mangrove	**133**	Penicilindole A	*Eupenicillium* sp. HJ002	EtOAc extract	Potent activities against human A-549 and HepG2 cell lines (IC_50_ = 5.5, 1.5 µM), with adriamycin used as the positive control (IC_50_ = 0.002, 0.1 µM), and 36.8 and 76.9 µM, respectively, for 5-fluoracil	[62]
Mangrove	**136**	Compound **141**	*Penicillium camemberti* OUCMDZ-1492	EtOAc extract	Weak activities against the H1N1 virus, with IC_50_ values of 28.3 µM	[61]
Mangrove	**137**	Compound **142**	*Penicillium camemberti* OUCMDZ-1492	EtOAc extract	Weak activities against the H1N1 virus, with IC_50_ values of 38.9 µM	[61]
Mangrove	**138**	Compound **143**	*Penicillium camemberti* OUCMDZ-1492	EtOAc extract	Weak activities against the H1N1 virus, with IC_50_ values of 32.2 µM	[61]
Mangrove	**140**	Compound **145**	*Penicillium camemberti* OUCMDZ-1492	EtOAc extract	Weak activities against the H1N1 virus, with IC_50_ values of 73.3 µM	[61]
Mangrove	**142**	Rhizovarin A	*Mucor irregularis* QEN-189	MeOH and EtOAc extract	Moderate activities towards the A-549 cancer cell line, with IC_50_ values of 11.5 µM; notable activities against the HL-60 cancer cell line with IC_50_ values of 9.6 µM	[63]
Mangrove	**143**	Rhizovarin B	*Mucor irregularis* QEN-189	MeOH and EtOAc extract	Moderate activities towards the A-549 cancer cell line, with IC_50_ values of 6.3 µM; notable activities against the HL-60 cancer cell line with IC_50_ values of 5.0 µM	[63]
Mangrove	**147**	Rhizovarin F	*Mucor irregularis* QEN-189	MeOH and EtOAc extract	Moderate activities towards the A-549 cancer cell line, with an IC_50_ value of 9.2 µM	[63]
Miscellaneous	**151**	Penitholabene	*Penicillium thomii* YPGA3	EtOAc extract	An inhibitory effect against the α-glucosidasewith an IC_50_ value of 282 µM	[65]
Miscellaneous	**153**	6-hydroxylaspalinine	*Penicillium* sp. AS-79	EtOAc extract	Activity against the aquatic pathogen *Vibrio parahaemolyticus* with an MIC of 64.0 µg/mL	[66]
Miscellaneous	**155**	Compound **155**	*Penicillium* sp. KFD28	EtOAc extract	Potent inhibitory activities against protein tyrosine phosphatase (PTP1B) with IC_50_ values of 1.7 µM	[67]
Miscellaneous	**156**	Compound **156**	*Penicillium* sp. KFD28	EtOAc extract	Potent inhibitory activities againstprotein tyrosine phosphatase (PTP1B) with IC_50_ values of 2.4 µM	[67]
Miscellaneous	**159**	Compound **159**	*Penicillium* sp. KFD28	EtOAc extract	Potent inhibitory activities against protein tyrosine phosphatase (PTP1B) with IC50 values of 14 µM	[67]
Miscellaneous	**160**	Compound **160**	*Penicillium* sp. KFD28	EtOAc extract	Potent inhibitory activities against protein tyrosine phosphatase (PTP1B) with IC_50_ values of 27 µM	[67]
Miscellaneous	**162**	Compound **162**	*Penicillium* sp. KFD28	EtOAc extract	Potent inhibitory activities againstprotein tyrosine phosphatase (PTP1B) with IC_50_ values of 23 µM	[67]
Miscellaneous	**163**	Compound **163**	*Penicillium* sp. KFD28	EtOAc extract	Potent inhibitory activities against protein tyrosine phosphatase (PTP1B) with IC_50_ values of 31.5 µM	[67]
Miscellaneous	**167**	Compound **167**	*Penicillium* sp. KFD28	EtOAc extract	Weak activity against HeLa cells with an IC_50_ value of 36.3 µM	[67]
Miscellaneous	**168**	Compound **168**	*Penicillium* sp. KFD28	EtOAc extract	Potent inhibitory activities against protein tyrosine phosphatase (PTP1B) with IC_50_ values of 9.5 µM	[67]
Miscellaneous	**171**	Botryotins A	*Botryotinia fuckeliana* MCCC	CHCl_3_/MeOH (1:1) extract	Being inactive against six HTCLs (HL-60, BEL-7402, BIU-87, PANC-1, HeLa-S3, and ECA109), each with the IC_50_ less than 20 µM; moderate antiallergic activity in RBL-2H3 cells with an IC_50_ value of 0.2 mM	[68,69]
Miscellaneous	**172**	Botryotins B	*Botryotinia fuckeliana* MCCC	CHCl_3_/MeOH (1:1) extract	Being inactive against six HTCLs (HL-60, BEL-7402, BIU-87, PANC-1, HeLa-S3, and ECA109), each with IC_50_ values less than 20 µM	[68,69]
Miscellaneous	**173**	Botryotins C	*Botryotinia fuckeliana* MCCC	CHCl_3_/MeOH (1:1) extract	Being inactive against six HTCLs (HL-60, BEL-7402, BIU-87, PANC-1, HeLa-S3, and ECA109), each with IC_50_ values less than 20 µM	[68,69]
Miscellaneous	**174**	Botryotins D	*Botryotinia fuckeliana* MCCC	CHCl_3_/MeOH (1:1) extract	Being inactive against six HTCLs (HL-60, BEL-7402, BIU-87, PANC-1, HeLa-S3, and ECA109), each with IC_50_ values less than 20 µM	[68,69]
Miscellaneous	**175**	Botryotins E	*Botryotinia fuckeliana* MCCC	CHCl_3_/MeOH (1:1) extract	Being inactive against six HTCLs (HL-60, BEL-7402, BIU-87, PANC-1, HeLa-S3, and ECA109), each with IC_50_ less than 20 µM	[68,69]
Miscellaneous	**176**	Botryotins F	*Botryotinia fuckeliana* MCCC	CHCl_3_/MeOH (1:1) extract	Being inactive against six HTCLs (HL-60, BEL-7402, BIU-87, PANC-1, HeLa-S3, and ECA109), each with IC_50_ less than 20 µM	[68,69]
Miscellaneous	**177**	Botryotins G	*Botryotinia fuckeliana* MCCC	CHCl_3_/MeOH (1:1) extract	Being inactive against six HTCLs (HL-60, BEL-7402, BIU-87, PANC-1, HeLa-S3, and ECA109), each with IC_50_ less than 20 µM	[68,69]
Miscellaneous	**178**	Botryotins H	*Botryotinia fuckeliana* MCCC	CHCl_3_/MeOH (1:1) extract	Being inactive against six HTCLs (HL-60, BEL-7402, BIU-87, PANC-1, HeLa-S3, and ECA109), each with IC_50_ less than 20 µM	[68,69]
Miscellaneous	**179**	A1	*Botryotinia fuckeliana* MCCC	EtOAc extract	Useful as a potent cytotoxic lead compound due to its notable activities against T24 and HL-60 cells (IC_50_ = 2.5, 6.1 µM)	[70]
Miscellaneous	**252**	Micromonohalimane B	*Micromonospora* sp. WMMC-218	Acetone extract	Inhibition of the methicillin-resistant *Staphylococcus aureus* with an MIC value of 40 µg/mL	[72]
Miscellaneous	**253**	Virescenosides Z9	*Acremonium striatisporum* KMM 4401	CHCl_3_-EtOH (2:1, *v*/*v*, 2.5 L) extract	Observably decreased ROS production in macrophages under 10 µM LPS stimulation.	[73]
Miscellaneous	**254**	Virescenoside Z10	*Acremonium striatisporum* KMM 4401	CHCl_3_-EtOH (2:1, *v*/*v*, 2.5 L) extract	Observably decreased ROS production in macrophages under 10 µM LPS stimulation, inducing downregulation of ROS production by 45%, and decreased NO production in LPS-stimulated macrophages at a concentration of 1 µM	[73]
Miscellaneous	**256**	Virescenosides Z12	*Acremonium striatisporum* KMM 4401	CHCl_3_-EtOH (2:1, *v*/*v*, 2.5 L) extrac	Observably decreased ROS production in macrophages under 10µM LPS stimulation	[73]
Miscellaneous	**257**	Virescenoside Z13	*Acremonium striatisporum* KMM 4401	CHCl_3_-EtOH (2:1, *v*/*v*, 2.5 L) extract	Observably decreased ROS production in macrophages under 10µM LPS stimulation, and decreased the NO production in LPS-stimulated macrophages at a concentration of 1 µM	[73]
Miscellaneous	**263**	(2R, 4bR, 6aS, 12bS, 12cS, 14aS)-4b-Deoxyβ-aflatrem	*Aspergillus flavus* OUCMDZ-2205	EtOAc extract	Cytotoxicity against the A-549 cell cycle in the *S* phase with IC_50_ values of 10 µM; inhibition against the kinase PKC-β with an IC_50_ value of 15.6 µM	[74]
Miscellaneous	**264**	(2R, 4bS), 6aS, 12bS, 12cR)-9-Isopentenylpaxillin-e D	*Aspergillus flavus* OUCMDZ-2205*i*	EtOAc extract	Cytotoxicity against the A-549 cell cycle in the *S* phase with IC_50_ values of 10 µM	[74]
Miscellaneous	**265**	(3R, 9S, 12R, 13S, 17S, 18S)-2-carbonyl3hydroxylemeniveol	*Aspergillus versicolor* ZZ761	/	Activity against *Escherichia coli* and *Candida albicans* with MIC values of 20.6 and 22.8 µM, respectively	[75]
Miscellaneous	**266**	Noonindole A	*Aspergillus noonimiae* CMB-M0339	EtOAc extract	Moderate antifungal activity against the fungi *Candida albicans*	[76]
Miscellaneous	**273**	Compound **273**	*Epicoccum* sp. HS-1	Ethyl acetate (1:1, *v*/*v*) extract	Inhibition of α-glucosidase with IC_50_ values of 4.6 µM, higher than the p.c. resveratrol, with IC_50_ = 31.2 µM	[78]
Miscellaneous	**274**	Roussoellol C	*Talaromyces purpurogenus* PP-414	EtOAc extract	Cytotoxic activity against the MCF-7 cells with an IC_50_ of 6.5 µM	[79]
Miscellaneous	**275**	Libertellenone B	*Libertella* sp.	EtOAc extract	Weak activities against HCT-116 (human adenocarcinoma cell line) (IC_50_ = 15 µM)	[35]
Miscellaneous	**276**	Libertellenone C	*Libertella* sp.	EtOAc extract	Weak activities against HCT-116 (human adenocarcinoma cell line) (IC_50_ = 53 µM)	[35]
Miscellaneous	**277**	Libertellenone D	*Libertella* sp.	EtOAc extract	Significant cytotoxicity against HCT-116 (human adenocarcinoma cell line) (IC_50_ = 53 µM)	[35]
Miscellaneous	**279**	Eutypellenoid B	*Eutypella* sp. D-1	CH_2_Cl_2_/CH_3_OH (1:1, *v*/*v*) extract	Antibacterial activities against *Staphylococcus aureus* and *Escherichia coli* with MIC values of 8 and 8 µg/mL; antifungal activities against *Candida parapsilosis*, *Candida albicans*, *Candida glabrata*, and *Candida tropicalis* with MIC values of 8, 8, 16, and 32 µg/mL, respectively; moderate cytotoxic activity against the HCT-116 cell line with IC_50_ value of 3.7 µM	[81]

## 6. Conclusions and Perspectives

Diterpenoids are widely distributed in marine organisms and exhibit diverse pharmacological activities. This paper offers a comprehensive review of 515 diterpenoids discovered in the marine field over the past last two decades. Based on their origin, the diterpenoids from marine organisms are divided into three distinct groups, namely 281 marine fungi-sourced diterpenoids, 162 marine invertebrate-derived diterpenoids, and 72 marine plant-associated diterpenoids. We demonstrate the chemical structure of these compounds and elucidate their significance in biological activity. Marine diterpenoids exhibit a plethora of activities, encompassing significant anti-tumor activity and cytotoxicity, anti-oxidant activity, anti-inflammatory activity, anti-bacterial activity, and anti-thrombotic activity, among others. Consequently, marine-derived diterpenoids undeniably hold potential as candidates for novel drug development.

## Figures and Tables

**Table 2:** Marine invertebrate-derived compounds with various bioactivities.

Source	NO.	Compound	Producing Organism	Extract/Fraction	Activity	References
Sponge	**282**–**284**	Spongenolactones A-C	Red Sea sponge *Spongia* sp.	EtOAc/MeOH/CH_2_Cl_2_ (1:1:0.5) extract	An inhibitory effect against superoxide anion generation in fMLF/CB-stimulated human neutrophils; spongenolactone A (**282**) was more active against the growth of Staphylococcus aureus than spongenolactone B (**283**).	[87]
	**285**–**289**	Sponalactone (**285**), 17-O-acetylepispongiatriol (**286**) and 17-O-acetylspongiatriol (**287**), together with two novel spongian diterpene artifacts, namely 15α,16α-dimethoxy-15,16-dihydroepispongiatriol (**288**) and 15α-ethoxyepispongiatriol-16(15H)-one (**289**)	The South China Sea sponge *Spongia officinalis*	95% EtOH extract	Moderate inhibitory activities against LPS-induced NO production in RAW264.7 macrophages, with IC_50_ values of 12–32 µM.	[88]
	**290** and **291**	Ceylonamides A and B	the Indonesian marine sponge *Spongia ceylonensis*	EtOH extract	Significant inhibition of RANKL-induced osteoclastogenesis in RAW264 macrophages, with IC_50_ values of 13 µM and 18 µM, respectively.	[89,90]
	**297**	17-dehydroxysponalactone	Red Sea sponge *Spongia* sp.	EtOAc/MeOH/CH_2_Cl_2_ (1:1:0.5) extract	No cytotoxicity but strong inhibitory activity against the superoxide anion generation and elastase release in the fMLF/CB-induced neutrophils.	[91]
	**298**–**302**	Raspadiene (**298**), kerlinic acid (**299**), kerlinic acid methyl ester (**300**), annonene (**301**), and 6-hydroxyannonene (**302**)	Marine sponge *Raspailia bouryesnaultae*	Ethanol extract	Moderate cytotoxic activity against the human cancer cell line A549, with IC_50_ values lower than 25 µM; Compound 286 exhibits inhibitory activities against HSV-1 (KOS and 29R strains) replication by 83% and 74%, respectively, which proved that it may be a promising compound against herpes simplex virus type 1 (HSV-1, KOS, and 29R strains).	[92]
	**303** and **304**	2oxoagelasines A and F	marine sponge *Agelas nakamurai Hoshino*	EtOH extract	Inhibition against the growth of *Mycobacterium smegmatis* with inhibition zones of 10 mm at 20 µg/disc.	[93]
	**305**	10-hydro-9-hydroxyagelasine F	marine sponge *Agelas nakamurai Hoshino*	EtOH extract	Significant activities against *M. smegmatis*.	[93]
	**306**	(-)-Agelamide D	marine sponge *Agelas* sp.	Methanol (1 L × 2) and dichloromethan-e (1 L × 1) extract	Activity toward tumor growth inhibition by radiation without systemic toxicities and enhanced radiation-induced ATF4 expression and apoptotic cell death.	[94]
	**307**–**310**	Compounds **307**–**310**	marine sponge *Dysidea* cf. arenaria	Acetone (1 L) extract	Cytotoxicity against NBT-T2 cells, with IC_50_ values of 3.1, 1.9, 8.4, and 3.1 µM, respectively.	[95]
	**311**–**317**	26-O-ethylstrongylophorine-14 (**311**), 26-O-methylstrongylophorine-16 (**312**) and strongylophorines-2 (**313**), -3 (**314**), -8 (**315**), -15 (**316**), and -17 (**317**)	The Okinawan marine sponge *Strongylophora strongilata*	Ethanol extract	Inhibition against protein tyrosine phosphatase 1B (PTP1B) with IC_50_ values of 8.7, 8.5, >24.4, 9.0, 21.2, 11.9, and 14.8 lM, respectively.	[96]
	**318**	Gagunin D (GD)	marine sponge *Phorbas* sp.	/	Cytotoxicity against human leukemia cells, suppressing the expression of tyrosinase and increasing the degradation rate of tyrosinase, and inhibition activity against tyrosinase enzymatic.	[97]
	**319** and **320**	Hipposponlachnins A and B	marine sponge *Hippospongia lachne*	95% EtOH extract	Inhibition activity against the release of biomarker β-hexosaminidase and the production of pro-inflammatory cytokine IL-4 and lipid mediator LTB4 in DNP-IgE stimulated RBL-2H3 cells.	[98]
	**321**	Tedanol	the Caribbean sponge *Tedania ignis*	MeOH and CHCl_3_ extract	Great anti-inflammatory activity at 1 mg/kg in the mouse model of inflammation in vivo, and potent reduction of the carrageenan-induced inflammation in acute (4 h) and subchronic (48 h) phases.	[99]
	**325**–**327**	(+)-8-epiagelasine T (**325**), (+)-10-epiagelasine B (**326**), and (+)-12-hydroxyagelasidine C (**327**)	sponge *Agelas citrina*	CH_3_OH-CH_2_Cl_2_ (1:1, 3 × 1.5 L) extract	Compound **326** exhibited the most activity against the Gram-positive pathogens (*Staphylococcus aureus*, *Streptococcus pneumoniae*, *Enterococcus faecalis*) with an MIC in the range of 1–8 µg/mL, while other compounds showed lower activities.	[101]
Coral	**328**–**330**	Mililatensols A–C	soft coral *Sarcophyton mililatensis*	Acetone extract	Great activities in the preliminary virtual screening of inhibitory potential against SARS-CoV-2.	[102]
	**335**	Sarboettgerin E	the South China Sea soft coral *Sarcophyton boettgeri*	Acetone extract	Significant anti-neuroinflammatory activity against LPS-induced NO release in BV-2 microglial cells.	[103]
	**339**	Sarcoconvolutum D	the red sea soft coral *Sarcophyton convolutum*	Ethyl acetate extract	Cytotoxic activity against cell lines A549 and HSC-2 with IC_50_ values of 49.70 and 53.17 µM, respectively.	[104]
	**343**	Waixenicin A	soft coral *Sarcothelia edmondsoni*	/	Reduces hypoxic-ischemic brain injury and preserves long-term behavioral outcomes in mouse neonates.	[106]
	**354** and **355**	Sinupol (**354**) and sinulacetate (**355**)	Xisha soft coral *Sinularia polydactyla*	Acetone extract	Notable activity against protein tyrosine phosphatase 1B (PTP1B).	[109]
	**356**	Compound **356**	soft coral *Sinularia* sp.	MeOH (3 × 400 mL) and a butanol:CH_2_Cl_2_: H_2_O (150:50:100 mL) extract	Inhibition of the growth of three human tumor cell lines (SF-268, MCF-7, and H460) with a GI_50_ value of 70–175 µM	[110]
	**358**–**361**	Lobocrasols A–D	soft coral *Lobophytum crassum*	MeOH extract	Compounds **358** and **359** showed potent inhibition against TNFa-induced NF-jB transcriptional activity in HepG2 cells in a dose-dependent manner (IC_50_ = 6.30 ± 0.42, 6.63 ± 0.11 lM); decreased the gene expression levels in HepG2 cells in cyclooxygenase-2 (COX-2) and inducible nitric oxide synthase (iNOS) to inhibit transcription.	[111]
	**362** and **368**	Locrassumins A (**362**) and G (**368**)	soft coral *Lobophytum crassum*	95% EtOH extract	Moderate inhibition against lipopolysaccharide (LPS)-induced nitric oxide (NO) production with IC_50_ values of 8–24 µM.	[112]
	**380**–**383**	Briarellin T (**380**), asbestinin 27 (**381**) and asbestinin 28 (**382**), asbestinin 17 (**383**)	octocoral *Briareum asbestinum*	N-hexane, ethyl acetate, and methanol extract	Well-proven anti-inflammatory activity through downregulation of the pro-inflammatory cytokines TNF-α, IL-6, IL-1β, and IL-8 as well as reduction of COX-2 expression in LPS-induced THP-1 macrophages.	[115]
	**384**	Excavatolide B	Formosan gorgonian *Briareum excavatum*	Methanol and dichloromethan-e (1:1) extract	Significant inhibition against the mRNA expression of the proinflammatory mediators, inducible nitric oxide synthase (iNOS) and cyclooxygenase-2 (COX-2), in lipopolysaccharide (LPS)-challenged murine macrophages (RAW 264.7); deaden carrageenan-induced nociceptive behaviors, mechanical allodynia, thermal hyperalgesia, weight-bearing deficits, and paw edema; inhibitory activity against iNOS and the infiltration of immune cells in carrageenan-induced inflammatory paw tissue.	[116]
	**385** and **386**	Sangiangol A and B	soft coral *Anthelia* sp.	EtOH extract	Moderate cytotoxicity against an NBT-T2 cell line (0.5–10 µg/mL).	[117]
	**387**–**389**	cespitulins H-J	soft coral *Cespitularia* sp.	EtOAc extract	Great anti-inflammatory activities; inhibition of the production of TNF-α and NO; suppression of the expression of iNOS and COX-2 gene.	[118]
	**399, 401**–**403**	Simplexins P (**399**) and R and S (**401** and **402**), simplexin A (**403**)	soft coral *Klyxum simplex*	EtOAc extract	Cytotoxicity against a limited panel of cancer cell lines.	[119]
	**409** and **411**	lobocrasol A and C	Vietnamese soft corals	/	Activities against bloodstream forms of *T. brucei*; elective activity against *L. donovani.*	[120]
	**404, 405, 409, 411, 417, and 420**	Compounds **404, 405,** and lobocrasol A (**409**), lobocrasol C (**411**), sinumaximol C (**417**), and 13-Epi-scabrolide C (**420**)	Vietnamese soft corals	/	Activities against bloodstream forms of *T. brucei.*	[120]
Sea hare	**434**–**438**	parguerol (**434**), parguerol 16-acetate (**435**), deoxyparguerol (**436**), isoparguerol (**437**), and isoparguerol 16-acetate (**438**)	sea hare *Aplysia*. *Dactylomela*	Chloroform-methanol (2:1) extract	Inhibition against P388 murine leukemia cells (IC_50_ = 8.3, 8.6, 0.86, 10.1, 1.0 µM).	[121]
	**442** and **443**	Compounds **442** and **443**	sea hare *Aplysia pulmonica*	95% EtOH extract	Toxicity against *Artemia salina* at a concentration of 0.5 µM.	[122]

**Table 3:** Marine plant-derived compounds with various bioactivities.

Source	NO.	Compound	Producing Organism	Extract/Fraction	Activity	References
Red algae	**444** and **445**	15-bromo-2,7,16,19-tetraacetoxy-9(11)-parguerene (**444**) and 15-bromo-2,7,16-tetraacetoxy-9(11)-parguerene (**445**)	the marine red algae *Laurencia obtusa* (Hudson) *Lamouroux*	/	Cytotoxicity.	[128]
	**446, 450, 451, 453**	Compounds 446, 450, 451, 453	the marine red algae *Laurencia obtusa* (Hudson) *Lamouroux*	/	Cytotoxic activity against HeLa with IC_50_ values of 5.7, 0.68, 10.8, and 11.6 µM, respectively, and against P388 cell lines with IC_50_ values of 6.5, 2.5, 14.6, and 18.3 µM, respectively.	[128]
	**463**	Sphaerodactylomelol	the red algae *Sphaerococcus coronopifolius*	MeOH and CH_2_Cl_2_ extract	Antimicrobial activity against S. aureus with IC_50_ value of 96.3 µM; showed cytotoxicity to HepG-2 cells with IC50 value of 720 µM; induced inhibition of cell proliferation with IC_50_ value of 280 µM.	[131]
	**468** and **471**	Compounds **468** and **471**	the red algae *Sphaerococcus coronopifolius*	CH_2_Cl_2_/MeOH (3/1) extract	Antitumor activity on one murine cancer cell line (one murine cancer cell line, B16F10, and five human cancer cell lines, A549, Hs683, MCF7, U373) with IC_50_ values 15 and 16 µM, respectively, and doxorubicin used as a positive control.	[132]
	**495**–**497**	Neorogioltriol (**495**), neorogioldiol (**496**), and O^11^,15-cyclo-14-bromo-14,15-dihydrorogiol-3,11-diol (**497**)	the red algae *Laurencia*	CH_2_Cl_2_/MeOH extract	Suppressed macrophage activation and promoted an M2-like anti-inflammatory phenotype.	[140]
Brown algae	**454** and **455**	Compounds **454** and **455**	the marine brown algae *Dictyota pfaffii*	CH_2_Cl_2_ extract	Greater anti-HIV-1 activities than compound **457**, with IC_50_ values of 2.9 and 4.1 µM, while its cytotoxic activity against MT-2 lymphocyte tumor cells was lower.	[129]
	**473**	Compound **473**	the brown seaweed *Bifurcaria bifurcate*	CH_2_Cl_2_/MeOH extract	Inhibition against the growth of cancer cells (78.8%) at 100 µg/mL test concentration.	[133]
	**476**–**481**	Compounds **476**–**481**	the brown seaweed *Bifurcaria bifurcate*	CH_2_Cl_2_/MeOH extract	Inhibiton against the growth of the MDA-MB-231 cell line with IC_50_ values ranging from 11.6 to 32.0 µg/mL.	[134]
	**482** and **483**	Eleganolone and eleganonal	the brown seaweed *Bifurcaria bifurcate*	CH_2_Cl_2_/MeOH extract	Antioxidant potential by FRAP and ORAC assays.	[135]
	**484**	Bifurcatriol	the brown seaweed *Bifurcaria bifurcate*	CH_2_Cl_2_/MeOH extract	High activity against the malaria parasite *P. falciparum* (IC_50_ = 0.65 µg/mL) with low cytotoxicity (IC_50_ = 56.6 µg/mL).	[136]
	**485a**	Pachydictyol B	the brown algae *Dictyota dichotoma*	Dichloromethan-e extract	Weak antimicrobial properties.	[138]
	**487**–**489**	Pachydictyol A (**487**), isopachydictyol A (**488**), and dichotomanol (**489**)	the brown algae *Dictyota dichotoma*	Dichloromethan-e extract	Useful to the studies of more active antithrombotic prototypes.	[137]
	**490**–**494**	Compounds **490**–**494**	the *Dictyota* brown algae	95% EtOH extract	Potent antioxidant activities against H2O2-induced oxidative damage in neuron-like PC12 cells at a low concentration of 2 µM.	[139]
Another algae	**498** and **499**	Enhoidin A and B	Tropical seagrass *Enhalus acoroides*	95% ethyl alcohol extract	Moderate cytotoxic activities against four human cancer cell lines (MCF-7, HCT-116, HepG-2, and HeLa).	[141]
	**468**–**470**	Compounds **468**–**470**	Jamaican macroalga *Canistrocarpus cervicornis*	Hexane, methylene chloride, ethyl acetate, and methanol extract	Moderate and concentration-dependent cytotoxic activity against human tumor cell lines PC3 and HT29.	[130]
Mangrove	**502** and **503**	Tagalons C and D	the Chinese mangrove *Ceriops tagal*	95% EtOH extract	Selective cytotoxicities with IC_50_ values of 3.75 and 8.07 µM against the human breast cancer cell line MT-1.	[142]
	**504**	Isopimar-8(14)-en-16-hydroxy- 15-one	Maruhubi mangrove *Ceriops tagal*	Chloroform extract	Antibacterial activity against *Bacillus cereus*, *Staphylococcus aureus*, and *Micrococcus kristinae* (each with MIC values of 100 µg/mL); activity towards *Streptococcus pyrogens* and *Salmonella pooni* (MIC = 500, 250 µg/mL), with chloramphenicol serving as the positive control (each with MIC values of 1.0 µg/mL).	[143]
	**506**	Compound **506**	marine mangrove *Bruguiera gymnorrhiza*	/	Moderate cytotoxicity against K562 chronic myeloid leukemia cells with an IC_50_ value of 22.9 µM.	[144]
	**510**	Compound **510**	the stems of marine mangrove *Bruguiera gymnorrhiza* from Xiamen China	/	Weak cytotoxicity on L-929 (IC_50_ = 30.6 µM)	[144]

## Data Availability

Not applicable.

## Abbreviations

Abbreviation	Full Name
A2780CisR	Cisplatin-resistant human ovarian cancer cells
A-549	Human non-small cell lung cancer cells
BEL-7402	Human liver cancer cells
BuOH	Butanol
COX-2	Cyclooxygenase-2
ECD	Electronic circular dichroism
EtOAc	Ethyl acetate
GM-CSF	Granulocyte-macrophage colony stimulating factor
HepG2	Human hepatocelular carcinomas
HL-60	Human promyelocytic leukemia cells
HPLC	High-performance liquid chromatography
HR-ESI-MS	High-resolution electrospray ionization mass spectroscopy
HUVEC	Human umbilical vascular endothelial cell
IC50	Half maximal inhibitory concentration
IgE	Immunoglobulin E
IL-13	Interleukin-13
IL-1β	Interleukin-1beta
iNOS	Inducible nitric oxide synthesis
LC-MS	Liquid chromatograph mass spectrometer
LPS	Lipopolysaccharide
MCF-7	Human breast adenocarcinoma cell line
MCP-1	Monocyte chemoattractant protein-1
MeOH	Methanol
MIC	Minimum inhibitory concentration
MIP-1β	Macrophage inflammatory protein
MOE	Murine oviductal epithelial
MOSE	Murine ovarian surface epithelial
MTT	Methyl thiazolyl tetrazolium
n-BuOH	N-Bromosuccinimide
NCI-H460	Human non-small cell lung cancer cell line
NF-kB	Nuclear factor-kappa B
NHDF	Normal human dermal fibroblasts
NMR	Nuclear magnetic resonance spectroscopy
p.c.	Positive control
PE	Polyethylene
PTP1B	Protein tyrosine phosphatase
RBL-2H3	Rat basophilic leukemia cells
ROS	Reactive oxygen species
SF-268	Human glioma cell line
TB	Tuberculosis
TNF-α	Tumor necrosis factor-α
VEGF-A	Vascular endothelial growth factor A
